# Determination of modifiable risk factors for length-for-age z-scores among resource-poor Indonesian infants

**DOI:** 10.1371/journal.pone.0247247

**Published:** 2021-02-18

**Authors:** Aly Diana, Jillian J. Haszard, Sri Y. Irda Sari, Sofa Rahmannia, Annisha Fathonah, Wina Nur Sofiah, Haidar Rizqi, Raulia Haekal, Allissia Gilmartin, Michelle Harper, William Petri, Lisa Houghton, Rosalind Gibson

**Affiliations:** 1 Faculty of Medicine, Nutrition Working Group, Universitas Padjadjaran, Bandung, Indonesia; 2 Department of Human Nutrition, University of Otago, Dunedin, New Zealand; 3 Faculty of Medicine, Department of Public Health, Universitas Padjadjaran, Bandung, Indonesia; 4 Faculty of Medicine, Universitas Pasundan, Bandung, Indonesia; 5 Department of Microbiology, Immunology and Cancer Biology, University of Virginia, Charlottesville, Virginia, United States of America; Curtin University, AUSTRALIA

## Abstract

To reduce the burden of early-life linear growth faltering in low- and middle-income countries, interventions have focused on nutrition strategies, sometimes combined with water quality, sanitation, and hygiene (WASH). However, even when combined, their effects on linear growth have been inconsistent. Here, we investigate potential predictors of length-for-age z-scores (LAZ) in a cohort of resource-poor rural Indonesian infants to inform the optimal strategies to reduce linear growth faltering. Apparently healthy rural breastfed Indonesian infants were randomly selected from birth registries at age 6 months (n = 230) and followed up at 9 (n = 202) and 12 (n = 190) months. Using maximum likelihood estimation, we examined longitudinal relationships among socio-demographic status, maternal height, infant sex, age, water source, sanitation facility, energy, protein, micronutrient intakes and biomarkers (serum ferritin, zinc, retinol binding protein (RBP), selenium–adjusted for inflammation), and α-1-acid glycoprotein (AGP) and C-reactive protein (CRP) (systemic inflammation biomarkers) at age 6 and 9 months on LAZ at age 9 and 12 months. Stunting (LAZ <-2) at 6, 9, and 12 months was 15.7%, 19.3%, and 22.6%, respectively. In the full model, the predictor variable at age 6 months that was most strongly associated with infant LAZ at 9 months was maternal height (0.18 (95% CI 0.03, 0.32) SD). At age 9 months, the strongest predictors of LAZ at 12 months were improved drinking water source (-0.40 (95% CI -0.65, -0.14) vs. not improved), elevated AGP compared to not elevated (0.26 (95%CI -0.06, 0.58), maternal height (0.16 (95% CI 0.02, 0.31) SD), sex (0.22 (95% CI -0.02,0.45) female vs. male), serum RBP (0.12 (95% CI -0.01, 0.25) SD), and protein intake (0.17 (95% CI -0.01, 0.35) SD). Health promotion that includes exclusive breastfeeding up to the first six months and follows microbial water quality guidelines to ensure water intake is always safe should be considered.

## Introduction

Multiple factors influence linear growth during early childhood, many of which contribute to the burden of stunting, especially in low resource settings. The World Health Organization (WHO) has set a global target to reduce stunting worldwide by 40% in 2025 to reduce the impairments associated with linear growth retardation [1]. These impairments may include delays in cognitive and motor development which can persist into adulthood [2].

Unfortunately, the impact of complementary feeding interventions on linear growth during infancy and early childhood (i.e., age 6–24 month) has yielded mixed results [3], stimulating investigations of other potential pathways linked to stunting. In low and middle-income countries, interventions focusing on the influence of the individual or combined effects of water quality, sanitation, and hygiene (WASH) on child growth [4], sometimes in conjunction with nutrition-specific strategies [5,6], have been investigated. However, even when combined, their effects on linear growth during infancy and early childhood have been inconsistent [5,6].

Indonesia is a country with the fifth highest burden of stunted children in the world [7], yet there has been a negligible change in the stunting prevalence in the last decade. Almost one-third (31%) of children below five years of age were stunted in 2018, with large disparities across provinces, ranging from 18% in Jakarta to 43% in East Nusa Tenggara [8]. These discrepancies emphasize the urgent need to investigate the etiology of growth retardation in Indonesia sub-nationally, so that effective intervention strategies can be implemented. Consequently, several investigators have explored the determinants of growth retardation among infants and young children in Indonesia; these studies have been reviewed extensively before [9]. However, because many of these studies were cross-sectional, they were unable to determine the direction of relationships. Moreover, to our knowledge, none have investigated the combined effects of nutrient intakes and WASH variables on linear growth during infancy and early childhood.

Data from our earlier comprehensive Indonesian cohort study [10,11] has provided a unique opportunity to explore both individually and combined the influence of nutrient intakes and WASH variables on the linear growth of rural infants from Sumedang district, West Java. This district is one of the 100 (from a total of 514) districts and cities in Indonesia in which interventions to combat stunting are considered a priority by the Indonesian government [12].

Earlier we developed a nutrient quality score using principal component analysis and showed that infants from Sumedang district with a higher nutrient quality score at age 9 months had a lower risk at age 12 months of being stunted after adjusting for confounding variables [13]. Here, we have used maximum likelihood estimation applied using structural equation modeling on this longitudinal data to investigate which of the following − nutrient intake, maternal and WASH measures, and/or micronutrient biomarkers − predict length-for-age z-scores (LAZ) among these rural infants from Sumedang district in the first year of life. Our aim was to broaden our understanding of the etiological factors influencing linear growth in this rural setting in an effort to identify the optimal combination of interventions needed to reduce linear growth faltering among these resource-poor infants.

## Methods

### Study setting and participants

Details of the study site, recruitment, and sociodemographic, health and anthropometric data collected in this prospective cohort study from August 2014 to August 2015 have been reported earlier [10]. Briefly, mother infant dyads (n = 230) were enrolled at 6 months postpartum from August 2014 to February 2015 following random selection from all the villages (n = 30) in the three sub-districts of Sumedang district, West Java, Indonesia. In addition, stool samples were also collected from a sub-sample of infants at age 12 months to investigate the presence of intestinal inflammation. Sumedang district is located about 50 km from Bandung City (capitol of West Java) and has a population of 1.1 million and an area of ~ 152 km squares. The climate is tropical with rainfall during most months, although often heavier from October to February, with a short dry season, generally from March to September. Approximately 22% of the area is used for paddy plantation [14].

Inclusion criteria were apparently healthy full term (≥ 37 weeks gestation), breast fed infants weighing at least 1500 g at birth. Of the eligible infants (n = 275), caregivers of 230 infants agreed to allow their infants to participate (84% response rate) in the study. Infants were followed up at age 9 (n = 202) and 12 (n = 190) months. In our initial study the sample size was powered to estimate the prevalence of stunting (LAZ < -2SD) with a 95% confidence interval precision of at most 7%.

The prospective cohort study protocol was approved by the Human Ethics Committee of Padjadjaran University, Indonesia (No 132/UN6C2.1.2/KEPK/PN/2014 and the University of Otago, New Zealand (H14/022). A follow-up study was conducted approximately 54−60 months later to collect water samples from the households. This follow-up study was also approved by the Human Ethics Committee of Padjadjaran University, Indonesia (No. 863/UN6.KEP/EC/2019). Written informed consent was provided by the parents or primary guardians of the infants and all participants were free to withdraw from the initial and follow-up study at any time.

### Socio-demographic, health, and anthropometric measurements

Information on sociodemographic characteristics, infant immunization status, household water and sanitation facilities, maternal hand washing, administration of worm medication and vitamin A supplements was collected in the prospective cohort study during home visits by the field doctors using a structured interviewer-administered questionnaire. Socio-economic status (SES) of the households was measured by an asset-based wealth index calculated using principal component analyses described earlier.

We applied the operational definitions used by WHO and UNICEF [15] for “improved” sources of drinking water and sanitation facilities. A drinking water source was considered “improved” when by nature of its construction or through active intervention it was protected from outside contamination, in particular with fecal matter. The household sanitary facility was considered “improved” if it consisted of a flush toilet, piped sewer system, septic tank and flush/out flush to a pit latrine. A handwashing score was created based on the number of occasions the mother in the household performed handwashing after going to the toilet, after changing a diaper, before cooking, eating, feeding her infant, and after handling raw food and trash, with a total score of seven. Maternal height, and infant length were measured using standardized techniques and calibrated equipment [10] from which infant LAZ were calculated at 6, 9, and 12 months of age using the WHO growth reference data [16]. Infant LAZ were all within the biological plausible range set by WHO Multicentre Growth Reference Study Group [16]. The proportion of mothers with height less than 145 cm was determined in view of the evidence that the offspring of mothers shorter than 145 cm have an increased risk of mortality, underweight, and stunting [17].

### Assessment of nutrient intakes from complementary foods and biomarkers of micronutrients, inflammation, helminthiasis, and environmental enteric dysfunction

Briefly, at 6 and 9 months of age, two in-home weighed records of complementary food intakes on non-consecutive days were collected from which energy and nutrient intakes were calculated using a specially compiled food composition database [18]. Next, the Multiple Source Method (MSM) was used to determine the “usual” intakes of energy and selected nutrients [19]. Nutrient quality index scores were calculated from eight correlated nutrients from complementary foods at 6 and 9 months of age (vitamin A, ascorbic acid, thiamine, riboflavin, niacin, calcium, iron and zinc) using principal components analyses as described earlier [13].

Morning non-fasting venipuncture blood samples were drawn from the infants at age 9 months for analyses of serum ferritin, zinc, selenium, retinol binding protein (RBP), C-reactive protein (CRP) and α-1-acid glycoprotein (AGP). Some results of biomarkers were missing at random, mostly because blood collected from some participants was not enough for all analysis. The number of missing values for each micronutrient biomarker has been reported previously [11]. Serum biomarkers were adjusted for inflammation using both CRP and AGP [20]. Serum zinc was adjusted for time of day and the time lapse since last feeding prior to the blood collection, and then adjusted for inflammation [21]. Anemia was defined as Hb < 110 g/L [22]. Suboptimal serum biomarkers (adjusted for inflammation) were as follows: ferritin < 12 μg/L [22]; RBP < 0.83 μmol/L [23]; zinc < 9.9 μmol/L [24]; and selenium < 0.82 μmol/L [25]. Systemic inflammation was assessed by serum C-reactive protein (CRP) > 5mg/L and α-1-acid glycoprotein (AGP) > 1 g/L [26].

Stool samples were collected from the infants at 6, 9, and 12 mo of age, and transported at– 4°C in chilled containers to the Parasite Laboratory, Faculty of Medicine, Universitas Padjadjaran, Indonesia. The Kato Katz method [27] was used for microscopic detection of the absence/presence of soil- transmitted helminths. An aliquot of the stool samples collected at age 12 months from a sub-sample of infants (n = 75) were frozen at -80°C without fixative, and subsequently assayed for myeloperoxidase (MPO), a marker for intestinal inflammation, in the laboratory of WP. Commercially available enzyme-linked immunosorbent assays (ELISAs) were used following instructions on the package insert (Alpco, Salem, NH), except that the initial dilutions run were 1:500 [28]. Plates were read on a fluorescence plate reader (BioTek). The final MPO data were corrected for the dilution factor in the assay.

### Collection and detection of *E*. *coli* and other coliform bacteria in water samples during household follow-up

Of the 230 households in the initial prospective cohort study, 175 agreed to participate in the follow-up study which was prompted by the findings from the maximum likelihood estimation based on the earlier cohort data. In the follow-up study, household water samples (100 mL) were collected in sterile containers from the main sources of drinking water at both the point of collection and point of use during the months of May and August 2019 (dry season). All water samples were stored immediately in a cooler, and transported on ice to the base laboratory for processing within 6–8 hours after collection.

Details of improved or unimproved drinking water were matched to a specific water source type according to the classification used in the Indonesian Demographic and Health Survey (DHS) [29]. Information on boiling water at point of use was based on maternal self-reports.

Bacterial identification was conducted using a membrane filtration technique (nitrocellulose membrane filter: 47 mm in diameter, pore size: 0.45 *μ*m, Merck Millipore) with Chromocult *Coliform* agar as the culture media (Merck Millipore, Jakarta, Indonesia) in the accredited Microbiology and Parasitology Laboratory of the Faculty of Medicine, Universitas Padjadjaran, Indonesia. Briefly, after filtering the water samples (100 mL) through the membrane filters, samples were placed on the culture media and incubated at 37°C for 24 hours until colonies formed. *E*. *coli* were identified by colony counting expressed in colony-forming units (CFU) of *E*.*coli* per 100mL water sample, and results compared with the criteria set for drinking water of 0 CFU/100 mL *E*. *Coli* [30]. Full details of this method are reported elsewhere [31].

### Statistical analysis

Variables to be investigated as predictors of LAZ were selected *a priori* and were known or hypothesized to influence LAZ based on our earlier findings of resource-poor rural infants, as well as theoretical evidence from the literature. LAZ was treated as a continuous variable rather than dichotomized to minimize the loss of information, maximize the statistical power, preserve the variation in the outcome, and avoid residual confounding.

LAZ is the primary outcome and was measured at 6, 9 and 12 months of age. There were three groups of predictors: demographic predictors (which included maternal predictors and handwashing practices measured at 6, 9 and 12 months of age); nutrient intake predictors (at 6 and 9 months of age); and biomarker predictors of micronutrients and systemic inflammation (at 6 and 9 months of age). Each of these groups of predictors was assessed against LAZ at 9 and 12 months separately before generating a complete model with all relevant predictors. This was to allow the comparison of ‘unadjusted’ and ‘adjusted’ estimates of association.

To estimate relationships between predictors and LAZ, dynamic panel data models were fitted using maximum likelihood estimation within structural equation models, using the xtdpdml command in Stata 16.1 (StataCorp, Texas) [32]. This method was selected for its ability to use longitudinal data with lagged predictors while accounting for unobserved confounders and it has been shown to be more efficient than other methods [32]. Full information maximum likelihood was used for missing data and robust standard errors were calculated. For comparison of effect sizes, all continuous predictors were standardized to be in units of standard deviations. For completeness and because standardized variables in SEM may affect the standard errors [33], unstandardized estimates were also calculated and presented. However, it is the standardized coefficients that are used to answer the primary research question and limited dependence on p-values is used for interpretation [34]. All models were adjusted for LAZ at the previous age. Goodness-of-fit was assessed by RMSEA (ideally less than 0.06); CFI and TLI (both ideally greater than 0.95) [35] and lagged predictors were free to vary with time. Regression coefficients, 95% confidence intervals, and p-values were calculated.

In the assessment of demographic predictors of LAZ (at 6, 9 and 12 months), the time-invariant predictors of wealth score, maternal height and education, improved drinking source, improved sanitation, and sex of infant were assessed from baseline measures. Handwashing practices were included as a time-varying predictor. As the relationships between demographic predictors and LAZ were not substantially different at each age, estimates were constrained across time. This means that associations represent the average association between the predictor and LAZ at each age.

The relationships between nutrient intake and LAZ were assessed using lagged predictors and estimates at each age were reported separately. To understand how the different measures of nutrient intake were related to LAZ, three different models were run: (1) energy intake and nutrient quality score as predictors; (2) energy intake and protein intake as predictors; and (3) energy, calcium, iron, zinc, retinol, and riboflavin intake as predictors. The regression coefficients for nutrient quality score were compared with those when protein intake was included in the model instead of nutrient quality (as nutrient quality and protein intake are correlated so only one should be included) and the strongest predictor of these was taken to be used in the full model with all predictors.

The relationships between biomarkers (serum ferritin, zinc, RBP, selenium, CRP, and AGP) and LAZ were assessed using lagged predictors and estimates at each age were reported separately. AGP was transformed into categorical variable using > 1 g/L cut-off, as it was not normally distributed even after log-transformation.

The full model of all predictors (demographics, nutrient intake, and biomarkers) was generated with time-invariant demographic predictors, and all other variables lagged and allowed to vary freely with time. Estimates for 6 months predicting 9 months were reported, and 9 months predicting 12 months.

## Results

### Selected socio-demographic and anthropometric status of infants and their mothers

A total of 230 6-month old infants (53.5% female) were enrolled in the study with a completion rate of 82.6% (Table 1). Of the mothers, 15.5% had a height < 145 cm, with an average height of 150.2 cm (SD 5.2 cm). Most of the mothers were housewives (91.6%), and more than 60% of the fathers were manual laborers, farmers, or were unemployed without a regular income. More than 50% of the mothers and fathers had completed secondary school.

**Table 1:** Maternal, socio-economic, infant, and household characteristics.

**Maternal characteristics**	n	
Age, mean (SD) years	226	27.5 (7.2)
Height, mean (SD) cm	225	150.2 (5.2)
Height < 145 cm %	35/226	15.5
Education, %		
Primary school or less	93/229	40.6
Secondary school	122/229	53.3
College/university	14/229	6.1
Wealth index, median (IQR)	223	0.24 (-1.62 to 1.40)
**Infant characteristics**		
Sex, female (%)	123/230	53.5%
**Household characteristics**		
Father’s education, %		
Primary school or less	109/229	46.7
Secondary school	108/229	47.2
College/university	12/229	25.3
Father’s occupation, %		
Regular wage earner	22/228	9.6
Business or trade owner	69/228	30.3
Manual labor	100/228	43.9
Farmer	26/228	11.4
Unemployed	11/228	4.8
Improved drinking-water source^b^, %	133/212	62.7
Improved sanitation facility^c^, %	174/214	81.3

SD: Standard deviation; IQR: Interquartile range.

^a^Includes water sources such as piped water, tube well/borehole, protected dug well, protected spring.

^b^Includes flush or pour-flush to piped sewer system, septic tank or pit latrine, ventilated improved pit latrine, pit latrine with slab and composting toilet, unshared with other households.

More than half of the households (107/209, 51.2%) had both a drinking-water source and toilet facility classified as “improved”. For almost all the infants (i.e., 96.8%), birthweight was between 2500–4500 g; none of the infants had a birthweight >4500 g, as measured within 1–3 days by the midwives or health personnel.

At age 9 months, 50% of the infants received vitamin A supplements, increasing to more than 70% by 12 months of age, a time when almost all infants were also fully immunized (almost 90%) (Table 2). The mean LAZ of the infants declined with increasing age, with the proportion classified as stunted increasing from 15.7% at 6 months, 19.3% at 9 months, to 22.6% at 12 months of age [10].

**Table 2:** Health, anthropometric characteristics, and nutrient intakes at age 6, 9, and 12 months.

	n	6 months	n	9 months	n	12 months
Vitamin A supplementation	36/220^a^	16.4%	104/192^b^	54.2%	134/186^b^	72.0%
Complete immunizations^c^					165/185	89.2%
Hand washing^d^, median (IQR)	225	2 (1 to 3)	198	3 (2 to 6)	186	4 (2 to 7)
LAZ, mean (SD)	225	-1.02 (0.96)	202	-1.16 (0.97)	190	-1.26 (1.00)
LAZ <-2 SD	35/225	15.6%	39/202	19.3%	43/190	22.6%
Energy, median (IQR) kcal/d	214	128 (95 to 166)	192	275 (218 to 378)	186	396 (302 to 492)
Protein, median (IQR) g/d	214	3.7 (2.8 to 5.1)	192	7.9 (5.6 to 11.9)	186	12.4 (8.7 to 16.9)
Calcium, median (IQR) mg/d	214	83 (55 to 116)	192	109 (69 to 144)	186	122 (85 to 170)
Iron, median (IQR) mg/d	214	3.1 (1.9 to 4.2)	192	2.4 (1.5 to 3.4)	186	2.7 (1.9 to 3.7)
Zinc, median (IQR) mg/d	214	1.2 (0.8 to 1.7)	192	1.5 (1.0 to 2.1)	186	2.0 (1.5 to 2.7)
Vitamin A RAE, median (IQR) μg/d	214	(56 to 115)	192	53 (25 to 105)	186	57 (26 to 99)
Riboflavin, median (IQR) mg/d	214	0.09 (0.06 to 0.12)	192	0.15 (0.09 to 0.30)	186	0.26 (0.17 to 0.42)
Nutrient quality score, mean (SD)	209	0.0 (2.3)	195	0.0 (2.1)	189	0.0 (2.2)

SD: Standard deviation; IQR: Interquartile range; LAZ: Length-for-age z-scores; RAE: Retinol activity equivalents.

^a^In the last 6 months.

^b^In the last 3 months.

^c^At 12 months (at least 1 BCG, 1 Polio, 1 DPT, 1 Hepatitis B, and 1 Measles (taken from immunization card).

^d^Hand washing score was calculated from number of occasions mother performed hand washing (after going to toilet, after changing diaper, before cooking, before feeding infants, before eating, after handling raw food, and after holding trash).

### Intakes of energy and selected nutrients from complementary foods at 9 months of age

All infants were predominantly/exclusively breastfed up to 6 months of age with continued breastfeeding up to 12 months of age except for four infants who were no longer being breastfed. Almost all of the infants predominantly breastfed at age 6 months consumed iron-fortified foods consisting of fortified cereal-based porridges and biscuits, with only a few (n = 9) consuming fortified formula milk. At 9 months of age, median energy intake from complementary foods alone was below the WHO estimated need (59.4%), whereas the intake of protein exceeded the estimated need by 75% [36]. Deficits in median intakes for several micronutrients at both 9 and 12 months in relation to the WHO estimated needs (as %) were observed, and were most notable for calcium (~ 70%) at both 9 and 12 months of age, and iron (~ 42%) and zinc at 9 months only (58%) [18].

### Biomarkers of micronutrient, inflammation, and EED status at 9 months of age

Details of the serum micronutrient biomarkers adjusted for inflammation [20] have been presented earlier [10,11]. At age 9 and 12 months, more than 35% of the infants were anemic, with 45% with low ferritin at 9 months, increasing to 65% at age 12 months (Table 3). After adjusting for inflammation, over 50% of the infants were judged to have selenium deficiency at both 9 and 12 months of age, whereas the incidence of zinc, vitamin B-12, and vitamin A deficiency was much lower at both time points, ranging from 8.3% for vitamin A (based on RBP) at 9 months to 16.4% for zinc at 9 months.

**Table 3:** Helminthiasis, MPO, biomarkers indicative of micronutrient deficiencies and inflammation at age 6, 9, and 12 months.

	n	6 months	n	9 months	n	12 months
Helminths			1/166	0.6%		
MPO, median (IQR) ng/L					75	2863 (1183 to 5309)
MPO, <2000 ng/L					44/75	58.7%
Anemia, <110 g/L	74/224	33.0%	84/199	42.2%	73/190	38.4%
Serum ferritin, <12μg/L	45/208	21.6%	87/193	45.1%	120/185	64.9%
Serum zinc, <9.9 μmol/L	26/185	14.1%	27/165	16.4%	16/154	10.4%
Serum RBP, <0.83 μmol/L	37/208	17.8%	16/193	8.3%	23/185	12.4%
Serum selenium, <0.82 μmol/L	134/185	72.4%	97/165	58.8%	81/154	52.6%
Serum CRP, > 5 mg/L	39/208	18.8%	38/193	19.7%	41/185	22.2%
Serum AGP, > 1 g/L	32/208	15.9%	43/193	22.3%	49/185	26.5%

SD: Standard deviation; IQR: Interquartile range; MPO: Myeloperoxidase; RBP: Retinol binding protein; CRP: C-reactive protein; AGP: α-1-acid glycoprotein.

At 9 months of age, only two infants had received deworming medication at some time, and less than 1% had evidence of helminthic parasites. At age 12 months, 44/75 (almost 60%) of the infants for whom a sufficient stool sample was available for assay had elevated MPO concentrations (i.e., > 2,000 ng/L) [28]. There were no significant differences in maternal/paternal education, maternal age or infant LAZ between those infants whose stools were assayed for MPO (n = 75) and those for whom no assays were performed (n = 150), with the exception of household wealth. More infants whose stool was assayed for MPO were from households with a higher wealth index (0.24 vs. -0.11).

### Predictors of infant length-for-age Z between 6 and 12 months of age

Of the seven socio-demographic and WASH predictors examined in Table 4, only improved drinking water source (-0.31 (95% CI -0.57, -0.04)) demonstrated a negative association with LAZ, and maternal height (0.21 (95% CI 0.07, 0.34)) a positive association. Three models were explored for potential nutrient predictors of infant LAZ, of which protein intakes (0.16 (95% CI 0.001, 0.32)) at 9 months was the nutrient variable most strongly and positively associated with LAZ at age 12 months (Table 5). Of the biomarker predictors of micronutrients and systemic inflammation (Table 6), serum ferritin at 6 months indicated a negative association with LAZ at 9 months, whereas elevated AGP compared to not elevated (0.26 (95% CI -0.04, 0.55)) and serum RBP at 9 months showed an association that tended to be positive (0.11 (95% CI -0.02, 0.24)) with LAZ at 12 months.

**Table 4:** Demographic predictors of length-for-age z-score (LAZ) between 6 and 12 months of age (n = 225).

	Mean difference in LAZ (95% CI)^a^ by standardized predictors	p-value	Units for unstandardized predictors	Mean difference in LAZ (95% CI)^a^ by unstandardized predictors, z-score	p-value
LAZ	0.15 (-0.31, 0.60)	0.532	z-score	0.03 (-0.43, 0.49)	0.901
Socio-economic status (wealth index)^b^	-0.02 (-0.15, 0.11)	0.796	Standardized score^b^	-0.01 (-0.08, 0.06)	0.815
Improved drinking water source^c^	-0.31 (-0.57, -0.04)	0.024	-	-0.33 (-0.61, -0.05)	0.021
Improved sanitation facility^d^	-0.02 (-0.39, 0.35)	0.908	-	0.00 (-0.41, 0.41)	0.989
Hand washing^e^	0.06 (-0.03, 0.15)	0.175	score^e^	0.02 (-0.01, 0.06)	0.169
Maternal height	0.21 (0.07, 0.34)	0.003	cm	0.04 (0.02, 0.07)	0.002
Maternal education (at least high school compared to less than high school)	-0.09 (-0.35, 0.16)	0.477	-	-0.12 (-0.39, 0.16)	0.413
Sex of infant (female compared to male)	0.12 (-0.10, 0.34)	0.272	-	0.11 (-0.13, 0.35)	0.373

Goodness of fit statistics for the standardized model: CFI = 0.99; TLI = 0.97; RMSEA = 0.050; χ^2^ (DF) = 33.56 (8).

^a^Calculated using maximum likelihood longitudinal structural equation modeling with estimates constrained across time (6, 9, and 12 months), meaning that these represent the average association between the predictor and LAZ at each age. The maternal height measure was standardized for the standardized regression coefficients.

^b^Measured by wealth index.

^c^Includes water sources such as piped water, tube well/borehole, protected dug well, protected spring.

^d^Includes flush or pour-flush to piped sewer system, septic tank or pit latrine, ventilated improved pit latrine, pit latrine with slab and composting toilet, unshared with other households.

^e^Measured at 6, 9, and 12 months. Hand washing measured as a score for the number of scenarios that mothers washed their hands.

**Table 5:** Nutrient predictors of length-for-age z-score (LAZ) at 9 and 12 months of age (n = 225).

	Mean difference in LAZ (95% CI)^a^ by standardized predictors	p-value	Units for unstandardized predictors	Mean difference in LAZ (95% CI)^a^ by unstandardized predictors, z-score	p-value
**Model 1 –nutrient quality score**					
Predictors at 6 months of age for LAZ at 9 months					
LAZ	0.17 (-0.17, 0.52)	0.330	z-score	0.05 (-0.28, 0.38)	0.760
Energy intake	0.07 (-0.05, 0.18)	0.242	10% difference^b^	0.012 (-0.009, 0.033)	0.276
Nutrient quality score	-0.02 (-0.11, 0.07)	0.690	Standardized score	0.00 (-0.04, 0.03)	0.810
Predictors at 9 months of age for LAZ at 12 months					
LAZ	0.17 (-0.17, 0.52)	0.330	z-score	0.05 (-0.28, 0.38)	0.760
Energy intake	-0.06 (-0.19, 0.06)	0.334	10% difference^b^	-0.017 (-0.045, 0.011)	0.227
Nutrient quality score	0.11 (-0.02, 0.23)	0.090	Standardized score	0.05 (0.00, 0.10)	0.062
**Model 2 –protein intake**					
Predictors at 6 months of age for LAZ at 9 months					
LAZ	0.18 (-0.17, 0.53)	0.325	z-score	0.05 (-0.28, 0.39)	0.752
Energy intake	0.00 (-0.20, 0.21)	0.963	10% difference^b^	0.006 (-0.030, 0.042)	0.757
Protein intake	0.06 (-0.15, 0.27)	0.568	10% difference^b^	0.006 (-0.030, 0.042)	0.778
Predictors at 9 months of age for LAZ at 12 months					
LAZ	0.18 (-0.17, 0.53)	0.325	z-score	0.05 (-0.28, 0.39)	0.752
Energy intake	-0.13 (-0.29, 0.04)	0.125	10% difference^b^	-0.030 (-0.067, 0.007)	0.116
Protein intake	0.16 (0.001, 0.32)	0.048	10% difference^b^	0.025 (-0.001, 0.051)	0.055
**Model 3 –micronutrient intakes**					
Predictors at 6 months of age for LAZ at 9 months					
LAZ	0.13 (-0.22, 0.49)	0.462	z-score	0.02 (-0.33, 0.37)	0.924
Energy intake	0.07 (-0.07, 0.21)	0.330	10% difference^b^	0.014 (-0.012, 0.041)	0.290
Calcium intake	-0.15 (-0.31, 0.01)	0.075	10% difference^b^	-0.018 (-0.038, 0.003)	0.089
Iron intake	0.06 (-0.37, 0.50)	0.776	10% difference^b^	0.000 (-0.031, 0.031)	0.989
Zinc intake	-0.14 (-0.51, 0.23)	0.468	10% difference^b^	-0.006 (-0.034, 0.021)	0.644
Retinol intake	-0.04 (-0.33, 0.26)	0.813	10% difference^b^	-0.001 (-0.015, 0.013)	0.922
Riboflavin intake	0.23 (0.00, 0.45)	0.047	10% difference^b^	0.013 (-0.002, 0.027)	0.098
Vitamin A supplementation in last 6 months	-0.20 (-0.57, 0.17)	0.294	-	-0.23 (-0.59, 0.13)	0.216
Predictors at 9 months of age for LAZ at 12 months					
LAZ	0.13 (-0.22, 0.49)	0.462	z-score	0.02 (-0.33, 0.37)	0.924
Energy intake	-0.07 (-0.21, 0.06)	0.303	10% difference^b^	-0.018 (-0.048, 0.012)	0.237
Calcium intake	0.00 (-0.13, 0.13)	0.999	10% difference^b^	0.001 (-0.016, 0.018)	0.900
Iron intake	0.01 (-0.18, 0.18)	0.998	10% difference^b^	0.000 (-0.021, 0.021)	0.997
Zinc intake	0.08 (-0.08, 0.25)	0.328	10% difference^b^	0.008 (-0.011, 0.027)	0.425
Retinol intake	0.02 (-0.07, 0.10)	0.693	10% difference^b^	0.001 (-0.002, 0.004)	0.672
Riboflavin intake	0.04 (-0.09, 0.16)	0.562	10% difference^b^	0.004 (-0.007, 0.015)	0.437
Vitamin A supplementation between 6 & 9 months	-0.13 (-0.39, 0.13)	0.317	-	-0.13 (-0.37, 0.11)	0.292

LAZ: Length-for-age z-scores.

Goodness of fit statistics for all standardized models: CFI = 1.00; TLI = 1.00; RMSEA = 0.000; Model 1: χ^2^ (DF) = 6.18 (5); Model 2: χ^2^ (DF) = 6.05 (5); Model 3: χ^2^ (DF) = 17.79 (15).

^**a**^Calculated using maximum likelihood longitudinal structural equation modeling with lagged predictors. All variables were standardized except for vitamin A supplementation in the standardized models.

^b^ Predictor variables were log-transformed, so regression coefficients are presented as the mean difference for a 10% difference in the predictor.

**Table 6:** Micronutrient and systemic inflammation biomarker predictors of length-for-age z-score (LAZ) at 6 and 9 months and 9 and 12 months of age (n = 225).

	Mean difference in LAZ (95% CI)^a^ by unstandardized predictors	p-value	Units for unstandardized predictors	Mean difference in LAZ (95% CI)^a^ by unstandardized predictors, z-score	p-value
Predictors at 6 months of age for LAZ at 9 months					
LAZ	0.19 (-0.16, 0.54)	0.288	z-score	0.07 (-0.27, 0.41)	0.688
Serum ferritin	-0.13 (-0.28, 0.02)	0.100	10% difference^a^	-0.015 (-0.031, 0.000)	0.055
Serum zinc	0.09 (-0.04, 0.22)	0.197	μmol/L	0.02 (-0.02, 0.06)	0.273
Serum RBP	0.06 (-0.07, 0.20)	0.355	μmol/L	0.19 (-0.38, 0.75)	0.520
Serum selenium	-0.08 (-0.21, 0.05)	0.228	μmol/L	-0.58 (-1.55, 0.40)	0.246
Elevated AGP compared to not elevated	0.16 (-0.15, 0.47)	0.310	-	0.15 (-0.13, 0.44)	0.298
CRP	0.00 (-0.12, 0.13)	0.969	10% difference^a^	0.000 (-0.006, 0.006)	0.936
Predictors at 9 months of age for LAZ at 12 months					
LAZ	0.19 (-0.16, 0.54)	0.288	z-score	0.07 (-0.27, 0.41)	0.688
Serum ferritin	-0.05 (-0.22, 0.12)	0.575	10% difference^a^	-0.009 (-0.029, 0.010)	0.347
Serum zinc	0.02 (-0.10, 0.15)	0.694	μmol/L	0.02 (-0.06, 0.09)	0.623
Serum RBP	0.11 (-0.02, 0.24)	0.097	μmol/L	0.37 (-0.15, 0.89)	0.167
Serum selenium	0.04 (-0.08, 0.15)	0.545	μmol/L	0.12 (-0.69, 0.93)	0.772
Elevated AGP compared to not elevated	0.26 (-0.04, 0.55)	0.088	-	0.28 (0.00, 0.55)	0.050
CRP	-0.03 (-0.16, 0.09)	0.594	10% difference^a^	-0.002 (-0.009, 0.004)	0.479

LAZ: Length-for-age z-scores; RBP: Retinol binding protein; CRP: C-reactive protein; AGP: α-1-acid-glycoprotein.

Goodness of fit statistics for the standardized model: CFI = 1.00; TLI = 1.00; RMSEA = 0.000; χ^2^ (DF) = 15.78 (13).

All variables were standardized for the standardized model.

^a^Predictor variables were log-transformed, so regression coefficients are presented as the mean difference for a 10% difference in the predictor.

Notes:

For serum zinc: Adjusted for time of the day and interval since the last meal = exp (unadjusted in biomarkers + (regression coefficient for time of day x (time of day)–(regression coefficient for interval since previous meal x interval since previous meal))).

For serum ferritin, retinol binding protein, zinc, selenium: Adjusted for inflammation = exp (unadjusted in biomarkers–(regression coefficient for CRP) x (CRP − (maximum of lowest decile for CRP)) − (regression coefficient for AGP) x (AGP–(maximum of lowest decile for AGP))).

Of the demographic, nutrient, biomarker, and health predictor variables included in the full model at 6 months of age (Table 7), greater maternal height (0.18 (95% CI 0.03, 0.32)) was most strongly associated with greater infant LAZ at 9 months. At age 9 months, the strongest positive predictors of LAZ at 12 months were greater maternal height (0.16 (95% CI 0.02, 0.31)), infant being female (0.22 (95% CI -0.02, 0.45), greater protein intake (0.17 (95% CI -0.01, 0.35), higher serum RBP (0.12 (95% CI -0.01, 0.25)) and elevated AGP compared to not elevated (0.26 (95% CI -0.06, 0.58) whereas having an improved drinking water source was negatively associated with LAZ (-0.40 (95% CI -0.65, -0.14)).

**Table 7:** Demographic, nutrient, biomarker, and health predictors of length-for-age z-score (LAZ) at 6 and 9 months and 9 and 12 months of age (n = 225).

	Mean difference in LAZ (95% CI)^a^ by standardized predictors	p-value	Units for unstandardized predictors	Mean difference in LAZ (95% CI)^a^ by unstandardized predictors, z-score	p-value
Predictors at 6 months of age for LAZ at 9 months					
LAZ	0.34 (-0.07, 0.76)	0.106	z-score	0.24 (-0.16, 0,64)	0.245
Socio-economic status^b^	0.00 (-0.12, 0.13)	0.977	-Standardized score^b^	0.00 (-0.06, 0.07)	0.938
Improved drinking water source^c^	-0.15 (-0.47, 0.17)	0.354	-	-0.02 (-0.51, 0.11)	0.214
Improved sanitation facility^d^	-0.09 (-0.44, 0.26)	0.630	-	-0.06 (-0.42, 0.29)	0.727
Hand washing^e^	-0.03 (-0.12, 0.07)	0.612	-Score^e^	-0.02 (-0.10, 0.06)	0.638
Maternal height	0.18 (0.03, 0.32)	0.016	cm	0.04 (0.01, 0.06)	0.008
Maternal education (at least high school compared to less than high school)	-0.17 (-0.40, 0.06)	0.146	-	-0.16 (-0.39, 0.08)	0.188
Sex of infant (female compared to male)	0.07 (-0.21, 0.35)	0.603	-	0.10 (-0.17, 0.38)	0.466
Energy intake	-0.06 (-0.29, 0.18)	0.628	10% difference^f^	-0.006 (-0.049, 0.038)	0.801
Protein intake	0.10 (-0.15, 0.35)	0.444	10% difference^f^	0.011 (-0.031, 0.054)	0.600
Serum ferritin	-0.15 (-0.33, 0.03)	0.111	10% difference^f^	-0.017 (-0.036, 0.001)	0.066
Serum zinc	0.10 (-0.02, 0.23)	0.113	μmol/L	0.03 (-0.01, 0.07)	0.153
Serum RBP	0.11 (-0.03, 0.24)	0.129	μmol/L	0.38 (-0.19, 0.94)	0.193
Serum selenium	-0.05 (-0.20, 0.09)	0.456	μmol/L	-0.39 (-1.45, 0.67)	0.472
Vitamin A supplementation in last 6 months	-0.08 (-0.41, 0.25)	0.628	-	-0.10 (-0.40, 0.21)	0.536
Elevated AGP compared to not elevated	0.14 (-0.19, 0.47)	0.413	-	0.13 (-0.17, 0.44)	0.387
CRP	0.02 (-0.11, 0.14)	0.773	10% difference	0.001 (-0.005, 0.007)	0.724
Predictors at 9 months of age for LAZ at 12 months					
LAZ	0.34 (-0.07, 0.76)	0.106	z-score	0.24 (-0.16, 0.64)	0.245
Socio-economic status^b^	-0.05 (-0.20, 0.09)	0.463	-Standardized score^b^	-0.02 (-0.10, 0.05)	0.569
Improved drinking water source^c^	-0.40 (-0.65, -0.14)	0.003	-	-0.43 (-0.70, -0.16)	0.002
Improved sanitation facility^d^	-0.05 (-0.38, 0.28)	0.769	-	-0.05 (-0.41, 0.31)	0.772
Hand washing	-0.06 (-0.19, 0.07)	0.345	Score^e^	-0.02 (-0.07, 0.02)	0.345
Maternal height	0.16 (0.02, 0.31)	0.031	cm	0.04 (0.01, 0.07)	0.011
Maternal education (at least high school compared to less than high school)	0.00 (-0.25, 0.25)	0.994	-	-0.02 (-0.28, 0.24)	0.867
Sex of infant (female compared to male)	0.22 (-0.02, 0.45)	0.073	-	0.25 (0.00, 0.49)	0.049
Energy intake	-0.13 (-0.30, 0.04)	0.141	10% difference^f^	-0.031 (-0.070, 0.007)	0.107
Protein intake	0.17 (-0.01, 0.35)	0.058	10% difference^f^	0.029 (0.001, 0.058)	0.046
Serum ferritin	-0.05 (-0.25, 0.15)	0.601	10% difference^f^	-0.010 (-0.032, 0.013)	0.406
Serum zinc	0.02 (-0.11, 0.15)	0.769	μmol/L	0.02 (-0.06, 0.09)	0.685
Serum RBP	0.12 (-0.01, 0.25)	0.066	μmol/L	0.43 (-0.08, 0.95)	0.101
Serum selenium	0.07 (-0.07, 0.22)	0.336	μmol/L	0.36 (-0.64, 1.37)	0.479
Vitamin A supplementation between 6 & 9 months	-0.16 (-0.42, 0.11)	0.257	-	-0.16 (-0.40, 0.09)	0.214
Elevated AGP compared to not elevated	0.26 (-0.06, 0.58)	0.112	-	0.26 (-0.03, 0.56)	0.081
CRP	0.02 (-0.11, 0.16)	0.744	10% difference^f^	0.001 (-0.006, 0.008)	0.822

Goodness of fit statistics for the standardized model: CFI = 1.00; TLI = 1.00; RMSEA = 0.000; χ^2^ (DF) = 102.25 (33).

LAZ: Length-for-age z-scores; RBP: retinol binding protein; CRP: C-reactive protein; AGP: α-1-acid-glycoprotein.

^a^Calculated using maximum likelihood longitudinal structural equation modeling with lagged predictors. All continuous variables were standardized (maternal height, all dietary intake variables and biomarkers) for the standardized model. Socio-economic status, improved drinking water source, improved sanitation facility, maternal height, maternal education, and sex of infant were included as time-invariant predictors.

^b^Measured by wealth index.

^c^Includes water sources such as piped water, tube well/borehole, protected dug well, protected spring.

^d^Includes flush or pour-flush to piped sewer system, septic tank or pit latrine, ventilated improved pit latrine, pit latrine with slab and composting toilet, unshared with other households.

^e^Measured at 6, 9, and 12 months. Hand washing measured as a score for the number of scenarios that mothers washed their hands.

^f^ Predictor variables were log-transformed, so regression coefficients are presented as the mean difference for a 10% difference in the predictor.

Notes:

For serum zinc: Adjusted for time of the day and interval since the last meal = exp (unadjusted in biomarkers + (regression coefficient for time of day x (time of day)–(regression coefficient for interval since previous meal x interval since previous meal))).

For serum ferritin, retinol binding protein, zinc, selenium: Adjusted for inflammation = exp (unadjusted in biomarkers–(regression coefficient for CRP) x (CRP − (maximum of lowest decile for CRP)) − (regression coefficient for AGP) x (AGP–(maximum of lowest decile for AGP))).

### Sources of drinking water and contamination during household follow-up

Of the 230 participants, 175 (78%) households provided drinking water samples when their infants were ~ 60–66 months of age. No significant differences in household SES, wealth index, drinking water source, sanitation facility, maternal education, were found between households providing drinking water samples and those households who did not (n = 50), with the exception of maternal height (149.8 vs. 152.0 cm, p = 0.007).

Among the 175 households, 145/175 (83.3%) had a source of drinking water categorized as improved based on the Indonesia DHS 2017 [37]. Of these, the most frequent type of improved sources was protected spring. However, of these improved sources, 90.3% (131/145) had drinking water at point of collection and 45.7% (53/116) had drinking water at point of use (i.e., ready to drink) with evidence of fecal contamination (*E*. *coli*).

## Discussion

To our knowledge this is the first study to use dynamic panel data modelling in Indonesia to examine longitudinal relationships among nutrient intakes, WASH, micronutrient and inflammation biomarkers, and socio-demographic variables with the potential to influence LAZ among resource-poor Indonesian infants. Of all the variables examined, household drinking water categorized as “improved” according to the WHO and UNICEF [15] metric was the strongest predictor of infant LAZ at 12 months of age although surprisingly, this association was negative. However, in our household follow-up investigation, fecal contamination at the point of collection was found in almost all households categorized as having an “improved” water source, which persisted in nearly 50% of the households after boiling or giving other treatments to the drinking water at the point of use. Clearly, in the Sumedang district, ensuring water safety at the point of consumption is essential.

Exposure to fecal contamination during infancy and early childhood has been strongly linked to environmental enteric dysfunction (EED) [38], an asymptomatic syndrome potentially underlying poor growth [39]. EED is characterized by chronic intestinal inflammation, reduced nutrient absorption, and increased intestinal permeability [39], all of which can result in adverse effects on linear growth [40]. Among young children in the Gambia, for example, over 40% of growth faltering was explained by increased intestinal permeability, a measure of EED, whereas diarrhea was not associated with growth failure [31]. The high prevalence of elevated levels of fecal MPO among the infants tested here at age 12 months is indicative of increased intestinal neutrophil activity associated with gut inflammation, likely resulting from frequent enteric infections. Intestinal inflammation may exist for extended periods of time even in the absence of overt clinical symptoms of diarrhea [41], which among these breastfed Sumedang infants at both 9 and 12 months of age was relatively low (i.e., < 12%) [13]. Hence, it is conceivable that intestinal inflammation, possibly together with the other adverse metabolic effects such as increased intestinal permeability and reduced nutrient absorption that characterize EED, had a role in the decline in the LAZ of these resource-poor infants. Interestingly, in a recent cohort of Pakistani children at high risk of EED [42], a negative relationship between MPO, a biomarker of gut inflammation, and IGF-1 growth hormone was reported, which was in turn strongly associated with linear growth. Certainly, JH Humphrey [43] argues that EED and not diarrhea may be the primary causal mechanism linking WASH to child growth.

In contrast with earlier reports [44–46] that showed inflammatory proteins such as CRP and AGP were inversely associated with IGF-1 and linear growth, in our study a non-significant but positive relationship was observed between elevated AGP at 9 months and LAZ at 12 months. This unexpected finding may be linked, at least in part, with catch-up growth that may have occurred once growth-inhibiting conditions such as inflammation/infection, were resolved [47]. It is conceivable that the positive effect of continued breastfeeding up to 12 months of age on immunity against severe gastrointestinal infections [48] together with improved gut integrity associated with higher RBP concentrations [49,50] may have facilitated the recovery process.

Only three other variables at 9 months of age (maternal height, protein intake, and serum RBP) were significant and positive predictors of infant LAZ at 12 months of age. Of these, the positive and unmodifiable relationship observed here between maternal height and infant LAZ is well documented in Indonesia [9] and elsewhere in Asia [51]. Inter-generational poverty is likely to have had some negative impact on the genetic growth potential of the Sumedang mothers and their offspring [52]; 16% had a height < 145 cm, considered a proxy for generational influences, and the infants of all these mothers had LAZ below zero by 12 months of age. In a study based on the Demographic and Health Survey data from 54 low and middle-income countries [17], every 1 cm greater maternal height was associated with a decreased risk in stunting [relative risk (RR), 0.968, (95% CI 0.967, 0.968)].

Of the five micronutrient biomarkers investigated here, only serum RBP at 9 months of age was significantly associated with infant LAZ. We used serum RBP as a surrogate measure for serum retinol because it is more stable and easier to analyze [53]. Vitamin A is known to have both promoting and regulatory roles in the innate immune system and adaptive immunity [54]. Children with lower serum retinol, and presumably poorer vitamin A nutritional status, are more likely to have impaired intestinal integrity [55]; and vitamin A supplementation has shown improvements in intestinal permeability [49,50]. Consequently, the role of vitamin A in enhancing the immune function of the infants and providing an enhanced defense against infections probably accounted, at least in part, for the positive relationship observed here between RBP at 9 months and LAZ at 12 months of age. Interestingly, in several earlier vitamin A supplementation trials of preschool children in Indonesia, positive effects on linear growth have been reported, although the magnitude of the effect appear to be dependent on season, vitamin A intake, and morbidity [56]. Here, season was unlikely to be a factor influencing infant linear growth because rainfall occurred during most months in the study area. Moreover, infant recruitment covered the so-called dry (March to September) and wet (October to February) seasons. Clearly efforts should be made to strengthen the vitamin A supplementation program in combination with increasing intakes of vitamin A-rich and vitamin A-fortified foods for infants during early post-partum in this setting to improve serum RBP concentrations [57].

Of the dietary variables examined (after adjusting for potential confounding), including our nutrient quality score developed previously [13], only protein intake at 9 months had a significant and positive influence on LAZ at age 12 months. This finding suggests that protein intake is a better predictor of growth than other nutrient variables investigated [13]. Furthermore, although the intake of protein exceeded the estimated needs at 9 months of age, there were shortfalls in the energy and selected micronutrient intakes from complementary foods when compared to the WHO estimated needs, some of which were probably additional growth-limiting factors [18].

Unlike several other reports in Indonesia [9,58] and elsewhere [51], we observed limited evidence of the influence of SES, maternal education, improved sanitation facility, or handwashing on the LAZ of these resource-poor infants at age 12 months. Reasons for these inconsistent findings are uncertain but may have been associated with differences in the samples used and populations they represent. For example, in this study there was limited variation in these demographic measures, as shown in Table 1: almost all the mothers were housewives (91.6%) with husbands who were manual laborers or farmers without a regular income; very few (~ 6%) had attended a tertiary education institution, and almost all (85%) households had an improved sanitation facility.

In contrast, sex (i.e., being female) was a strong and positive predictor of LAZ at 12 months of age, consistent with the findings of others in Indonesia [58,59] and elsewhere [60]. Here, female infants had higher energy intakes than males at 9 months despite a lower energy requirement [36]. However, adjusting for energy intake only served to strengthen the association between sex and LAZ at 12 months (Table 7). Whether such sex differences are linked with biological differences that are independent of infant feeding patterns remains unclear [56].

### Strengths and limitations

As noted above, our comprehensive data from our earlier cohort study allowed us to study multiple predictors, which when combined, have the potential to impact on the linear growth of these resource-poor Indonesian infants [11,18]. Moreover, the longitudinal nature of our infant data collection allowed causal sequencing to be explored. Our follow-up collection and analyses of household drinking water samples at the point of consumption provided some evidence that exposure to fecal contamination likely contributed to the unexpected negative relationship observed between “improved” water source and LAZ at 12 months.

Nevertheless, our sampling of the drinking water was cross-sectional and performed 54–60 months after the collection of the infant data at age 12 months, and hence did not provide information on temporal variability in water quality which is known to depend on season and/or sporadic contamination [61]. Moreover, our infants at age 12 months were likely to be mobile and continuously exposed around their home compounds to pervasive poultry fecal contamination via multiple and interrelated fecal-oral pathways [62]. Consequently, it is not possible to ascribe the effect of fecal contamination in drinking water as the only source of transmission of fecal pathogens in this study setting. Nevertheless, we suggest that irrespective of the source, transmission of fecal pathogens through the fecal-oral route probably placed these resource-poor rural infants at high risk for EED during later infancy, a syndrome inversely correlated with linear growth in early postnatal life [40]. However, because only one marker of EED (MPO) was measured here in a subset of the infants at 12 months of age because of refusal or insufficient volume of stool samples, we were unable to confirm this hypothesis.

For these breastfeeding infants, data on both nutrient intakes *per se* and our nutrient quality score were based on the contribution from complementary foods alone because we measured neither the intake or composition of human milk here. Moreover, the measured complementary food intakes were over a short time interval (i.e., two days), although adjusted to represent “usual” intakes using the Multiple Source Method (MSM) [19]. Our nutrient quality score used standardized micronutrient intakes from weighed food records in the calculation of the scores, so that infants who consumed lower amounts had a lower score. Finally, while our models convey potential causal information from longitudinal data, this study is observational and should be interpreted as such. In addition, because our households were randomly selected only from Sumedang district, our findings may not apply to infants in other poor resource settings in Indonesia. However, the proportion of stunting in our rural Sumedang infants at 9 months was comparable to that reported for 6–11 month-old infants in the West Java Province (19.3 vs. 18.6%) [63], the most populous province in Indonesia; and also comparable to the national level (21.5%) [8]. Several characteristics of the Sumedang households were also comparable to those reported at the national level in the most recent Demographic and Health Survey [37], including the presence of both a sanitation facility (81.3 vs. 80.0%) and drinking water source (62.7 vs. 75.0%) categorized as improved, and the level of primary education or less for the parents in the households (i.e., 43.7 vs. 46.0%). Finally, we are not the first to report that access to an “improved water source” as defined by the WHO and UNICEF [15] metric does not necessarily ensure drinking water free of fecal contamination [61,64]. Hence the negative influence of improved water source on LAZ is not surprising. In light of these findings, WHO/UNICEF revised their operational definitions for improved drinking water supply, treated water, and improved sanitation facilities [65]. We could not apply all the WHO revised criteria in our dataset (Table 7) because we did not perform water examination for fecal contamination when we collected the original data in 2014–2015. However, instead, we selected the revised WHO criteria related to WASH only (with the exception of chemical contamination) based on our household follow-up data collected 54–60 months after the infant data at 12 months of age. Interestingly, applying these revised WHO criteria, only 7/145 (5%) of the households studied at 54–60 months after the collection of the infant data at 12 months of age would have had drinking water classified as safely managed. These findings emphasize that when interpreting studies based on household drinking water, attention must be given to the metric that has been applied.

### Conclusions and recommendations

In this rural Indonesian setting, two variables (“improved” water source and maternal height) at age 9 months were significant predictors of the LAZ at 12 months for these resource-poor infants. The unexpected negative influence of “improved” water source on LAZ was the strongest association likely linked in part to fecal contamination of the drinking water which persisted even after boiling in some households. Hence, our findings emphasize that “improved” water sources are not universally, nor consistently free of fecal contamination and that applying the source-type classification alone in surveys may be misleading. Instead, the improved water source metric should be adopted and consideration given to testing the drinking water source at the point of consumption to ensure the supply is safe. In addition, health promotion in this setting should also highlight the importance of a safe water supply.

Based on our findings, we recommend strengthening the national nutrition education program to improve maternal nutritional status during pregnancy and lactation with the aim of enhancing child growth and breaking the intergenerational cycle. Messages to promote exclusive breastfeeding up to the first six months and to boil drinking water appropriately to ensure water is always safe for consumption should also be included. Finally, in the future, population-based measures of intestinal injury and altered intestinal function should be included in studies designed to address the predictors of linear growth during infancy and childhood in all low resource settings.

## Supporting information

S1 Dataset Infant and young child characteristics of infants in Sumedang district, Indonesia. (XLS)

S1 File Questionnaires PLoS ONE. (PDF)
