# Supplementary material for: Determination of modifiable risk factors for length-for-age z-scores among resource-poor Indonesian infants
Source: PLoS One. 2021 Feb 18;16(2):e0247247. doi: 10.1371/journal.pone.0247247 (PMC7891771; doi:10.1371/journal.pone.0247247)
Supplement: S1 File — (PDF) [file pone.0247247.s002.pdf]

QUESTIONNAIRES 6 MONTH

Adequacy of Micronutrient Intakes and Status of

Breastfed Indonesian Infants

Fed Traditional Complementary Foods

Sumedang District

2014 - 2015

PART 0 - ELIGIBILITY CHECKLIST FOR INTERVIEWER

1) *Interviewer:**
interviewer_elig
Interviewer is a field doctor who has been standardised.
Use your initial every time you have to fill in the interviewer question.
1. AD : Aly Diana
2. EI : Eva Indirawati
3. M : Monik
4. DEL : Dimas Erlangga Luftimas
5. R : Rima
6. H : Hendro
66. Others

2) *Date of interview (dd/mm/yyyy):**
date2_elig
Use format day/month/year to fill in this interviewer question.
Every time you have to fill in the date, please use dd/mm/yyyy format.

3) *Mother's name:**
mom_elig

*It is better to fill in the full name of mother. If it is not possible, you can fill in the forename, middle name or nick name. Please match the name that was told by mother with the name written in the paper**.*

4) *Mother's ID number:**
mom id_elig
*It is no need of asking this question. It is written in the paper** given to you. Please re-check once again whether you have typed the ID number correctly.*

5) *Phone number*
hp_elig
Fill mother's phone number

6) *Mother's date of birth (dd/mm/yyyy):**
mom birth_elig
Please ask the mother about her birth date. If she cannot remember or look unsure about her answer, please ask for her ID card, and then write down the date.

7) *Infant's name:**
baby_name_elig
*It is better to fill in the full name of infant. If it is not possible, you can fill in the forename, middle name or nick name. Please match the name that was told by mother with the name written in the paper**.*

8) ***Father's name:****
dad_elig
It is better to fill in the full name of father.

9) ***Cadre's name***
cadre_elig
1. Syarifah
2. Rosmiati
3. Entin
4. Saadiah
5. Enok
6. Edja
7. Siti
8. Nunung
9. Ratna
10. MIntarsih
11. Dania
66. Others
99. Missing

10) ***Infant's date of birth (dd/mm/yyyy):****
baby_elig
Please use the format day/month/ year to fill in this question, please check with the data in the paper given (if available), as well as in the KMS (health card). If the date is different; write down the date mentioned in the KMS and please make a note in the paper.

11) ***Age of infant is 6 month (+/- 2 weeks):***
baby_age_elig
In this question the important thing is whether the age of infant is 6 month +/- 2 weeks or not. Please re-check using the birth date mentioned in the KMS.
1. Yes
0. No

12) ***Length of pregnancy >37 weeks:***
pregnancy_elig
The important thing in this question is whether the length of pregnancy >37 weeks (at term) or not. Please check in the pink book (Buku KIA), and answer the question following the information from pink book. If the pink book is not available, please ask the mother and answer the question following the mother's memory.
1. Yes
0. No

13) ***Birth weight >1500 gram:***
bw_elig
In this question the important thing is whether birth weight >1500 gram or not. Please check in the pink book (Buku KIA)/health card (KMS), and answer the

question following the information from pink book. If the pink book/health card is not available, please ask the mother and answer the question following the mother's memory.

1.Yes

0. No

14) *Exclusive/predominant breastfeeding at least for 4 months:*

bf_elig

*Please ask the mother whether the infant gotexclusive/predominant breastfeeding at least for 4 months. Definition of **exclusive breastfeeding**: the infant receive breastmilk (including milk expressed or from wet nurse) and ORS, drops, syrups (vitamins, minerals medicines); and **NOTHING** else. Definition of **predominant breastfeeding**: the infant receive breastmilk (including milk expressed or from wet nurse) as the predominant source of nourishment and certain liquid (water and water-based drinks, fruit juice), ritual fluids and ORS, drops or syrups (vitamins, minerals medicines); and **NOTHING** else. Giving the infant any form of liquid (honey, glucose, etc.) during the first day of life is still acceptable as a form of **ritual fluids**.*

1. Yes

0. No

15) *Mother agrees to writte informed consent:*

consent_elig

It is important for mother to know about what will researcher do to her child. Please give a comprehensive information about our research and all the steps. Ask the mother to sign the informed consent if she agreed. If in case the parents have already signed the informed consent before hand, please just remind the mothers about the steps of our research, read it from the information page – and then answer the following questions number 13-15.

1. Yes

0. No

16) *Mother agress to allow home visit for dietary weighed record and home observation:*

visit_elig

In this question the important thing is whether mother agreesor not to allow home visit for dietary weighed record and home observation.

1. Yes

0. No

17) *Mother agrees to answer questions related to demographics, socio-economics, pregnancy, delivery, morbidity, health, caring practice, food, and development of infant:*

question_elig

Please ask the mother whether agrees or not to answer questions related to demographics, socio-economics, pregnancy, delivery, morbidity, health, caring practice, food, and development of infant.

18) *Mother agrees to let her infant undergo specified anthropometric measurements (weight, length, head circumference, mid-upper arm circumference), blood, urine, and stool collection:*

exam_elig

Please ask the mother whether mother agrees to let her infant undergo specified anthropometric measurements (weight, length, head circumference, mid-upper arm circumference), blood, urine, and stool collection. Anthropometric measurements (weight, length, head circumference, mid-upper arm circumference), urine, and stool collection will be done by the field doctor during the home visit. Please inform the mother that blood collection will be done by PRODIA laboratory staff, every Thursday.

1. Yes
0. No

If the answer to any of above questions is NO, then infant cannot be enrolled in the study.

19) At the time of enrolment, does the infant have evidence of severe disease (active tuberculosis, severe anemia [haemoglobin<7 g/dL], acute malnutrition [mid-upper arm circumference <11.5 cm]):

ill_elig

Please ask the mother whether her infant have evidence of severe disease or not. Active tuberculosis means the infant has been diagnosed active tuberculosis by the health worker. You can examine the conjungtiva of infant to see haemoglobin level at a glance. To detect acute malnutrition case you should examine mid-upper arm circumference. If the mid-upper arm circumference <11.5 cm; the infant should be referred to health worker.

If the infant hasn't been diagnosed of TB or anemic *and you find any signs and symptoms of TB as well as anemia or any other severe diseases, please refer the infant to the nearest health worker. In this case, please don't give any answer Yes/No; wait for the result from the health worker. Don't forget to do a follow-up.*

If the answer to any of above questions is YES, then infant cannot be enrolled in the study. However, no matter whether the infant can be enrolled in the study or not, please continue with these demographic questions (number 18-23).

20) Mother's (most recent) occupation:

mom_job_elig

You have to ask the most recent occupation of mother and fill in this question. If the mother said that she does not work, please choose housewife.

Options:

1. Civil servant
2. Private employee (office work
3. Entrepreneurship
4. Agricultural worker
5. Blue collar
6. Housewife
7. Teacher

66. Other
98. Don't know
99. Missing

21) **What is the highest level of education mother has received?**
mom_edu_elig
You have to ask the mother about her last education that she was graduated from. For example if she has not graduated from elementary school, then she will be considered as never attended school.
Options:
1. Never attended school
2. Elementary School
3. Junior High School
4. Senior High School
5. Academy/Vocational training
6. University
98. Don't know

22) ***Father's (most recent) occupation:***
dad_job_elig
You have to ask the most recent and main occupation of father and fill in this question.
Options:
1. Not working
2. Civil servant
3. Private employee (office work)
4. Entrepreneurship
5. Agricultural worker
6. Blue collar
7. Teacher
8. Farmer
66. Other
98. Don't know
99. Missing

23) **What is the highest level of education father has received?**
dad_edu_elig
You have to ask the father about her last education that he was graduated from. For example if he has not graduated from elementary school, then he will be considered as never attended school.
Options:
1. Never attended school
2. Elementary School
3. Junior High School
4. Senior High School
5. Academy/Vocational training
6. University
66. Other
98. Don't know
99. Missing

Please remember - if the answer to question 19 is yes, then infant cannot be enrolled in the study.

24) *CONCLUSION*

conclusions_elig

Please select "enrolled" if respondents fulfill all the eligibility, select "Ineligible" if not. Please select "refused", if she doesn't want to join the research.

Options:

1. Enrolled
2. Ineligible
3. Refused

25) If refused, please specify:

refuse_elig

If mother refused to join the research please specify the reason clearly.

*Required questions, you have to fill in these questions to be able to continue

**The paper consists of name of mother, mother's ID, and infant's ID – you have to bring the paper with you during the home visit.

PART 1 - IDENTIFICATION OF RESPONDENTS

1) ***Interviewer:****
   interviewer_fam_6m
   Interviewer is a field doctor who has been standardised.
   Use your initial every time you have to fill in the interviewer question.
   1. AD : Aly Diana
   2. EI : Eva Indirawati
   3. M : Monik
   4. DEL : Dimas Erlangga Luftimas
   5. R : Rima
   6. H : Hendro
   66. Other

2) ***Date of interview (dd/mm/yyyy):****
   date2_fam_6m
   Use format day/month/year to fill in this interviewer question.

3) ***Mother's name:****
   name_fam_6m
   It is better to fill in the full name of mother. If it is not possible, you can fill in the forename, middle name or nick name. Please match the name that was told by mother with the name written in the paper.

4) ***Mother's ID number:****
   mom id_fam_6m
   *It is no need of asking this question. It is written in the paper** given to you. Please re-check once again whether you have typed the ID number correctly.*

5) **Has the interviewer asked the mother to record the conversation that will be done? Has the interviewer prepared the recorder?**
   record_fam_6m

   Please remember that the conversations or the questioning process must be recorded as a back-up data, in case the off-line survey (SurveyGizmo) encountered any unwanted problem. The only answer of these questions is 1. Please make sure that the recorder functioned optimally during each visit.
   Options:
   1. Yes
   2. Yes, but mother doesn't allow

6) **Infant's sex:***
   sex_fam_6m
   Ask the mother about infant's sex.
   Options:
   1. Male
   2. Female

7) Infant's birth order:*

order_fam_6m

Ask the mother about Infant's birth order. Count all the live birth and still birth, but exlude aborted baby(ies). Classify the birth order into'first infant', 'second infant', or 'third or subsequent'.

Options:

1. First infant
2. Second infant
3. Third or subsequent

8) Number of pregnancies:

preg_fam_6m

This question asks about number of pregnancy(ies) that the mother have ever had. All cases of aborted, live birth, still birth werecounted.

Options:

1. 1
2. 2
3. 3
4. 4
5. 5+

9) Number of deliveries (live birth):

birth_fam_6m

Ask the mother about number oflive birth.

1. 1
2. 2
3. 3
4. 4
5. 5+

10) Please specify the age of your children (in years):

Ask the mother about the age of her children (in years) when they have their last birthday.

First child

child_1_fam_6m

1. 1
2. 2
3. 3
4. 4
5. 5+

97. NA

Second child

child_2_fam_6m

1. 1
2. 2
3. 3
4. 4
5. 5+

97. NA

Third child
child_3_fam_6m
1. 1
2. 2
3. 3
4. 4
5. 5+
97. NA

Fourth child
child_4_fam_6m
1. 1
2. 2
3. 3
4. 4
5. 5+
97. NA

Fifth child
child_5_fam_6m
1. 1
2. 2
3. 3
4. 4
5. 5+
97. NA

11 Village name:
village_fam_6m

Please select the village which is respondent's house located. Please also check with the paper that you have.

1. Cigendel
2. Cijeruk
3. Pamulihan
4. Haurngombong
5. Cilembu
6. Mekarbakti
7. Cimarias
8. Cinanggerang
9. Sukawangi
10. Ciptasari
11. Citali
12. Sukasari
13. Genteng
14. Banyuresmi
15. Nanggerang
16. Mekarsari
17. Sindangsari

18. Sukarapih
19. Gudang
20. Tanjungsari
21. Jatisari
22. Margaluyu
23. Kutamandiri
24. Margajaya
25. Raharja
26. Cijambu
27. Pasigaran
28. Gunungmanik
29. Kadakajaya
30. Cinanjung
99. Missing

12) Address:

address_fam_6m

Write the full address. If the mother doesn't know her full address; it's is ok to write the address as full as she knows.

13) Marital status:

marriage_fam_6m

In this question the important thing is the marital status of mother. Still married, widowed (because the husband is dead), divorced or separated (by law), single or other.

1. Married
2. Widowed
3. Divorced or separated
4 . Single
66. Other

These are questions about the HOUSEHOLD

14) Total number of adults (over 18 years) in the household:

adult_fam_6m

Calculate the total number of adults (over 18 years) in the household to fill in this question.

1. 1
2. 2
3. 3
4. 4
5. 5
6. 6
7. 7
8. 8
9. 9
10. 10+
99. Missing

15) Number of children < 5 years in the household:
toddler_fam_6m

Calculate the total number of children < 5 years in the household to fill in this question.

1. 1
2. 2
3. 3
4. 4
5. 5
6. 6
7. 7
8. 8
9. 9
10. 10+

99. Missing

16) Number of children 6-12 years in the household:
schoolage_fam_6m

Calculate the total number of children 6-12 years in the household to fill in this question.

1. 1
2. 2
3. 3
4. 4
5. 5
6. 6
7. 7
8. 8
9. 9
10. 10+

99. Missing

17) Number of children 13-17 years in the household:
adol_fam_6m

Calculate the total number of children 13-17 years in the household to fill in this question.

1. 1
2. 2
3. 3
4. 4
5. 5
6. 6
7. 7
8. 8
9. 9
10. 10+

99. Missing

18) Total number of persons in the household:
total_fam_6m

Calculate the total number of person in the household to fill in this question. It is used as a data to analyze the proportion of number person with the size of house. The population of house related to air circulation and sanitation.

1. 1
2. 2
3. 3
4. 4
5. 5
6. 6
7. 7
8. 8
9. 9
10. 10
11. 11
12. 12
13. 13
14. 14
15. 15+
99. Missing

HOUSING and GOODS

19) Do you own your own house?
own_house_fam_6m
The important in this question is whether she owns her own house or not. It is used to see the economic level of the household.

1. Yes
0. No

20) Is this house yours?
your_house_fam_6m
The important in this question is whether that house is her own or not

1. Yes
0. No

21) Does your household have:

You have to ask the mother about the things that own by her/members of her family. Those things include some household equipment, transportation vehicle, communication facility, and electronic equipment. It is asked to measure the economic level of the household.

	Code	1. Yes	0. No	99. Missing

clock or watch	clock_fam_6m			
Television	tv_fam_6m			
telephone or mobile phone	hp_fam_6m			
Refrigerator	fridge_fam_6m			
Bicycle	bicycle_fam_6m			
Motorcycle	motor_fam_6m			
Car	car_fam_6m			
Electricity	electricity_fam_6m			
Radio	radio_fam_6m			
Maid	maid_fam_6m			

22) Does your house have a garden or agricultural land?
garden_fam_6m

The important thing in this question is whether she has a garden or agricultural land. It is being asked to assess whether any vegetables or spices or herbs that the household consumed come from the garden or not.

1. Yes
0. No

23) Is any food for the household grown in the garden or agricultural land?
eat_fam_6m

You have to ask if there is any food for the household grown in the garden/agricultural land or not.

1. Yes
0. No

24) If the answer is 1, please specify the type of food:
plant_fam_6m

You have to specify the answer from mother about any food that she got from her garden. Please write the list of all food which come from her own garden/agricultural land.

Yesterday during the day or night (for questions 25-31):
These questions are asked to check the dietary diversity among the household. Please answer 1, if ANY members of the household consume the specified food. The number of the food is not of concern. Read the food lists. Underline the

corresponding foods consumed and tick the column 1 or No depending on whether any food item of the list was consumed.

25) Did your household eat grains, roots and tubers?

	Code	1. Yes	0. No	99. Missing
Porridge, bread, rice, maize, noodles or other foods made from grains	rice_fam_6m			
White potatoes, white yams, cassava or any other foods made from roots	root_fam_6m			

26) Did your household eat any foods made from beans (kidney beans, mung beans, soy beans, tofu, tempeh), peas, lentils, nuts (peanuts) or seeds?
nuts_fam_6m

1. Yes
0. No

27) Did your household consume any dairy products?

	Code	1 Yes	0 No	99. Missing
Milk, such as tinned, powdered or fresh animal milk	milk_fam_6m			
Yogurt or drinking yogurt	yoghurt_fam_6m			
Cheese or other dairy products	cheese_fam_6m			

28) Did your household eat any flesh foods?

	Code	1 Yes	0 No	99. Missing
Liver, kidney, heart or other organ meats	organ_fam_6m			
Any meat, such as beef, pork, lamb, goat, chicken or duck	flesh_fam_6m			

Fresh or dried fish, shellfish or seafood	seafood_fam_6m			

29) Did your household eat chicken eggs, duck eggs or quail eggs?

egg_fam_6m

1. Yes
0. No

30) Did your household eat yellow/red/orange fruits and/or dark green leafy vegetables?

Pumpkin, carrots, tomatoes, squash or sweet potatoes that are yellow or orange inside	Code	1 Yes	0 No	99. Missing
Any dark green leafy vegetables, e.g. spinach, cassava leaves, water spinach, broccoli, caweightage, lettuce	yellow_fam_6m			
Ripe mangoes (fresh or dried), ripe papayas	green_veg_fam_6m			
Pumpkin, carrots, tomatoes, squash or sweet potatoes that are yellow or orange inside	orange_veg_fam_6m			

31) Did your household eat any other foods mentioned below?

Ask the mother whether household eat or not any food in classification, such as oils fat classification, sugary food classification, any sweet beverage classification, any coffee and/or tea, and any condiments classification.

	Code	1 Yes	0 No	99. Missing
Any oils, fats, or butter or foods made with any of these	oil_fam_6m			
Any surgary foods, such as chocolates, sweets, candies, pastries, cake or biscuits	sugar_fam_6m			
Any sweet beverage (soda, fruits flavoured drink)	beverage_fam_6m			
Any coffee and/or tea	coffee_fam_6m			

Condiments for flavour, such as chilies, spices, herbs, soy sauce, ketchup, or fish powder	spice_fam_6m			

32) Does this household own any farm animals, e.g. milk cows, cattle, fish, poultry?
animals_fam_6m

Ask the mother whether her household own any farm animals or not. It is being asked to assess whether any animal protein that the household consumed come from the farm or not. In addition, it will also reflect the economic condition of the household.

1. Yes
0. No

If the answer is No, then go to question number 46.

33) If 1, please specify (type and number):
animals_type_fam_6m

Ask the mother to specify the type of animals, and how many animals that she has.

34) Does your household eat meat, poultry, fish or eggs [produced in your farm]?
eat_fam_6m

You have to ask the mother whether household eat meat, poultry, fish or eggs produced in her farm.

1. Yes
0. No
97. NA

35) Number of meals per day your household usually eats (excluding snacks):
meal_fam_6m

Please specify the number of meals per day her household usually eats, excluding snacks. Household eating schedule often related to infant's eating schedule.

1. 1
2. 2
3. 3
4. 4
5. 5+
99. Missing

36) Number of snacks (made or bought) per day your household usually eats:
snack_fam_6m

Please specify the numberof snacks (made or bought) per day her household usually eats. Remember this question as only for the number of snacks.

1. 1
2. 2
3. 3
4. 4
5. 5+
99. Missing

37) Has your household received any food assistance (e.g. from the government, neighbors, relatives) in the last 6 months?

rice2_fam_6m

The important thing is whether her household received or not any food assistance in the last 6 months. It shows the economic level of household.

1. Yes
0. No

38) Where has this week's food mainly come from? (only one answer is allowed)

source_fam_6m

Ask the mother whereweek's food mainly come from.Please choose only one answer. If the mother told you several food source, encourage her to choose only one source listed in the question. If the answer does not listed, write the answer in 'other' option.

1. Garden
2. Purchased
3. Both garden and purchased
4. Wages in kind after working
5. Eating wild foods not traditionally used in daily diet
6. Food gifts from relief organisations, churches, government
7. Given by other family
8. Purchased and given by other family
9. Purchased and borrow from others
66. Other

PART 2 - Antibiotics, Morbidity, Hygiene, Sanitation

1) *Interviewer:**

interviewer_morb_6m

Interviewer is a field doctor who has been standardised.

Use your initial every time you have to fill in the interviewer question.

1. AD : Aly Diana
2. EI : Eva Indirawati
3. M : Monik
4. DEL : Dimas Erlangga Luftimas
5. R : Rima
6. H : Hendro

66. Other

2) Mother's name:*

mom_morb_6m

It is better to fill in the full name of mother. If it is not possible, you can fill in the forename, middle name or nick name. Please match the name that was told by mother with the name written in the paper.

3) Mother's ID number:*

id_morb_6m

It is no need of asking this question. It is written in the paper** given to you. Please re-check once again whether you have typed the ID number correctly.

4) Did you (mother) consume antibiotics during your last month of pregnancy?

mom_ab_morb_6m

You have to ask the mother whether mother consumesor not antibiotics during her last month of pregnancy. The data are used to identify the correlation between antibiotic consumption of mother and infant's gut microbiota composition.

1. Yes

0. No

98. Don't know

99. Missing

If No, then go to question number 8

5) If 1, please specify the name of antibiotics:

name_morb_6m

Ask the mother to show you the drug/the blister,if she is still keeping it. If not, please encourage the mother to tell the name of antibiotic she consumed.

98. Don't remember

6) How long did you consume the antibiotics?

long_ab_morb_6m

Fill this question with duration of mother's antibiotic consumption, and not with duration of prescription (told by the doctor). Please specify in days.

7) How many pills a day?

number_ab_morb_6m

Fill this question with number of antibiotic pills/tablets consumed, and not with number of pills in the prescription (told by the doctor).

1. 1
2. 2
3. 3
4. 4+

97. NA
99. Missing

DELIVERY HISTORY

8) Where and how did you deliver (NAME)?

delivery_morb_6m

This question means the place where mother delivered the NAME (NAME refers to child's name) and how she delivered.

1. Vaginal at home
2 . Vaginal at local health facilities (Puskesmas, midwife's clinic)
3. Vaginal at hospital
4. Caesarian at hospital
99. Missing

9) What is the birthweight of (NAME)?

birth_weight_morb_6m

The important thing is to classify the birthweight of NAME and fill in this question (NAME refers to child's name)

1. < 2500 gr
2. 2500 – 4500 gr
3. > 4500 gr
99. Missing

10) Has (NAME) been hospitalised soon after delivery?

hospital_morb_6m

This question means whether (NAME) has been hospitalised soon after delivery or not. If NAME has been delivered through cesarean section you can ask to mother did the infant has to get any specific medical treatment because of any disorder or not.

1. Yes
0. No
99. Missing

If No, then go to question number 12

11) If 1, please specify the reason(s) for hospitalisation:

reason_hosp_morb_6m
This question means the reason why (NAME) has been hospitalization soon after delivery.

MORBIDITY

12) Has your infant been sick in the last 2 weeks?
sick_2w_morb_6m
This question means whether**infant has been sick in the last 2 weeks or not or don't know.**
1. Yes
0. No
98. Don't know
99. Missing

If No, then go to question number **23.**

13) Has your infant been vomiting in the last 2 weeks?
vomit_2w_morb_6m

The important thing is whether the infant has been vomiting in the last 2 weeks or not. Please choose '1' or 'no'. If mother work outside house and she doesn't know whether the infant vomit or not, please choose 'don't know'.
1. Yes
0. No
98. Don't know
99. Missing

14) Has your infant been ill with a fever at any time in the last 2 weeks?
fever_2w_morb_6m
Ask the mother whether the infant has been ill or not with a fever at any time in the last 2 weeks. Underlying disease can cause any change in nutritional status.
1. Yes
0. No
98. Don't know
99. Missing

15) Has your child had diarrhea in the last 2 weeks?
diarrhea_2w_morb_6m
The purpose of this question is to know whether the infant had diarrhea in the last 2 weeks or not.
1. Yes
0. No
98. Don't know
99. Missing

If No, then go to question number 20.

16) If 1, how many sachets of Oralit (ORS) had been given to your infant during the whole episode of diarrhea?
ors_sachet_morb_6m

The important thing of this question is how many sachet oralithad been given to the infant as oral rehydration solution during the whole episode of diarrhea (total number).

1. 0
2. 1-3
3. 4-5
4. 6+
97. NA

17) How many glass (ml) of ORS had been given to your infant every time he/she defecate?

ors_glass_morb_6m

The purpose of this question is to know the number of glass (ml) of ORS had been given to the infant every time he/she defecate.

1. 0
2. <50 ml
3. 50 ml (1/4 glass)
4. 100 ml (1/2 glass)
5. 150 ml (3/4 glass)
6. 200 ml (1 glass)
7. >200 ml
97. NA
98. Don't know

18) How many days was your infant given zinc supplements?

zinc_day_morb_6m

This question means duration that the infant consumed zinc supplements in days.

1. 0
2. 1-3
3. 4-6
4. 7-9
5. 10+
97. NA

19) How many tablet(s)/day was your infant given zinc supplements?

zinc_tab_morb_6m

The important thing is the number of zinc supplements tablet consumed by the infant in a day.

1. ½
2. 1
3. 2
4. 3+
97. NA
98. Don't know

20. Has your child had an illness with a cough at any time in the last 2 weeks?

cough_morb_6m

This question means whether the infant had an illness with a cough at any time in the last 2 weeks or not.

1. Yes

0. No
98. Don't know
99. Missing

If No, then go to question number 22.

21. When your child had an illness with a cough in the last 2 weeks, did he/she breathe faster than usual with short, rapid breaths or have difficulty breathing?
apnea_morb_6m
The important thing is when the infant had an illness with a cough in the last 2 weeks, whether she breathe faster than usual with short, rapid breaths or have difficulty breathing or not. The purpose of this question is to determine the possibility of pneumonia cases.
1. Yes
0. No
97. NA
98. Don't know
99. Misssing

22) Has your infant had dengue fever?
dengue_morb_6m
This question means whether**infant has** had dengue fever **or not or don't know.**
1. Yes
0. No
98. Don't know
99. Missing

23. Has your child been feeding poorly or refusing food in the last 2 weeks?
refuse_eat_2w_morb_6m
This question means whether**infant has** been feeding poorly or refusing food in the last 2 weeks **or not or don't know.**
1. Yes
0. No
98. Don't know
99. Missing

24) Has your infant been given worm medicine in the past 6 months?
deworming_morb_6m
The important thing is whether the infant has been given worm medicine in the past 6 months or not. The consumption of worm medicine can eradicate the worms in infant gut.
1. Yes
0. No
98. Don't know
99. Missing

25) Has your infant been sick since born?
ever_sick_morb_6m
This question means whether**infant has** been sick since born **or not or don't know.**
1. Yes

0. No
98. Don't know
99. Missing

If No, then go to question number 53.

26. Did you seek advice for any of the illnesses from health workers?
seek_health_morb_6m
This question means whether mother seek advice for any of the illnesses from health workers or not.
1. Yes
0. No
97. NA
98. Don't know
99. Missing

If No, then go to question number 28.

27. If 1, which health workers you visited (most often)?
health_provider_morb_6m
The purpose of this question is to know which health workers that mother visited (most often). Please choose one. (NAME refers to child's name)
1. Doctor
2. Nurse
3. Midwife
4. Cadre
97. NA
99. Missing
66. Other

28. Currently, has (NAME) received any medications?
medicine_now_morb_6m
The important thing is whether (NAME) currently received any medications or not.
1. Yes
0. No
99. Missing

If No, then go to question number 30.

29. Could you please show the medication (if you still keeping the blister/package/bottle)?
medicine_list_morb_6m
Whether it is yes/No, please specify (name, dose, days of administration) of all medications received:
Ask the mother to specify the name, dose, days of administration of all medications received. Ask mother to show the medication if she still keeping the blister/package/bottle. The purpose of showing the blister/package/bottle is to

make sure the drug consumed by the infant. We can also know the dosage given to infant precisely.

30) Has (NAME) received any drug in the last 1 month?
medicine_1m_morb_6m
This question means whether(NAME) has received any drug in the last 1 month **or not or don't know.**
1. Yes
0. No
97. NA
99. Missing

If No, then go to question number 33

31) Has (NAME) received any antibiotics in the last 1 month?
ab_1m_morb_6m
Ask the mother whether (NAME) received or not any antibiotics in the last 1 month. Antibiotic consumption can influence gut micro bacteria examined in infant.
1. Yes
0. No
97. NA
98. Don't Know
99. Missing

32) Has (NAME) received any antifungal in the last 1 month?
af_1m_morb_6m
Ask the mother whether (NAME) received or not any antifungal in the last 1 month. Antifungal consumption can influence gut parasite examined in infant.
1. Yes
0. No
97. NA
98. Don't Know
99. Missing

33) Has (NAME) ever received antibiotics since born?
ab_ever_morb_6m
The important thing is whether (NAME) ever received antibiotics since born or not.
1. Yes
0. No
98. Don't Know
99. Missing

If No or Don't Know, then go to question number 38.

34) If 1, how many times (NAME) had ever received antibiotics since born?
ab_number_morb_6m

Ask the mother about the frequency of (NAME) antibiotic consumption period since born.
1. 1

2. 2
3. 3
4. 4+
97. NA
99. Missing

35) When is the last time (NAME) received antibiotics?
ab_last_morb_6m
Ask the mother
when the the last time (NAME) received antibiotics. You can fill in this question in date format or using adverb of time such as 2 weeks ago.

36) What is the name of the last antibiotics?
ab_name_morb_6m
Fill in this question with the name of the last antibiotics that (NAME) consumed since born.

37) How many days was the antibiotics given?
ab_long_morb_6m
Fill in this question with the duration of the last antibiotics that (NAME) consumed since born.

38) Has (NAME) ever received antifungal since born?
af_ever_morb_6m
The important thing is whether (NAME) ever received antifungal since born or not.
1. Yes
0. No
98. Don't Know
99. Missing

If No, then go to question number 43.

39) If 1, how many times (NAME) had ever received antifungal since born?
af_number_morb_6m

Ask the mother about the frequency of (NAME) antifungal consumption period since born.
1. 1
2. 2
3. 3
4. 4+
97. NA

40) When is the last time (NAME) received antifungal?
af_last_morb_6m

Ask the mother when the the last time (NAME) received antifungal. You can fill in this question in date format or using adverb of time such as 3 weeks ago.

41) What is the name of the antifungal?

af_name_morb_6m
Fill in this question with the name of the last antibiotics that (NAME) consumed since born.

42) How many days was the antifungal given?
af_long_morb_6m
Fill in this question with the duration of the last antifungal that (NAME) consumed since born.

143) Did you give the same number of breastfeeds and the same amount of complementary food during the time your baby was sick?
cf_sick_morb_6m
The important thing is mother give the same number of breastfeeds and the same amount of complementary food during the time her baby was sick or not.
1. Yes
0. No
97. NA
98. Don't Know
99. Missing

44) Do you give special foods to your infant when (NAME) is ill (in general)?
cf_add_morb_6m
Ask the mother whether she usually gives special foods to her infant when (NAME) is ill or not. Special foods including supplement.
1. Yes
0. No
97. NA
98. Don't Know
99. Missing

45) If yes, please specify:
cf_add_name_morb_6m
Ask the mother to specify what the special food is. Try to get the detail information.

46) Do you withhold certain food from your infant when (NAME) is ill (in general)?
cf_limit_morb_6m
Fill in this question whether mother withhold certain food from her infant when (NAME) is ill (in general) or not.
1. Yes
0. No
97. NA
98. Don't Know
99. Missing

47) If yes, please specify:
cf_limit_name_morb_6m
Ask the mother to specify what the certain food is. Try to get the detail information.

48) Do you give special foods to your infant after illness (in general)?

cf_after_sick_morb_6m

Fill in this question whether mother give special foods to her after illness (in general) or not.

1. Yes
0. No
97. NA
98. Don't Know
99. Missing

49 If 1, please specify:

cf_as_name_morb_6m

Ask the mother to specify what the special food is. Try to get the detail information.

50) Has your infant been hospitalised?

hospital_ever_morb_6m

The important thing is whether infant has been hospitalised or not or don't know.

1. Yes
0. No
98. Don't Know
99. Missing

If No, then go to question number 53

51If 1, how many times has your infant been hospitalised?

hospital_number_morb_6m

Ask the mother to specifyhow many times her infant has been hospitalised or don't know.

1. 1
2. 2
3. 3+
98. Don't know
99. Missing

52 Please specify the reason(s) for hospitalisation:

hospital_reason_morb_6m

Ask the mother to specifythe reason(s) for hospitalisation.

IMMUNISATION

53) Has your infant been immunised?

immunisation_morb_6m

The important thing is whether the infant has been immunized or not. It is very important that you ask each question exactly as it is written on the questionnaire. Even though the mother said only once, choose '1' choice button.

1. Yes

0. No
99. Missing

54) Can mother show the infant's immunisation card?
immunisation_card_morb_6m
Ask the mother whether mother can show the infant's immunisation card or not. Be sure that she understands that the "card" refers to vaccination documentation. In some cases the mother may not be willing to take time to look for the vaccination card, thinking that you are in a hurry. Encourage her to look for (NAME's) card. It is highly desirable to obtain written documentation of the child's immunization history; therefore, be patient if the respondent needs to search for the card.
1. Yes
0. No
99. Missing

If 1, please TAKE PICTURE of the IMMUNISATION CARD, and fill these questions based on the information in IMMUNISATION CARD! If NOT, Ask the mother when the interviewer can go back and see thatIMMUNISATION CARD. If there is not card, fill DONT KNOW for questions number 55-59.
155) Tuberculosis vaccination (BCG)?
bcg_morb_6m polio_morb_6m

See in the card whether the infant has received tuberculosis vaccination (BCG) or not.
1. Yes
0. No
97. NA
98. Don't know
99. Missing

56) Polio vaccination?
bcg_morb_6m polio_morb_6m

See in the card whether the infant has received Polio vaccination or not.
1. Yes
0. No
97. NA
98. Don't know
99. Missing

57) Diptheria, Pertussis and Tetanus (DPT) vaccination?
dpt_morb_6m
See in the card whether the infant has received Diptheria, Pertussis and Tetanus (DPT)or not..
1. Yes
0. No
97. NA
98. Don't know
99. Missing

58) Measles vaccination?

measles_morb_6m

See in the card whether the infant has received Measlesvaccination or not. 1. Yes

0. No
97. NA
98. Don't know
99. Missing

59 Hepatitis B vaccinations?

hep_b_morb_6m

See in the card whether the infant has received Hepatitis B vaccinations or not. 1. Yes

0. No
97. NA
98. Don't know
99. Missing

HYGIENE and SANITATION

60) Does your house get flooded when it rains very hard?

flood_morb_6m

Fill in this question whether house get flooded when it rains very hard or not. Get flooded means the floor inside the house is wet because of the rain.

1. Yes
0. No
99. Missing

61) What type of toilet facilities do members of your household use?

toilet_morb_6m

Ask the motherwhat type of toilet facilities is in the house. Pease choose one: private with septic tank, private with no septic tank, flush or pour flush toilet, shared/public, pit, river/stream/creek, yard or bush or forest. If there is another facility please write in the 'other 'column.

1. Private with Flush or pour flush toilet
2. Private with septic tank
3. Private with no septic tank
4. Shared/public (shared with others who are not family member)
5. Pit (private or public)
6. River/stream/creek
7. Yard or bush or forest
66. Other

62) What is the main source of drinking water for your household?

water_morb_6m

Ask the mother the main source of drinking water for her household is. Piped water into dwelling means piped water inside the house. Protected well in dwelling means the dwelling is covered to protect the hygiene of water. Bottled water means the water is bought by the mother.

1. Piped water into dwelling
2. Piped water into yard/plot
3. Piped water to public tap
4. Open well in dwelling
5. Open well in yard/plot
6. Open public well
7. Protected well in dwelling
8. Protected well in yard/plot
9. Protected public well
10. Water from spring
11. Water from rivers/stream
12. Pond/lake
13. Rain water
14. Bottled water
15. Ask from families or neighbors

163) What do you usually do to make the water safer to drink?
drink_morb_6m
Ask the mother what way that she usually does to make the water safer to drink. Choose one. If not mentioned in the choice, write the answer in 'specify'.

1. Boil
2. Add bleach or chlorine
3. Strain it through a cloth
4. Use water filter (ceramic or sand or composite)
5. Solar disinfection
6. Let it stand and settle
7. Directly drink it
8. Strain it first then boil it
9. Let it settle first then boil it

98\. Don't know
66\. Other drink_othr_morb_6m

64) At any time in the past 12 months, has anyone come into your house to spray the inside walls against mosquitoes?
fogging_morb_6m
Fill in the question whether the house has been fogged inside walls against mosquitoes or not. If mother doesn't know, choose 'don't know'.

1\. Yes
0\. No
98\. Don't know
99\. Missing

65) Does your household have any insecticide treated mosquito nets that can be used while sleeping?
bednet_morb_6m

Fill in the question whether household have any insecticide treated mosquito nets that can be used while sleeping or not.

1. Yes
0. No
98. Don't know
99. Missing

If No, then go to question number 67

66)) If 1, did your infant sleep under an insecticide treated mosquito net last night?

bednet_child_morb_6m

The important of this question is whether the infant sleep under an insecticide treated mosquito net last night or not.

1. Yes
0. No
97. NA
98. Don't know
99. Missing

67) Does anyone regularly smoke cigarettes or use any other type of tobacco in the house?

smoke_morb_6m

The important of this question is whether there is anyone that the regularly smoke cigarettes or use any other type of tobacco in the house or not.

1. Yes
0. No
99. Missing

68) Do you use an open fire for cooking in the house?

firewood_morb_6m

The important of this question is whether mother use an open fire for cooking in the house or not.

1. Yes
0. No
99. Missing

69) Who usually cooks your infant's food?

cook_morb_6m

Ask the mother who usually cooks the infant's food to fill in this question. Remember to choose only one.

1. Myself (mother)
2. Mother in law
3. Mother
4. Caregiver
5. Sibling
6. Husband
66. Other

70) Who usually feeds your infant?:

feed_morb_6m

Ask the mother who usually feeds her infant. Please choose one answer.

1. Myself (mother)
2. Mother in law
3. Mother
4. Caregiver
5. Sibling
6. Husband
66. Other

71) Do you always cover food until it is eaten?

food_cover_morb_6m

Fill in the question whether mother always cover food until it is eaten or not.

1. Yes
0. No
99. Missing

72) There are key moments when you need to wash your hands to prevent germs from reaching food. What are these key moments?

In this question you can choose more than one answer. Fill in the key moments when mother need to wash her hands to prevent germs from reaching food.

1. After going to the toilet/latrinewash_toilet_morb_6m
2. After cleaning the baby's bottom/changing a baby's nappywash_diaper_morb_6m
3. Before preparing/handling foodwash_cook_morb_6m
4. Before feeding a childwash_feed_morb_6m
5. Before eatingwash_eat_morb_6m
6. After handling raw foodwash_raw_morb_6m
7. After handling garbagewash_trash_morb_6m
98. Don't know wash_dk_morb_6m
66. Other wash_othr_morb_6m

73) Do you have pet(s) at home?

pet_morb_6m

The important thing is whether mother has pet(s) or not at home.

1. Yes
0. No
99. Missing

74) If 1, please specify:

pet_name_morb_6m

Fill in this question with any pet that mother has.

PART 3 - BREASTFEEDING and INFANT YOUNG CHILDREN FEEDING

---

1) Interviewer:*
interviewer_breast_6m
Use your initial every time you have to fill in the interviewer question.
1. AD : Aly Diana
2. EI : Eva Indirawati
3. M : Monik
4. DEL : Dimas Erlangga Luftimas
5. R : Rima
6. H : Hendro
66. Other

2) Mother's name:*
mom_name_breast_6m
It is better to fill in the full name of mother. If it is not possible, you can fill in the forename, middle name or nick name. Please match the name that was told by mother with the name written in the paper.

3) Mother's ID number:*
mom_id_breast_6m
It is no need of asking this question. It is written in the paper given to you.

4) Who is responding?
response_breast_6m
You have to ask who is someone that answering your question. Is she infant's mother or the other, please specify and fill in this question.
1. Yes
2. Mother (infant's grandmother)
3. Husband
4. Sibling (of mother)
66. Other, othr_breast_6m
99. Missing

5) How long after birth did you first put (NAME) to the breast?
bf_breast_6m
Ask the mother how long after birth her infant first try to breastfeed.
1. Right after the baby was born (less than an hour)
2. More than an hour after the baby was born
98. Don't know
99. Missing

6) During the first three days after delivery, did you give (NAME) the yellow liquid that came from your breasts?
colostrum_breast_6m

For approximately three days after delivery, the breasts secrete colostrum. There are some communities that believe colostrum is not good for infants and do not allow them to have colostrum. Fill in this question whether in the first three days after delivery mother give (NAME) the yellow liquid that came from her breast or not. (NAME refers to child's name)

1. Yes
0. No
98. Don't know
99. Missing

7) *During the first three days after delivery, did you give (NAME) anything else to eat or drink before feeding him/her breast milk? Please specify:*

Ask the mother to specify any foods that she gave to the infant during the first three days after delivery. You can click more than one choice if mother said giving infant more than one king of foods beside breast milk.

1. Honeyhoney_breast_6m
2. Milk (other than breast milk)milk_breast_6m
3. Infant formula formula_breast_6m
4. Butter butter_breast_6m
5. Plain water water_breast_6m
6. Water with sugar and/or salt (excluded ORS) sweet_breast_6m
7. Fruit juice juice_breast_6m
8. Tea or coffeetea_breast_6m
9. Nothingnothing_breast_6m
66. Other othr2_breast_6m

8) Did (NAME) drink anything from a bottle with a nipple 1terday or last night?
bottle_breast_6m
Ask mother whether the infant or child drank anything from a bottle with a nipple 1terday. Baby bottles are not recommended, because they are very difficult to clean. Spoons, cups, and gourds may also be dirty, but bottles are even more likely to be contaminated and to make the baby sick. Fill in this question whether (NAME) drank anything from a bottle with a nipple 1terday or last night or not.

1. Yes
0. No
98. Don't know
99. Missing

9) Was (NAME) breastfed of did he/she consume breastmilk during the day or at night?
yest_breast_6m
Fill in this question whether the infant was breastfed during all day or not.

1. Yes
0. No
98. Don't know
99. Missing

10) If No, for how long did you breastfeed (NAME)? (in months)
long_breast_6m
Ask the mother how long she breastfeed (NAME) in month.

11) Why did you stop breastfeeding?

stop_breast_6m

The important thing is the reason why the mother stopped breastfeeding. Then fill in this question.

1. Mother pregnant
2. Mother unwell
3. Mother tired of breastfeeding
4. ntroduced solids
5. Breast milk making child sick
6. Not enough milk
7. Infant refused breast milk
8. Formula milk is better
66. Other
97. NA
98. Don't know
99. Missing

12) Have you started to introduce solid foods (complementary feeding) to your infant?

cf_breast_6m

Fluids and/or semi-solids given to infants in the first few days after delivery are called prelacteal feeds. They may introduce pathogens that cause diarrhea and other diseases.

Prelacteal feeds are given during the period when the colostrum is produced prior to the free flow of the mother's mature milk and rob the infant of this important health protecting substance. Fill in this question whether mother have starte to introduce solid foods (complementary feeding) to her infant or not.

1. Yes
0. No
98. Don't know
99. Missing

13) What made you decide to start giving solid foods to (NAME)?

reason_breast_6m

Ask the mother about the reason why the mother start giving solid foods to (NAME).

1. Not enough breast milk
2. Weak (low quality) of breast milk
3. Breastmilk alone is not sufficient (enough)/cannot supply all the nutrients needed for growth
4. Not enough time to breastfeed
5. Baby is always crying
6. Baby reaching for food
7. Baby active
8. Tradition
9. Told to
10. Necessity to work
11. Common knowledge, the age is already required solid foods
12. Baby shows his/her interest when looking others eating
13. Often hear the baby's stomach growling

14. Baby always put everything on his/her mouth
98. Don't know
66. Otherothr4_breast_6m

14) How old was (NAME) when you started giving him/her semi-solid/solid foods other than breast milk?
cf_age_breast_6m *Ask the mother about the age of (NAME) when you started giving him/her semi-solid/solid foods other than breast milk.*
1. 1 month
2. 2 month
3. 3 month
4. 4 month
5. 5 month
6. 6+ month
98. Don't know

Now I would like to ask you about other foods your child ate over the last 24 hours (question 19-25). I am interested in whether your child had the item even if it was combined with other foods. 1terday, did your child eat:

15) Grains, roots and tubers?
Some foods in the list are listed as a single item – for example, white potatoes – but may usually be eaten in a soup or stew. If the infant or child has eaten a mixed food like a soup,or stew, record all the food groups in the mixed food. For example, if the child ate a stew ofpotatoes, maize, there should be a check mark for each of the two food groups that contain these foods. Do not check off foods that have been added in very small amounts, or for seasoning.

	Code	1 Yes	0 No	99 Missing
Porridge, bread, rice, maize, noodles or other foods made from grains	rice_breast_6m			
White potatoes, white yams, cassava or any other foods made from roots	root_breast_6m			

16) Any foods made from beans (kidney beans, mung beans, soy beans, tofu, tempeh), peas, lentils, nuts (peanuts) or seeds?
nuts_breast_6m

The important thing is whether infant eat any foods made from beans (kidney beans, mung beans, soy beans, tofu, tempeh), peas, lentils, nuts (peanuts) or seeds or not. Do not check off foods that have been added in very small amounts, or for seasoning.
1. Yes
0. No
99. Missing

17) Any dairy products?

Some foods in the list are listed as a single item. If the infant or child has eaten a mixed food record all the food groups in the mixed food. Please mark '1' or 'no' button

		1 Yes	0 No	99 Missing
Milk, such as tinned, powdered or fresh animal milk	milk2_breast_6m			
Formula milk	formula2_breast_6m			
Yogurt or drinking yogurt	yoghurt_breast_6m			
Cheese or other dairy products	cheese_breast_6m			

18) Any flesh foods?

Some foods in the list are listed as a single item. If the infant or child has eaten a mixed food record all the food groups in the mixed food. Please mark '1' or 'no' button. Do not check off foods that have been added in very small amounts, or for seasoning. For example, if a spoon of fish powder is added to a pot of stew, do not record that the infant or child has eaten fish

	Code	1 Yes	0 No	99 Missing
Liver, kidney, heart or other organ meats	organ_breast_6m			
Any meat, such as beef, pork, lamb, goat, chicken or duck	flesh_breast_6m			
Fresh or dried fish, shellfish or seafood	seafood_breast_6m			

19) Chicken eggs, duck eggs or qual eggs?

egg2_breast_6m

The important thing is whether the infant eat chicken eggs, duck eggs or qual eggs or not.

1. Yes
0. No
99. Missing

20) Yellow/red/orange fruits and/or any dark green leafy vegetables?

Some foods in the list are listed as a single item – for example, pumpkin – but may usually be eaten in a soup or stew. If the infant or child has eaten a mixed food

like a soup,or stew, record all the food groups in the mixed food. For example, if the child ate a stew ofpumpkin, papaya, and green leaves, there should be a check mark for each of the three foodgroups that contain these foods. Do not check off foods that have been added in very small amounts, or for seasoning. For example, If one fifth of tomato is included in the family pot, do not record that as an “other fruit or vegetable.”

		1 Yes	0 No	99 Missing
Pumpkin, carrots, tomatoes, squash or sweet potatoes that are yellow or orange inside	yellow_breast_6m			
Any dark green leafy vegetables, e.g. spinach, cassava leaves, water spinach, broccoli, caweightage, lettuce	green_breast_6m			
Ripe mangoes (fresh or dried), ripe papayas	orange_breast_6m			

21) Any other foods mentioned below?

Some foods in the list are listed as a single item – for example, biscuit – but may usually be eaten together such as buttered biscuit. If the infant or child has eaten a mixed food like buttered biscuit, record all the food groups in the mixed food. For example, if the child ate a buttered biscuit, there should be a check mark for each of the two food groups that contain these foods. Do not check off foods that have been added in very small amounts, or for seasoning. For example, If one chili pepper is included in the family pot, do not record that as an “other fruit or vegetable.”

	Code	1 Yes	0 No	99 Missing
Any oils, fats, or butter or foods made with any of these	oil_breast_6m			
Any surgary foods, such as chocolates, sweets, candies, pastries, cake or biscuits	sugar_breast_6m			
Any sweet beverage, such as soda, fruit flavoured drink	beverage_breast_6m			
Any tea and/or coffee	coffee_breast_6m			
Condiments for flavour, such as chilies, spices, herbs, soy	spice_breast_6m			

sauce, ketchup, or fish powder				

22) How many times did (NAME) eat foods, that is meals and snacks other than liquids yesterday during the day or at night?
eat_breast_6m
This question asks about how many times the infant or child ate solid/semi-solid foods as meals and snacks 1terday. Ask the mother/caregiver the question just as it is written. Solid/semi-solid foods include family foods, and also many special dishes prepared for infants. Thick soups and stews should be included. Thick porridges are also included. Very thin, watery soups and gruels should not be included because infants and young children do not get enough energy (calories) from very thin soups and gruels. Liquids do not count for this question. Also, very small snacks, such as a bite or two of someone else's food, should not be counted.
1. 1
2. 2
3. 3
4. 4
5. 5
6. 6+
97. NA

23) How many meals (excluding snacks) did you feed your infant 1terday?
meal_breast_6m
This question asks about how many times the infant or child ate solid/semi-solid foods as meals 1terday. Ask the mother/caregiver the question just as it is written. Solid/semi-solid foods include family foods, and also many special dishes prepared for infants.
1. 1 meals/day
2. 2 meals/day
3. 3 meals/day
4. 4 meals/day
5. 5 meals/day
6. 6+ meals/day
97. NA
98. Don't know

24) Did your child eat any food prepared with flour 1terday during the day or night?
flour_name_breast_6m
This question asks whether infant ate or not any food prepared with flour that was fortified with micronutrients yesterday.
1. Yes, please write the brand:
0. No
97. NA
98. Don't know
99. Missing

25) Has your infant ever received salt fortified by iodine?

salt_breast_6m

This question asks whether infant ever received salt fortified by iodine or not. Iodine is important for children growth and intelligent. Iodine deficiency also can cause endemic goiter.

1. Yes
0. No
98. Don't know
99. Missing

26) Please show the salt used in your household. Write down the brand:

salt_name_breast_6m

This question asks the mother to show the salt used in her household. Write down the brand.

SUPPLEMENTATION

27) Has your infant ever received Vitamin A supplements in the last 6 months? (Please show vitamin A capsules)

vit_a_breast_6m

The purpose of question 31 is to find out whether or not the infant/child received a doseof vitamin A in the last 6 months. Lack of vitamin A causes children to get sick more easily andin extreme cases lack of vitamin A can cause eye damage and blindness. Vitamin A supplements are given because many children do not get enough vitamin A from the foods theyeat.

You will need examples of the vitamin A capsules or ampules that have been used in the project area. Show the mother/caregiver the capsule or ampule to help her remember if her infant or child ever received one.

1. Yes
0. No
98. Don't know
99. Missing

28) Has your infant received multivitamin and minerals pills or syrup in the last month?

multivitamin_breast_6m

The purpose of question 31 is to find out whether or not the infant/child received a dose of multivitamin and minerals pills or syrup in the last month.

1. Yes
0. No
98. Don't know
99. Missing

29) Has your infant received sprinkles (TABURIA) in the last 6 months? (Please show TABURIA)

taburia_breast_6m

This question asks whether infant has received sprinkles (TABURIA) in the last 6 months or not. (Please show TABURIA)

1. Yes
0. No
98. Don't know
99. Missing

If No or Don't Know , then go to question number 37

30) From whom have you received the sprinkles (TABURIA)?

taburia_who_breast_6m

The purpose of question 34 is to find out from whothe mother has received the sprinkles (TABURIA).

1. Puskesmas staff
2. Village Midwife
3. Cadres
66. Other
97. NA
99. Missing

31) When did you receive it?

taburia_when_breast_6m

The purpose of question 35 is to find out the time that mother has received the sprinkles (TABURIA). TABURIA better been given at 6 month until 24 month old infant.

1. This month
2. Last month
3. Two months ago
4. Three months ago
5. Four months ago (or more)
97. NA
99. Missing

32) How many sachets of sprinkles have you received in the last 6 months?

taburia_number_breast_6m

The purpose of question 36 is to find out how many sachets of sprinkles mother has received in the last 6 months

1. <15 sachets
2. 15 – 30 sachets
3. 30 – 60 sachets
4. >60 sachets
97. NA
98. Don't know
99. Missing

33) How many sachets of sprinkles have you given to (NAME) in the last 6 months?

taburia_eat_breast_6m

The purpose of question 37 is to find out how many sachets of sprinkles mother give to (NAME) in the last 6 months.

1. <5 sachets
2. 5 – 10 sachets
3. 10 – 15 sachets
4. >15 sachets
97. NA
99. Missing

34) How did you give the sprinkles to (NAME)?

This question asks how mother give the sprinkles to (NAME). It should be given once every two days.

1. Twice a day taburia_twice_breast_6m
2. Every day taburia_once_breast_6m
3. Every other day taburia_days_breast_6m
4. Sprink it into infant porridge (cool) taburia_cold_breast_6m
5. Sprink it into infant porridge (hot) taburia_hot_breast_6m
6. Mix it with porridge/soup while cooking taburia_cook_breast_6m
7. Never taburia_never_breast_6m

97. NA taburia_na_breast_6m
66. Other othr6_breast_6m

35) How did (NAME) react to the addition of sprinkles (Taburia) to his/her food?
taburia_reaction_breast_6m
This question asks how (NAME) did react to the addition of sprinkles (Taburia) to his/her food.

1. Dislike very much
2. Dislike a little
3. Not sure
4. Like a little
5. Like very much

97. NA

36) Do you know what is the function/benefit of giving sprinkles to your infant? Please mention:
taburia_benefit_breast_6m
The purpose of question 40 is to find out the knowledge of mother about the function/benefit of giving sprinkles to infant.

---

37) Have you regularly brought your infant to Posyandu?
posyandu_breast_6m
The purpose of question 44 is to find out whether mother regularly brings her infant to Posyandu or not.

1. Yes, once per month
2. Yes, once every two month
3. Sometimes (1-2 times)
4. Never

98. Don't know

38) What kind of cooking pot do you use?
This question means what kind of cooking pot mother use. This is important because any pot material contain harmful substance for infant.

1. Clay clay_breast_6m
2. Aluminium alumunium_breast_6m
3. Steel steel_breast_6m
4. Iron iron_breast_6m

98. Don't Know dk_breast_6m
97. NA na_breast_6m
99. Missing

39) Do you ever soak your beans before cooking?

soak_breast_6m

The purpose of question 47 is to find out whether mother soaksthe beans before cooking or not. Soaking the beans is important to reduce vitat that inhibit vitamin absorption. Reference

1. Yes
0. No
98. Don't know
99. Missing

40) If 1, why do you soak your beans before cooking?

soak_yes_breast_6m

The purpose of question 48 is to find out the reason why mother soak the beans before cooking.

1. Traditional
2. Improves taste
3. Healthier
4. Reduces cooking time
5. Told to do it
6. To make it become more tender
98. Don't know
97. NA
66. Other

41) Why do you NOT soak your beans before cooking?

soak_no_breast_6m

The purpose of question 48 is to find out the reason why mother didn't soakthe beans before cooking.

1. Too time consuming
2. Spoils taste
3. Told not to do it
4. Lack of knowledge/experience
98. Don't know
97. NA
66. Other

MEAL PLANNING

42) How many snacks did you feed your infant yesterday?

snack_breast_6m

This question asks about how many times the infant or child ate solid/semi-solid foods as snacks 1terday. Ask the mother/caregiver the question just as it is written. Solid/semi-solid foods include family foods, and also many special dishes prepared for infants.

1. 1 snacks/day
2. 2 snacks/day

3. 3 snacks/day
4. 4 snacks/day
5. 5 +snacks/day
98. Don't know
97. NA

43) Do you give your children a separate plate?

plate_breast_6m

The important thing is whether mother givesher children a separate plate or not.

1. Yes
0. No
97. NA
98. Don't know
99. Missing

44) Did you add ingredients to your infant's porridge such as mashed vegetables, beans, fruits, eggs, and small fish?

porridge_add_breast_6m

The important thing is whether mother add ingredients to the infant's porridge such as mashed vegetables, beans, fruits, eggs, and small fish or not.

1. Yes
0. No
97. NA
98. Don't know
99. Missing

45) During the last two weeks did your infant eat any of the following foods? (ALLOW MULTIPLE ANSWERS, READ THE LIST)

The important thing is whether during the last two weeks infant eat any foods listed in the question. Encourage mother to multiple answers, but remember not to mention the foods listed.

1. Cassava leaves cassava_leaf_breast_6m
2. Pumpkin leaves pumpkin_leaf_breast_6m
3. Carrots carrot_breast_6m
4. Pumpkin pumpkin_breast_6m
5. Groundnuts nuts2_breast_6m
6. Potato leaves potato_leaf_breast_6m
7. Mangoes mango_breast_6m
8. Papaya papaya_breast_6m
9. Eggs egg3_breast_6m
10. Meat flesh2_breast_6m
11. Fish fish_breast_6m
12. Banana banana_breast_6m
13. Avocado avocado_breast_6m
14. Sweet potato sweet_potato_breast_6m
15. Animal milk milk3_breast_6m
98. Don't know dk_breast_6m
97. NA na_breast_6m

46) 47) Are you the person who fed [NAME] yesterday?
mom_feed_breast_6m

The purpose of question58 is to confirm whether mother was someone that fed the infant 1terday or not.

1. Yes
0. No
97. NA
99. Missing

47) 48) Yesterday, at the main meal, did [NAME] eat all the food you thought he/she should?
eat_all_breast_6m

The purpose of question59 is whether [NAME] eat all the food mother's thought he/she should or not.

1. Yes
0. No
97. NA
98. Don't know
99. Missing

48)) During this meal, were you or another caregiver sitting with your infant?
sit_together_breast_6m

This question asks whether mother or another caregiver were sitting with infant during this meal or not.

1. Yes
0. No
97. NA
99. Missing

49) If sitting with the infant, how were you two positioned?
position_breast_6m

The question asks how about the position of mother/or caregiver to infant.
Whether can see the infant's face or not.

1. Mother/caregiver positioned so she could NOT see infant's face
2. Mother/caregiver positioned so she could see infant's face frontways or sideways
97. NA
99. Missing

50)) How was the infant positioned?
infant_position_breast_6m

The question asks how about the position of infant.

1. Infant held in mother or caregiver's arms
2. Infant lying in mother or caregiver's lap
3. Infant sitting in mother or caregiver's lap
4. Infant standing up or running around
5. Infant sitting on a chair, stool, mat or bed
6. Infant lying on the floor or bed

97 . NA

66. Otherposition_othr_breast_6m

51)) Did your infant refuse to eat an offered mouthful of food?
refuse_breast_6m

This question asks whether infant refused to eat an offered mouthful of food or not.

1. Yes
0. No
97. NA
99. Missing

52)) Did your infant cry during the meal 1terday?
cry_breast_6m

This question asks whether i infant cried during the meal 1terday or not

1. Yes
0. No
97. NA
99. Missing

53) Did your infant try to leave the meal early yesterday?
leave_early_breast_6m

This question asks whether infant try to leave the meal early 1terday or not

1. Yes
0. No
97. NA
99. Missing

54) Did you have to control your infant while eating, such as by holding his/her head or body?
control_breast_6m

The purpose of question70 is whether mother has to control your infant while eating, such as by holding his/her head or body.

1. Yes
0. No
97. NA
99. Missing

55) yesterday at the evening meal, how was your infant behaviour?
behaviour_breast_6m

The purpose of question71 is to find out infant behaviour on yesterday at the evening meal.

1. More fussy about eating than usual
2. Eating as she or he usually eats
3. Less fussy about eating than usual
98. Don't know
97. NA

56) Was your infant sick yesterday?

sick_breast_6m

This question asks whether infant sick yesterday or not.

1. Yes
0. No
99. Missing

57) Is your child able to feed themselves some of the meal?

feed_themself_breast_6m

This question asks whether infant able to feed themselves some of the meal or not

1. Yes
0. No
99. Missing

58) If 1, at what age in months are children able to START feeding themselves some of the meal?

feed_themself_age_breast_6m

The purpose of question74 is to find out the specific age in months the children able to START feeding themselves some of the meal.

---

59) How long is it recommended that a woman breastfeed her child? (Probe if necessary: Until what age is it recommended that a mother continues breastfeeding?)

long_breastfeed_breast_6m

The purpose of question75 is to find out the knowledge of mother about duration recommended that a woman breastfeed her child.

1. Less than 6 months
2. 6 months
3. 7-11 months
4. 12-23 months
5. 24+ months
98. Don't know
66. Otherlong_othr_breast_6m

60) At what age should babies start eating foods in addition to breastmilk?

start_eat_breast_6m

This question asks the knowledge of mother about at what age babies should start eating foods in addition to breastmilk

1. 6 months
98. Don't know
66. Otherstart_othr_breast_6m

61) Please look at these two pictures of porridges. Which one do you think should be given to a young child?

porridges_breast_6m

For this question, you have to show to mother the two pictures of porridges. Then ask the mother which one mother think should be given to a young child.

1. Thick porridge
2. Watery porridge

98. Don't know

62) Why did you pick that picture?
reason_breast_6m

The purpose of question78 is to find out the reason why mother has chosen one of two pictures.

1. Because the first porridge is thicker than the other
2. Because the thick porridge is more nutritious or because it is prepared with different types of foods or ingridients (food diversity)
3. Because watery porridge is easier to be swallowed
4. Because it is more difficult to feed when the porridge is too watery
5. Because watery porridge is easier to be digested
6. Because the infant's preference
7. To train infant swallowing her/his food
8. In order to make infant full easily
9. Because infant tends to throw upwhen the porridge gets too thick
10. Because it becomes a habit to make porridge that way
11. Because they are told that one is better than another
12. Because watery porridge can make infant bloating

98. Don't know
66. Otherreason_othr_breast_6m

63) To feed their children, many mothers give them rice porridge. Please tell me some ways to make rice porridge more nutritious or better for your baby's health. (Probe if necessary: Which foods or types of food can be added to rice porridge make it more nutritious?)
animal_breast_6m

For this question, you have to ask the mother to explain some ways to make rice porridge more nutritious or better for her baby's health.

---

1. Animal-source foods (meat, poultry, fish, liver/organ meat, eggs, etc.)animal_breast_6m
2. Pulses and nuts: flours of groundnut and other legumes (peas, beans, lentils, etc.), sunflower seed, peanuts, soybeansnuts_breast_6m
3. Vitamin-A-rich fruits and vegetables (carrot, orange-fleshed sweet potato, yelow pumpkin, mango, papaya, etc) fruits_veggies_breast_6m
4. Green leafy vegetables (e.g. spinach)green_leaves_breast_6m
5. Energy-rich foods (e.g. oil, butter)butter_breast_6m

66. Other, please specify: _______food_othr_breast_6m
98. Don't knowfood_dk_breast_6m

PART4–Ages and Stages

1)Interviewer:*
interviewer_stages_6m

Use your initial every time you have to fill in the interviewer question.
1. AD : Aly Diana
2. EI : Eva Indirawati
3. M : Monik
4. DEL : Dimas Erlangga Luftimas
5. R : Rima
6. H : Hendro

66. Other

2) Mother's name:*
name_mom_stages_6m

*It is better to fill in the full name of mother. If it is not possible, you can fill in the forename, middle name or nick name. Please match the name that was told by mother with the name written in the paper**.*

3) *Mother's ID number:**
id_stages_6m

*It is no need of asking this question. It is written in the paper** given to you. Please re-check once again whether you have typed the ID number correctly.*

Communication

4) Does your baby make high pitch squeals?
q1_voice_high_stages_6m
1. Yes
2. Sometimes
0. No

5) Observed/reported
1. Observedq1_observed_stages_6m
2. Reportedq1_reported_stages_6m
3. There is difference between observation result and what was reportedq1_different_stages_6m

6) When playing with sounds, does your baby make grunting, growling or other deep toned sounds?
q2_voice_low_stages_6m

1. Yes
2. Sometimes
0. No

7) Observed/reported
1. Observedq2_observed_stages_6m
2. Reportedq2_reported_stages_6m

3. There is difference between observation result and what was

   reportedq2_different_stages_6m

8) If you call your baby when you are out of sight, does he look in the direction of your voice?
q3_calling_name_stages_6m

1. Yes
2. Sometimes
0. No

9) Observed/reported
1. Observedq3_observed_stages_6m
2. Reportedq3_reported_stages_6m
3. There is difference between observation result and what was

   reportedq3_different_stages_6m

10) When a loud noise occurs, does your baby turn to see where the sound came from?
q4_loud_noise_turn_stages_6m

1. Yes
2. Sometimes
0. No

11) Observed/reported
1. Observedq4_observed_stages_6m
2. Reportedq4_reported_stages_6m
3. There is difference between observation result and what was

   reportedq4_different_stages_6m

12) Does your baby makes sounds like "da", "ga", "ka", and "ba"?
q5_sounds_da_stages_6m

1. Yes
2. Sometimes
0. No

13) Observed/reported
1. Observedq5_observed_stages_6m
2. Reportedq5_reported_stages_6m
3. There is difference between observation result and what was

   reportedq5_different_stages_6m

14) If you copy the sounds your baby makes, does your baby repeat the same sounds back to you?
q6_repeat_sounds_stages_6m
1. Yes
2. Sometimes
0. No

15) Observed/reported
1. Observedsq6_observed_stages_6m
2. Reportedq6_reported_stages_6m
3. There is difference between observation result and what was reportedq6_different_stages_6m

Gross motor

16) While your baby is on his back, does your baby lift his legs high enough to see his feets?
q7_lift_legs_stages_6m
1. Yes
2. Sometimes
0. No

17) Observed/reported
1. Observedq7_observed_stages_6m
2. Reportedq7_reported_stages_6m
3. There is difference between observation result and what was reportedq7_different_stages_6m

18) When your baby is on his tummy, does she straighten both arms and push her whole chest off the bed or floor?
q8_lift_chest_stages_6m
1. Yes
2. Sometimes
0. No

19) Observed/reported
1. Observedq8_observed_stages_6m
2. Reportedq8_reported_stages_6m
3. There is difference between observation result and what was reportedq8_different_stages_6m

20) When your baby on the floor, does he lean on her hands while sitting? (if he already sits up straight without leaning on her hands, mark yes for this item)
q9_sit_lean_hands_stages_6m

1. Yes
2. Sometimes
0. No

21) Observed/reported
1. Observeds q9_observed_stages_6m
2. Reportedq9_reported_stages_6m
3. There is difference between observation result and what was reportedq9_different_stages_6m

22) If you hold both hands just to balance your baby, does he support his own weight while standing?
q10_balance_standing_stages_6m

1. Yes
2. Sometimes
0. No

23) Observed/reported
1. Observedq10_observed_stages_6m
2. Reportedq10_reported_stages_6m
3. There is difference between observation result and what was reportedq10_different_stages_6m

24) Does your baby get into crawling position by getting up on her hands and knees?
q11_crawl_stages_6m

1. Yes
2. Sometimes
0. No

25) Observed/reported
1. Observedq11_reported_stages_6m
2. Reportedq11_different_stages_6m
3. There is difference between observation result and what was reportedq11_observed_stages_6m

Fine motor

26) Does your baby grab a toy you offer and look at it, wave it about, or chew on it about 1 minute?
q12_hold_toy_1minute_stages_6m

1. Yes
2. Sometimes
0. No

27) Observed/reported
1. Observedq12_reported_stages_6m
2. Reportedq12_different_stages_6m

3. There is difference between observation result and what was reportedq12_observed_stages_6m

28) Does your baby reach or grasp a toy using both hands at once?
q13_grasp_by_2hands_stages_6m

1. Yes
2. Sometimes
0. No

29) Observed/reported

1. Observed q13_observed_stages_6m
2. Reportedq13_reported_stages_6m
3. There is difference between observation result and what was reportedq13_different_stages_6m

30) Does your baby reach for a crumb or cheerio and touch it with his finger or hand? (if he already picks up a small object the size of a pea, mark "yes" for this item)
q14_reach_crumbs_stages_6m

1. Yes
2. Sometimes
0. No

31) Observed/reported

1. Observedq14_observed_stages_6m
2. Reportedq14_reported_stages_6m
3. There is difference between observation result and what was reportedq14_different_stages_6m

32) Does your baby pick up a small toy, holding it in the center of her hand with his finger around it?
q15_hold_center_hand_stages_6m

1. Yes
2. Sometimes
0. No

33) Observed/reported

1. Obsevedq15_observed_stages_6m
2. Reportedq15_reported_stages_6m
3. There is difference between observation result and what was reportedq15_different_stages_6m

34) Does your baby try to pick up a crumb or cheerio by using his thumband all of his fingers in a raking motion, even if he isnt able to pick it up? (if he already picks up a crumb or cheerio, mark yes for this item)
q16_pickup_crumbs_raking_stages_6m

1. Yes
2. Sometimes
0. No

35) Observed/reported
1. Observedq16_observed_stages_6m
2. Reportedq16_reported_stages_6m
3. There is difference between observation result and what was reportedq16_different_stages_6m

36) Does your baby pick up a small toy with only one hand?
q17_pickup_by_1hand_stages_6m

1. Yes
2. Sometimes
0. No

37) Observed/reported
1. Observedq17_observed_stages_6m
2. Reportedq17_reported_stages_6m
3. There is difference between observation result and what was reportedq17_different_stages_6m

Problem solving

38) When a toy is in front of your baby, does he reach for it with both hands?
q18_reach_by_2hands_6m

1. Yes
2. Sometimes
0. No

39) Observed/reported
1. Observedq18_observed_stages_6m
2. Reportedq18_reported_stages_6m
3. There is difference between observation result and what was reportedq18_different_stages_6m

40) When your baby is in his back, does he turn his head to look for a toy when he drops it? (if he already picks it up, mark "yes" for this item)
q19_search_toy_dropped_stages_6m

1. Yes
2. Sometimes

0. No

41) Observed/reported

1. Observedq19_observed_stages_6m
2. Reportedq19_reported_stages_6m
3. There is difference between observation result and what was reportedq19_different_stages_6m

42) When your baby is on her back, does he try to get a toy he has dropped if he can see it?
q20_get_toy_dropped_stages_6m

1. Yes
2. Sometimes
0. No

43) Observed/reported

1. Observedq20_observed_stages_6m
2. Reportedq20_reported_stages_6m
3. There is difference between observation result and what was reportedq20_different_stages_6m

44) Does your baby pick up a to and put it in his mouth?
q21_put_toy_mouth_stages_6m

1. Yes
2. Sometimes
0. No

45) Observed/reported

1. Observedq21_observed_stages_6m
2. Reportedq21_reported_stages_6m
3. There is difference between observation result and what was reportedq21_different_stages_6m

46) Does your baby pass s toy back and forth from one hand to the other?
q22_pass_hand_stages_6m

1. Yes
2. Sometimes
0. No

47) Observed/reported

1. Observedq22_observed_stages_6m
2. Reportedq22_reported_stages_6m
3. There is difference between observation result and what was reportedq22_different_stages_6m

48) Does your baby play by banging toy up and down on the floor or table?

q23_banging_toy_stages_6m
1. Yes
2. Sometimes
0. No

49) Observed/reported
1. Observedq23_observed_stages_6m
2. Reportedq23_reported_stages_6m
3. There is difference between observation result and what was reportedq23_different_stages_6m

Personal social

50) When in front of large mirror, does your baby smile or coo at himself?
q24_mirror_smile_stages_6m
1. Yes
2. Sometimes
0. No

51) Observed/reported
1. Observedq24_observed_stages_6m
2. Reportedq24_reported_stages_6m
3. There is difference between observation result and what was reportedq24_different_stages_6m

52) Does your baby act differently towards strangers than he does with you and other famiiiliar people? (reactions to stranger may include staring, frowning, withdrawing, or crying)
q25_reaction_stangers_stages_6m
1. Yes
2. Sometimes
0. No

53) Observed/reported
1. Observedq25_observed_stages_6m
2. Reportedq25_reported_stages_6m
3. There is difference between observation result and what was reportedq25_different_stages_6m

54) While lying on his back, does your baby ply by grabbing her foot?
q26_grab_foot_stages_6m
1. Yes
2. Sometimes
0. No

55) Observed/reported
1. Observedq26_observed_stages_6m

2. Reportedq26_reported_stages_6m
3. There is difference between observation result and what was reportedq26_different_stages_6m

56) When in front of large mirror, does your baby reach out to pat the irror?
q27_mirror_pat_stages_6m

1. Yes
2. Sometimes
0. No

57) Observed/reported
1. Observedq27_observed_stages_6m
2. Reportedq27_reported_stages_6m
3. There is difference between observation result and what was reportedq27_different_stages_6m

58) While your baby lying on his back, does he put his feet in his mouth?
q28_foot_mouth_stages_6m

1. Yes
2. Sometimes
0. No

59) Observed/reported
1. Observedq28_observed_stages_6m
2. Reportedq28_reported_stages_6m
3. There is difference between observation result and what was reportedq28_different_stages_6m

60) Does your baby try to get a toy that out of reach? (he may roll, pivot on his tummy, or crawl to get it)
q29_get_far_toy_stages_6m

1. Yes
2. Sometimes
0. No

61) Observed/reported
1. Observedq29_observed_stages_6m
2. Reportedq29_reported_stages_6m
3. There is difference between observation result and what was reportedq29_different_stages_6m

62) Does your baby use both hands and both legs equally well?if no, explain:
use_both_arms_well_stages_6m

63) When you help your baby stand, are his feet flat on the surface most of the time? if no, explain:
feet_flat_stages_6m

64) Do you have concerns that your baby is too quite or does not make sounds like other babies?if yes, explain:
worry_quiet_stages_6m

65) Does either parent have a family history of childhood deafness or hearing impairement? if yes, explain:
family_hearing_imp_stages_6m

66) Do you have concenrs about your baby's vision? if yes, explain:
worry_vision_stages_6m

67) Does your baby roll from his back to his tummy, getting both arms out from under him?
Roll_stages_6m

1. Yes
2. Sometimes
0. No

68) Observed/reported

1. Observedq30_observed_stages_6m
2. Reportedq30_reported_stages_6m
3. There is difference between observation result and what was reportedq30_different_stages_6m

PART 5 - HOME OBSERVATION

---

1) Interviewer:*
interviewer_house

Use your initial every time you have to fill in the interviewer question.

1. AD : Aly Diana
2. EI : Eva Indirawati
3. M : Monik
4. DEL : Dimas Erlangga Luftimas
5. R : Rima
6. H : Hendro

66. Lainnya

2) Infant's name:*
It is better to fill in the full name of infant. If it is not possible, you can fill in the forename, middle name or nick name. Please match the name that was told by mother with the name written in the paper.

3) Infant's ID number:*
id_house

It is no need of asking this question. It is written in the paper given to you.

HOUSE ENVIRONMENT

4) What is the main source of drinking water for your household?
water_house
When doing home observation, observe the main source of drinking water for her household.

1. Piped water into dwelling
2. Piped water into yard/plot
3. Piped water to public tap
4. Open well in dwelling
5. Open well in yard/plot
6. Open public well
7. Protected well in dwelling
8. Protected well in yard/plot
9. Protected public well
10. Water from spring
11. Water from rivers/stream
12. Pond/lake
13. Rain water
14. Bottled water
15. Ask from families or neighbors

66. Other

99. Missing

5) What kind of roads are by the house?
road_house

The important thing is whether the road is paved or unpaved

1. Paved
2. Non-paved

6) What are the walls of the house made of?
wall_house

Observe the materials of the walls. Fill in the question.

1. Concrete/brick
2. Wood
3. Bamboo
66. Other wall_othr_house

7) What is the material of the floor of the house?
floor_house

Observe the materials of floor of the house. Fill in the question.

1. Earth or dirt
2. Bamboo
3. Wood
4. Concrete/brick
5. Marble/granite/ceramic
6. Cement
66. Other

8) What is the construction material of the roof of the house? (If you are not sure, please ask the respondents. If you are still unsure, please answer Don't know)
roof_house

Observe the materials of roof of the house. Fill in the question. If you are not sure, plaease ask the respondents. If you are still unsure, please answer Don't know

1. Brick/Concrete
2. Wood
3. Asbestos/Zinc
4. Leaves
5. Plasterboard
6. Bamboo
98. Don't know
66. Otherroof_othr_house

9) Is there enough light inside the house during daylight?
light_house

Observe the light inside the house during interview.

1. Yes
0. No
99. Missing

10) Are there any ventilation/windows in the house which can support good air change?
ventilation_house

Observe any ventilation/windows in the houseduring interview.

1. Yes
0. No
99. Missing

11) Are the ventilation/windows opened during the visit?
open_ventilation_house

Observe whether the ventilation/windows are opened or not during the visit.

1. Yes
0. No
99. Missing

12) Are there any mechanical equipment to circulate/move the air inside the house (for example: fan)?
fan_house

Observe whether there any mechanical equipment to circulate/move the air inside the house or not

1. Yes
0. No
99. Missing

---

13) Is there a bednet over the infant's bed?
bednet_house

You have to observe if there is the bed net over the infant's bed.

1. Yes
0. No
99. Missing

PART 6 – ANTHROPOMETRIC MEASUREMENT

1) Interviewer:*
Use your initial every time you have to fill in the interviewer question.

1AD : Aly Diana
2EI : Eva Indirawati
3M : Monik
4DEL : Dimas Erlangga Luftimas
66

2) Date of interview (dd/mm/yyyy):*
date_exam_meas_6m
Use format day/month/year to fill in this interviewer question

3) Infant's name:*
baby_meas_6m
It is better to fill in the full name of infant. If it is not possible, you can fill in the forename, middle name or nick name. Please match the name that was told by mother with the name written in the paper.

4) Infant's ID number:*
id_meas_6m
It is no need of asking this question. It is written in the paper given to you.

5) Birth weight (gr) (look at the health card):
Ask mother about infant's birth weight in gram. Also, ask mother to show you the health card. Infant's birth weight usually is written in the card.

INFANT WEIGHT (questions 6,7,8)

weight_1_meas_6m

weight_2_meas_6m

weight_3_meas_6m

ave_weight_meas_6m

A pediatric scale should be used with a scale pan large enough to support the weight of the child. The child should be undressed and naked. Record the weight to the nearest 10 g. Repeat the measurement. If the difference between the two measurements is >0.1 kg, then take a third measurement. Record the measurements on the form. Take the mean of the two closest measurements. Write in gram.

INFANT LENGTH (questions 9,10,11)

length_1_meas_6m

length_2_meas_6m

length_3_meas_6m

ave_length_meas_6m

For all the infants in the Indonesian study, recumbent length will be measured with a measuring board. It is best to take this length measurement immediately after the weight so that the infant is dressed only in a nappy. Two examiners are required to correctly position the infant and to ensure accurate and reliable measurements of length. Record the two measurements on the form. Take a third measurement if the difference between the first two measurements is > 0,5 cm. Write in cm

MID-UPPER ARM CIRCUMFERENCE (questions 12,13,14)

muac_1_meas_6m

muac_2_meas_6m

muac_3_meas_6m

Obtain this measurement with the mother or caregiver seated and holding the infant in her lap. The infant should be wearing loose clothing without sleeves to allow exposure of the shoulder area. Measurements are taken to the nearest mm. Repeat the measurement. Take a third measurement if the difference between the first two measurements is >5 mm (0.5 cm).

HEAD CIRCUMFERENCE (questions 15,16,17)

hc_1_meas_6m

hc_2_meas_6m

hc_3_meas_6m

For the measurement, a fiberglass insertion tape, or a narrow, flexible, nonstretch tape made of fiberglass about 0.6 cm wide should be used. Obtain this measurement with the mother or caregiver seated and holding the infant in her lap. Measure the circumference to the nearest millimeter in duplicate. Repeat the measurement. Take a third measurement if difference between two measurements is > 0.5 cm. write in cm.

MOTHER HEIGHT (questions 18,19,20)

height_1_meas_6m

height_2_meas_6m

height_3_meas_6m

Height is measured using a stadiometer, or portable anthropometer. Clothing should be minimal when measuring height so that posture can be clearly seen. If possible, braids should be removed if they interfere with the

height measurement. Shoes and socks should not be worn. Record the time of day and the height to the nearest millimeter on the anthropometric form. Repeat the measurement. Take a third measurement if the difference between the first two measurements is > 0.7 cm. In cases where large amounts of adipose tissue prevent the heels, buttocks, and shoulders from simultaneously touching the wall, subjects should simply be asked to stand erect. write in cm.

FATHER HEIGHT (questions 21,22,23)

Height is measured using a stadiometer, or portable anthropometer. Clothing should be minimal when measuring height so that posture can be clearly seen. If possible, braids should be removed if they interfere with the height measurement. Shoes and socks should not be worn. Record the time of day and the height to the nearest millimeter on the anthropometric form. Repeat the measurement. Take a third measurement if the difference between the first two measurements is > 0.7 cm. In cases where large amounts of adipose tissue prevent the heels, buttocks, and shoulders from simultaneously touching the wall, subjects should simply be asked to stand erect. write in cm.

24) Reported father's height (cm)

Ask the mother to specify **father's height (cm).**

MOTHER WEIGHT (questions 25,26, 27)

wght_mom_1_meas_6m

wght_mom_2_meas_6m

wght_mom_3_meas_6m

Use an electronic balance for the measurement of weight in adults. The balance should be calibrated regularly and whenever it is moved to another location. Measurements should preferably be made after the bladder has been emptied, and before a meal. Record the body weight to the nearest 0.1 kg on the anthropometric form.Repeat the measurement. Take a third measurement if the difference between the first two measurements is > 0.1 kg.

MOTHER HEMOGLOBIN (g/dL) (question 28):

hb_meas_6m

Fingerprick blood samples will be done to analyzehaemoglobin of mother. Hb analyze will be done using HemoCue™ Hb 201. It will be done not in the time of visiting, but on Thursday, in the same day of taking infant's blood.

29) Has the Mother's MMA been done?
The important thing is whether Mother's MMA has been doneor not.

1

0

QUESTIONNAIRES 9 MONTH

Adequacy of Micronutrient Intakes and Status of

Breastfed Indonesian Infants

Fed Traditional Complementary Foods

Sumedang District

2014 - 2015

26) *Interviewer:**
interviewer_quest_9m
Interviewer is a field doctor who has been standardised.
Use your initial every time you have to fill in the interviewer question.
1. AD : Aly Diana
2. DEL : Dimas Erlangga Luftimas
3. M : Monik
4. R : Rima
5. HF : Hana Fauzia
6. NN : Ninda
7. TF : Tri Mulya Fitriasari
8. S : Sofa Rahmannia
9. HK : Hendro Kasmanto

66. : Other

27) *Date of interview (dd/mm/yyyy):**
date2_quest_9m
Use format day/month/year to fill in this interviewer question.
Every time you have to fill in the date, please use dd/mm/yyyy format.

28) *Mother's name:**
mom_quest_9m
*It is better to fill in the full name of mother. If it is not possible, you can fill in the forename, middle name or nick name. Please match the name that was told by mother with the name written in the paper**.*

29) *Mother's ID number:**
mom id_quest_9m
*It is no need of asking this question. It is written in the paper** given to you. Please re-check once again whether you have typed the ID number correctly.*

30) *Has the interviewer asked the mother to record the conversation that will be done? Has the interviewer prepared the recorder?*
record_quest_9m
Please remember that the conversations or the questioning process must be recorded as a back-up data, in case the off-line survey (SurveyGizmo) encountered any unwanted problem. The only answer of these questions is 1. Please make sure that the recorder functioned optimally during each visit.
Options:
1. Yes
2. Yes, but mother doesn't allow

31) *Does your house have a garden or agricultural land?*
garden_quest_9m
The important thing in this question is whether she has a garden or agricultural land. It is being asked to assess whether any vegetables or spices or herbs that the household consumed come from the garden or not.
1. Yes

0. No

7) ***Is any food for the household grown in the garden or agricultural land?***
eat_quest_9m
You have to ask if there is any food for the household grown in the garden/agricultural land or not.
1. Yes
0. No

8) ***If the answer is 1, please specify the type of food:***
plant_quest_9m
You have to specify the answer from mother about any food that she got from her garden. Please write the list of all food which come from her own garden/agricultural land.

Yesterday during the day or night (for questions 9-15):
These questions are asked to check the dietary diversity among the household. Please answer 1, if ANY members of the household consume the specified food. The number of the food is not of concern. Read the food lists. Underline the corresponding foods consumed and tick the column 1 or No depending on whether any food item of the list was consumed.

9) ***Did your household eat grains, roots and tubers?***

	Code	1. Yes	0. No	99. Missing
Porridge, bread, rice, maize, noodles or other foods made from grains	ricehh_quest_9m			
White potatoes, white yams, cassava or any other foods made from roots	roothh_quest_9m			

10) ***Did your household eat any foods made from beans (kidney beans, mung beans, soy beans, tofu, tempeh), peas, lentils, nuts (peanuts) or seeds?***
nutshh_ quest _9m
1. Yes
0. No

11) ***Did your household consume any dairy products?***

	Code	1 Yes	0 No	99. Missing
Milk, such as tinned, powdered or fresh animal milk	milkhh_quest _9m			

Yogurt or drinking yogurt	yoghurthh_quest_9m			
Cheese or other dairy products	cheesehh_quest _9m			

12) Did your household eat any flesh foods?

	Code	1 Yes	0 No	99. Missing
Liver, kidney, heart or other organ meats	organhh_ quest _9m			
Any meat, such as beef, pork, lamb, goat, chicken or duck	fleshhh_ quest _9m			
Fresh or dried fish, shellfish or seafood	seafoodhh_ quest _9m			

13) Did your household eat chicken eggs, duck eggs or quail eggs?

egghh_quest_9m

1. Yes
0. No

14) Did your household eat yellow/red/orange fruits and/or dark green leafy vegetables?

	Code	1. Yes	0. No	99. Missing
Pumpkin, carrots, tomatoes, squash or sweet potatoes that are yellow or orange inside	yellowhh_quest _9m			
Any dark green leafy vegetables, e.g. spinach, cassava leaves, water spinach, broccoli, caweightage, lettuce	greenhh_ quest _9m			
Ripe mangoes (fresh or dried), ripe papayas	orangehh_quest _9m			

15) Did your household eat any other foods mentioned below?

Ask the mother whether household eat or not any food in classification, such as oils fat classification, sugary food classification, any sweet beverage classification, any coffee and/or tea, and any condiments classification.

	Code	1 Yes	0 No	99. Missing
Any oils, fats, or butter or foods made with any of these	oilhh_ quest _9m			
Any surgary foods, such as chocolates, sweets, candies, pastries, cake or biscuits	sugarhh_ quest _9m			
Any sweet beverage (soda, fruits flavoured drink)	beveragehh_quest_9m			
Any coffee and/or tea	coffeehh_ quest _9m			
Condiments for flavour, such as chilies, spices, herbs, soy sauce, ketchup, or fish powder	spicehh_ quest _9m			

16) ***Does this household own any farm animals, e.g. milk cows, cattle, fish, poultry?***
animalshh_ quest _9m
Ask the mother whether her household own any farm animals or not. It is being asked to assess whether any animal protein that the household consumed come from the farm or not. In addition, it will also reflect the economic condition of the household.
1. Yes
0. No

17) ***If 1, please specify (type and number):***
animalshh_type_ quest _9m
Ask the mother to specify the type of animals, and how many animals that she has.

18) ***Does your household eat meat, poultry, fish or eggs [produced in your farm]?***
eathh_ quest _9m
You have to ask the mother whether household eat meat, poultry, fish or eggs produced in her farm.
1. Yes
0. No
97.NA

19) ***Number of meals per day your household usually eats (excluding snacks):***
mealhh_ quest _9m
Please specify the number of meals per day her household usually eats, excluding snacks. Household eating schedule often related to infant's eating schedule.

1. 1
2. 2
3. 3
4. 4
5. 5+
99.Missing

20) ***Number of snacks (made or bought) per day your household usually eats:***
snackhh_ quest _9m
Please specify the numberof snacks (made or bought) per day her household usually eats. Remember this question as only for the number of snacks.
1. 1
2. 2
3. 3
4. 4
5. 5+
99.Missing

21) ***Has your household received any food assistance (e.g. from the government, neighbors, relatives) in the last 6 months?***
ricehh_ quest _9m
The important thing is whether her household received or not any food assistance in the last 6 months. It shows the economic level of household.
1. Yes
0. No

22) ***Where has this week's food mainly come from? (only one answer is allowed)***
source_ quest _9m
Ask the mother whereweek's food mainly come from.Please choose only one answer. If the mother told you several food source, encourage her to choose only one source listed in the question. If the answer does not listed, write the answer in 'other' option.
1. Garden
2. Purchased
3. Both garden and purchased
4. Wages in kind after working
5. Eating wild foods not traditionally used in daily diet
6. Food gifts from relief organisations, churches, government
66.Other

23) ***Has your infant been sick in the last 2 weeks?***
sick_quest_9m
*This question means whether the **infant has been sick in the last 2 weeks or not or don't know.***
1. Yes
0. No
98.Don't know
99.Missing

24) ***Has your infant been vomiting in the last 2 weeks?***
vomit_quest_9m

The important thing is whether the infant has been vomiting in the last 2 weeks or not. Please choose '1' or 'no'. If mother work outside house and she doesn't know whether the infant vomit or not, please choose 'don't know'.

1. Yes
0. No
98.Don't know
99.Missing

25) *Has your infant been ill with a fever at any time in the last 2 weeks?*

fever_quest_9m

Ask the mother whether the infant has been ill or not with a fever at any time in the last 2 weeks. Underlying disease can cause any change in nutritional status.

1. Yes
0. No
98.Don't know
99.Missing

26) *Has your child had diarrhea in the last 2 weeks?*

diarrhea_quest_9m

The purpose of this question is to know whether the infant had diarrhea in the last 2 weeks or not.

1. Yes
0. No
98.Don't know
99.Missing

If No, then go to question number 31.

27) *If 1, how many sachets of Oralit (ORS) had been given to your infant during the whole episode of diarrhea?*

ors_quest_9m

The important thing of this question is how many sachet oralithad been given to the infant as oral rehydration solution during the whole episode of diarrhea (total number).

1. 0
2. 1-3
3. 4-5
4. 6+
97.NA

28) *How many glass (ml) of ORS had been given to your infant every time he/she defecate?*

ors_glass_quest_9m

The purpose of this question is to know the number of glass (ml) of ORS had been given to the infant every time he/she defecate.

1. 0
2. <50 ml
3. 50 ml (1/4 glass)
4. 100 ml (1/2 glass)
5. 150 ml (3/4 glass)

6. 200 ml (1 glass)
7. >200 ml
97. NA
98. Don't know

29) How many days was your infant given zinc supplements?
zinc_day_quest_9m
This question means duration that the infant consumed zinc supplements in days.
1. 0
2. 1-3
3. 4-6
4. 7-9
5. 10+
97.NA

30) How many tablet(s)/day was your infant given zinc supplements?
zinc_quest_9m
The important thing is the number of zinc supplements tablet consumed by the infant in a day.
1. ½
2. 1
3. 2
4. 3+
97.NA
98.Don't know

31) Has your child had an illness with a cough at any time in the last 2 weeks?
cough_quest_9m
This question means whether the infant had an illness with a cough at any time in the last 2 weeks or not.
1. Yes
0. No
98.Don't know
99.Missing

If No, then go to question number 33.

32) When your child had an illness with a cough in the last 2 weeks, did he/she breathe faster than usual with short, rapid breaths or have difficulty breathing?
apnea_quest_9m
The important thing is when the infant had an illness with a cough in the last 2 weeks, whether she breathe faster than usual with short, rapid breaths or have difficulty breathing or not. The purpose of this question is to determine the possibility of pneumonia cases.
1. Yes
0. No
97.NA
98.Don't know
99.Misssing

33) Has your infant had dengue fever in the last 3 months?

dengue_quest_9m

*This question means whether **infant has** had dengue fever **or not or don't know in the last 3 months.***

1. Yes
0. No
98.Don't know
99.Missing

34) Has your child been feeding poorly or refusing food in the last 2 weeks?

refuse_eat_quest_9m

*This question means whether **infant has** been feeding poorly or refusing food in the last 2 weeks **or not or don't know.***

1. Yes
0. No
98.Don't know
99.Missing

35) Has your infant been given worm medicine in the last 3 months?

wormmed_quest_9m

The important thing is whether the infant has been given worm medicine in the last 3 months or not. The consumption of worm medicine can eradicate the worms in infant gut.

1. Yes
0. No
98.Don't know
99.Missing

36) Has your infant been sick since born?

ever_sick_quest_9m

*This question means whether **infant has** been sick since born **or not or don't know.***

1. Yes
0. No
99.Missing

If No, then go to question number 39.

37) Did you seek advice for any of the illnesses from health workers when (NAME) got sick in the last 3 months?

seek_health_quest_9m

This question means whether mother seek advice for any of the illnesses from health workers or not.

1. Yes
0. No
97.NA
98.Don't know
99.Missing

If No, then go to question number 39.

38) If 1, which health workers you visited (most often)in the last 3 months?

health_quest_9m

The purpose of this question is to know which health workers that mother visited (most often) in the last 3 months. Please choose one. (NAME refers to child's name)

1. Doctor
2. Nurse
3. Midwife
4. Cadre

97.NA

99.Missing

66.Other

39) Currently, has (NAME) received any medications?

medicine_quest_9m

The important thing is whether (NAME) currently received any medications or not.

1. Yes

0. No

99.Missing

If No, then go to question number 41.

40) Could you please show the medication (if you still keeping the blister/package/bottle)?

medicine_show_quest_9m

Whether it is yes/No, please specify (name, dose, days of administration) of all medications received:

Ask the mother to specify the name, dose, days of administration of all medications received. Ask mother to show the medication if she still keeping the blister/package/bottle. The purpose of showing the blister/package/bottle is to make sure the drug consumed by the infant. We can also know the dosage given to infant precisely.

41) Has (NAME) received any drug in the last 1 month?

drug_onemonth_quest_9m

This question means whether (NAME) has received any drug in the last 1 month ***or not or don't know.***

1. Yes

0. No

97.NA

99.Missing

If No, then go to question number 44

42) Has (NAME) received any antibiotics in the last 1 month?

ab_1m_quest_9m

Ask the mother whether (NAME) received or not any antibiotics in the last 1 month. Antibiotic consumption can influence gut micro bacteria examined in infant.

1. Yes

0. No
97.NA
98.Don't Know
99.Missing

43) ***Has (NAME) received any antifungal in the last 1 month?***
af_quest_9m
Ask the mother whether (NAME) received or not any antifungal in the last 1 month. Antifungal consumption can influence gut parasite examined in infant.
1. Yes
0. No
97.NA
98.Don't Know
99.Missing

44) ***When is the last time (NAME) received antibiotics?***
ab_quest_9m
Ask the mother when the the last time (NAME) received antibiotics. You can fill in this question in date format or using adverb of time such as 2 weeks ago.

45) ***What is the name of the last antibiotics?***
ab_ name_quest _9m
Fill in this question with the name of the last antibiotics that (NAME) consumed since born.

46) ***How many days was the antibiotics given?***
ab_ day_quest _9m
Fill in this question with the duration of the last antibiotics that (NAME) consumed since born.

47) ***When is the last time (NAME) received antifungal?***
af_ quest _9m

Ask the mother when the the last time (NAME) received antifungal. You can fill in this question in date format or using adverb of time such as 3 weeks ago.

48) ***What is the name of the antifungal?***
af_name_quest_9m
Fill in this question with the name of the last antibiotics that (NAME) consumed since born.

49) ***How many days was the antifungal given?***
af_ day_quest _9m
Fill in this question with the duration of the last antifungal that (NAME) consumed since born.

50) ***How did you give breastfeeds and complementary food during the time your baby was sick in the last 3 months (in general)?***
cf_sick_quest_9m
The important thing is mother give the same number of breastfeeds and the same amount of complementary food during the time her baby was sick or not.
1. More than usual

2. Less than usual
3. Same as usual
97.NA
99.Missing

51) If 1, please specify (type and number):
cf_type_ quest _9m

52) Do you withhold certain food from your infant when (NAME) is ill in the last 3 months (in general)?
cf_limit_quest_9m
Fill in this question whether mother withhold certain food from her infant when (NAME) is ill (in general) or not.
1. Yes
0. No
97.NA
98.Don't Know
99.Missing

53) If yes, please specify:
cf_limit_name_quest_9m
Ask the mother to specify what the certain food is. Try to get the detail information.

54) Do you give any special foods to your infant after illness in the last 3 months (in general)?
cf_after_sick_quest_9m

Fill in this question whether mother give special foods to her after illness (in general) or not.
1. Yes
0. No
97.NA
98.Don't Know
99.Missing

55) If 1, please specify:
cf_as_name_quest_9m

Ask the mother to specify what the special food is. Try to get the detail information.

56) Has your infant been hospitalized in the last 3 months?
hospital_ever_quest_9m
The important thing is whether infant has been hospitalised or not or don't know.
1. Yes
0. No
98.Don't Know
99.Missing

If No, then go to question number 59

57) ***If 1, how many times has your infant been hospitalised?***
hospital_number_quest_9m
Ask the mother to specifyhow many times her infant has been hospitalised or don't know.
1. 1
2. 2
3. 3+
98.Don't know
99.Missing

58) ***Please specify the reason(s) for hospitalisation:***
hospital_reason_quest_9m
Ask the mother to specifythe reason(s) for hospitalisation.

59) ***Note infant birthweight (gram) as written in the KMS :***
birthweight_KMS_quest_9m

____

60) ***Has your infant been immunised?***
immunisation_quest_9m
The important thing is whether the infant has been immunized or not. It is very important that you ask each question exactly as it is written on the questionnaire. Even though the mother said only once, choose '1' choice button.
1. Yes
0. No
99.Missing

61) ***Can mother show the infant's immunisation card?***
immunisation_card_quest_9m
Ask the mother whether mother can show the infant's immunisation card or not. Be sure that she understands that the "card" refers to vaccination documentation. In some cases the mother may not be willing to take time to look for the vaccination card, thinking that you are in a hurry. Encourage her to look for (NAME's) card. It is highly desirable to obtain written documentation of the child's immunization history; therefore, be patient if the respondent needs to search for the card.
1. Yes
0. No
99.Missing

If 1, please TAKE PICTURE of the IMMUNISATION CARD, and fill these questions based on the information in IMMUNISATION CARD! If NOT, Ask the mother when the interviewer can go back and see thatIMMUNISATION CARD. If there is not card, fill DONT KNOW for questions number 55-59.

62) ***Tuberculosis vaccination (BCG)?***
bcg_quest_9m

See in the card whether the infant has received tuberculosis vaccination (BCG) or not.

1. Yes
0. No
97. NA
98. Don't know
99. Missing

63) Polio vaccination?

polio_quest_9m

See in the card whether the infant has received Polio vaccination or not.

1. Yes
0. No
97. NA
98. Don't know
99. Missing

64) Diptheria, Pertussis and Tetanus (DPT) vaccination?

dpt_quest_9m

See in the card whether the infant has received Diptheria, Pertussis and Tetanus (DPT)or not.

1.Yes
0.No
97.NA
98.Don't know
99.Missing

65) Measles vaccination?

measles_quest_9m

See in the card whether the infant has received Measles vaccination or not.

1. Yes
0. No
97. NA
98. Don't know
99. Missing

66) Hepatitis B vaccinations?

hep_b_quest_9m

See in the card whether the infant has received Hepatitis B vaccinations or not.

1. Yes
0. No
97. NA
98. Don't know
99. Missing

67) At any time in the past 3 months, has anyone come into your house to spray the inside walls against mosquitoes?

fogging_quest_9m

Fill in the question whether the house has been fogged inside walls against mosquitoes or not. If mother doesn't know, choose 'don't know'.

1. Yes

0. No
98.Don't know
99.Missing

68) ***Does your household have any insecticide treated mosquito nets that can be used while sleeping?***
bednet_ quest _9m
Fill in the question whether household have any insecticide treated mosquito nets that can be used while sleeping or not.
1. Yes
0. No
98. Don't know
99. Missing

If No, then go to question number 70

69) ***If 1, did your infant sleep under an insecticide treated mosquito net last night?***
bednet_child_quest_9m
The important of this question is whether the infant sleep under an insecticide treated mosquito net last night or not.
1. Yes
0. No
97. NA
98. Don't know
99. Missing

70) ***Who usually cooks your infant's food in the last 3 months?***
cook_ quest_9m

Ask the mother who usually cooks the infant's food to fill in this question. Remember to choose only one.
1. Myself (mother)
2. Mother in law
3. Mother
4. Caregiver
5. Sibling
66. Other

71) ***Who usually feeds your infant in the last 3 months?:***
feed_quest_9m

Ask the mother who usually feeds her infant. Please choose one answer.
1. Myself (mother)
2. Mother in law
3. Mother
4. Caregiver
5. Sibling
66. Other

72) ***Do you always cover food until it is eaten?***
food_cover_ quest _9m

Fill in the question whether mother always cover food until it is eaten or not.

1. Yes
0. No
99. Missing

73) There are key moments when you need to wash your hands to prevent germs from reaching food. What are these key moments?

In this question you can choose more than one answer. Fill in the key moments when mother need to wash her hands to prevent germs from reaching food.

1. After going to the toilet/latrine wash_toilet_ quest _9m
2. After cleaning the baby's bottom/changing a baby's nappy wash_diaper_ quest _9m
3. Before preparing/handling food wash_cook_ quest _9m
4. Before feeding a child wash_feed_ quest _9m
5. Before eating wash_eat_ quest _9m
6. After handling raw food wash_raw_ quest _9m
7. After handling garbage wash_trash_ quest _9m
98. Don't know wash_dk_ quest _9m
66. Other wash_othr_ quest _9m

74) Did (NAME) drink anything from a bottle with a nipple yesterday or last night?

bottle_quest_9m

Ask mother whether the infant or child drank anything from a bottle with a nipple yesterday. Baby bottles are not recommended, because they are very difficult to clean. Spoons, cups, and gourds may also be dirty, but bottles are even more likely to be contaminated and to make the baby sick. Fill in this question whether (NAME) drank anything from a bottle with a nipple yesterday or last night or not.

1. Yes
0. No
98. Don't know
99. Missing

75) Was (NAME) breastfed of did he/she consume breastmilk during the day or at night?

yest_breast_9m

Fill in this question whether the infant was breastfed during all day or not.

1. Yes
0. No
98. Don't know
99. Missing

If 1, continue to question 78

76) If No, for how long did you breastfeed (NAME)? (in months)

long_quest_9m

Ask the mother how long she breastfeed (NAME) in month.

77) Why did you stop breastfeeding?

stop_quest_9m

The important thing is the reason why the mother stopped breastfeeding. Then fill in this question.

1. Mother pregnant
2. Mother unwell
3. Mother tired of breastfeeding
4. Introduced solids
5. Breast milk making child sick
6. Not enough milk
7. Infant refused breast milk
8. Formula milk is better

66. Other
97. NA
98. Don't know
99. Missing

Now I would like to ask you about other foods your child ate over the last 24 hours (question 78-87). I am interested in whether your child had the item even if it was combined with other foods. Yesterday, did your child eat:

78) Grains, roots and tubers?

Some foods in the list are listed as a single item – for example, white potatoes – but may usually be eaten in a soup or stew. If the infant or child has eaten a mixed food like a soup,or stew, record all the food groups in the mixed food. For example, if the child ate a stew ofpotatoes, maize, there should be a check mark for each of the two food groups that contain these foods. Do not check off foods that have been added in very small amounts, or for seasoning.

	Code	1 Yes	0 No	99 Missing
Porridge, bread, rice, maize, noodles or other foods made from grains	rice24_quest _9m			
White potatoes, white yams, cassava or any other foods made from roots	root24_ quest _9m			

79) Any foods made from beans (kidney beans, mung beans, soy beans, tofu, tempeh), peas, lentils, nuts (peanuts) or seeds?

nuts24_quest_9m

The important thing is whether infant eat any foods made from beans (kidney beans, mung beans, soy beans, tofu, tempeh), peas, lentils, nuts (peanuts) or seeds or not. Do not check off foods that have been added in very small amounts, or for seasoning.

1. Yes
0. No
99. Missing

80) Any dairy products?

Some foods in the list are listed as a single item. If the infant or child has eaten a mixed food record all the food groups in the mixed food. Please mark '1' or 'no' button

		1 Yes	0 No	99 Missing
Milk, such as tinned, powdered or fresh animal milk	milk24_quest_9m			
Formula milk	formula24_quest_9m			
Yogurt or drinking yogurt	yoghurt24_quest_9m			
Cheese or other dairy products	cheese24_quest_9m			

81) Any flesh foods?

Some foods in the list are listed as a single item. If the infant or child has eaten a mixed food record all the food groups in the mixed food. Please mark '1' or 'no' button. Do not check off foods that have been added in very small amounts, or for seasoning. For example, if a spoon of fish powder is added to a pot of stew, do not record that the infant or child has eaten fish

	Code	1 Yes	0 No	99 Missing
Liver, kidney, heart or other organ meats	organ24_ quest _9m			
Any meat, such as beef, pork, lamb, goat, chicken or duck	flesh24_ quest _9m			
Fresh or dried fish, shellfish or seafood	seafood24_ quest _9m			

82) Chicken eggs, duck eggs or qual eggs?

egg24_ quest _9m

The important thing is whether the infant eat chicken eggs, duck eggs or qual eggs or not.

1. Yes
0. No
99. Missing

83) Yellow/red/orange fruits and/or any dark green leafy vegetables?

Some foods in the list are listed as a single item – for example, pumpkin – but may usually be eaten in a soup or stew. If the infant or child has eaten a mixed food like a soup, or stew, record all the food groups in the mixed food. For

example, if the child ate a stew of pumpkin, papaya, and green leaves, there should be a check mark for each of the three food groups that contain these foods. Do not check off foods that have been added in very small amounts, or for seasoning. For example, If one fifth of tomato is included in the family pot, do not record that as an "other fruit or vegetable."

		1 Yes	0 No	99 Missing
Pumpkin, carrots, tomatoes, squash or sweet potatoes that are yellow or orange inside	yellow24_quest _9m			
Any dark green leafy vegetables, e.g. spinach, cassava leaves, water spinach, broccoli, caweightage, lettuce	green24_ quest _9m			
Ripe mangoes (fresh or dried), ripe papayas	orange24_ quest _9m			

84) Any other foods mentioned below?

Some foods in the list are listed as a single item – for example, biscuit – but may usually be eaten together such as buttered biscuit. If the infant or child has eaten a mixed food like buttered biscuit, record all the food groups in the mixed food. For example, if the child ate a buttered biscuit, there should be a check mark for each of the two food groups that contain these foods. Do not check off foods that have been added in very small amounts, or for seasoning. For example, If one chili pepper is included in the family pot, do not record that as an "other fruit or vegetable."

	Code	1 Yes	0 No	99 Missing
Any oils, fats, or butter or foods made with any of these	oil24_ quest _9m			
Any surgary foods, such as chocolates, sweets, candies, pastries, cake or biscuits	sugar24_ quest _9m			
Any sweet beverage, such as soda, fruit flavoured drink	beverage24_ quest _9m			
Any tea and/or coffee	coffee24_ quest _9m			
Condiments for flavour, such as chilies, spices, herbs, soy sauce, ketchup, or fish	spice24_ quest _9m			

powder				

85) How many times did (NAME) eat foods, that is meals and snacks other than liquids yesterday during the day or at night?

eat_yest_ quest _9m

This question asks about how many times the infant or child ate solid/semi-solid foods as meals and snacks 1terday. Ask the mother/caregiver the question just as it is written. Solid/semi-solid foods include family foods, and also many special dishes prepared for infants. Thick soups and stews should be included. Thick porridges are also included. Very thin, watery soups and gruels should not be included because infants and young children do not get enough energy (calories) from very thin soups and gruels. Liquids do not count for this question. Also, very small snacks, such as a bite or two of someone else's food, should not be counted.

1. 1
2. 2
3. 3
4. 4
5. 5
6. 6+

97. NA

86) How many meals (excluding snacks) did you feed your infant yesterday?

meal_ yest_quest _9m

This question asks about how many times the infant or child ate solid/semi-solid foods as meals 1terday. Ask the mother/caregiver the question just as it is written. Solid/semi-solid foods include family foods, and also many special dishes prepared for infants.

1. 1 meals/day
2. 2 meals/day
3. 3 meals/day
4. 4 meals/day
5. 5 meals/day
6. 6+ meals/day

97. NA
98. Don't know
99. Missing

87) Did your child eat any food prepared with flour yesterday during the day or night?

flour_yest_ quest _9m

This question asks whether infant ate or not any food prepared with flour that was fortified with micronutrients yesterday.

1. Yes, please write the brand:

0. No
97. NA
98. Don't know
99. Missing

88) Has your infant ever received salt fortified by iodine?

salt_quest_9m

This question asks whether infant ever received salt fortified by iodine or not. Iodine is important for children growth and intelligent. Iodine deficiency also can cause endemic goiter.

1. Yes
0. No
98. Don't know
99. Missing

89) *Please show the salt used in your household. Write down the brand:*

salt_name_ quest _9m

This question asks the mother to show the salt used in her household. Write down the brand.

90) *Has your infant ever received Vitamin A supplements in the last 3 months? (Please show vitamin A capsules)*

vit_a_ quest _9m

The purpose of question 90 is to find out whether or not the infant/child received a doseof vitamin A in the last 6 months. Lack of vitamin A causes children to get sick more easily andin extreme cases lack of vitamin A can cause eye damage and blindness. Vitamin A supplements are given because many children do not get enough vitamin A from the foods theyeat.

You will need examples of the vitamin A capsules or ampules that have been used in the project area. Show the mother/caregiver the capsule or ampule to help her remember if her infant or child ever received one.

1. Yes
0. No
98. Don't know
99. Missing

91) *Has your infant received multivitamin and minerals pills or syrup in the last month?*

multivitamin_ quest _9m

The purpose of question 28 is to find out whether or not the infant/child received a dose of multivitamin and minerals pills or syrup in the last month.

1. Yes
0. No
98. Don't know
99. Missing

92) *Has your infant received sprinkles (TABURIA) in the last 3 months? (Please show TABURIA)*

taburia_ quest _9m

This question asks whether infant has received sprinkles (TABURIA) in the last 6 months or not. (Please show TABURIA)

1. Yes
0. No
98. Don't know
99. Missing

If No or Don't Know , then go to question number 100

93) *From whom have you received the sprinkles (TABURIA)?*
taburia_whom_ quest _9m
The purpose of question 93 is to find out from whothe mother has received the sprinkles (TABURIA).
1. Puskesmas staff
2. Village Midwife
3. Cadres
66. Other
97. NA
99. Missing

94) *When did you receive it?*
taburia_when_ quest_9m
The purpose of question 94 is to find out the time that mother has received the sprinkles (TABURIA). TABURIA better been given at 6 month until 24 month old infant.
1. This month
2. Last month
3. Two months ago
4. Three months ago
5. Four months ago (or more)
97. NA
99. Missing

95) *How many sachets of sprinkles have you received in the last 3 months?*
taburia_number_ quest_9m
The purpose of question 95 is to find out how many sachets of sprinkles mother has received in the last 3 months
1. <15 sachets
2. 15 – 30 sachets
3. 30 – 60 sachets
4. >60 sachets
97. NA
98. Don't know
99. Missing

96) *How many sachets of sprinkles have you given to (NAME) in the last 3 months?*
taburia_eat_ quest _9m
The purpose of question 96 is to find out how many sachets of sprinkles mother give to (NAME) in the last 3 months.
1. <5 sachets
2. 5 – 10 sachets
3. 10 – 15 sachets
4. >15 sachets
97. NA
99. Missing

97) *How did you give the sprinkles to (NAME)?*
This question asks how mother give the sprinkles to (NAME). It should be given once every two days.

1. Twice a day taburia_twice_quest_9m
2. Every day taburia_once_quest_9m
3. Every other day taburia_days_quest_9m
4. Sprink it into infant porridge (cool) taburia_cold_quest_9m
5. Sprink it into infant porridge (hot) taburia_hot_quest_9m
6. Mix it with porridge/soup while cooking taburia_cook_quest_9m
7. Never taburia_never_quest_9m
97. NA taburia_na_quest_9m
66. Other othr6_quest_9m

98) How did (NAME) react to the addition of sprinkles (Taburia) to his/her food?

taburia_reaction_quest_9m

This question asks how (NAME) did react to the addition of sprinkles (Taburia) to his/her food.

1. Dislike very much
2. Dislike a little
3. Not sure
4. Like a little
5. Like very much
97. NA

99) Do you know what is the function/benefit of giving sprinkles to your infant? Please mention:

taburia_benefit_quest_9m

The purpose of question 99 is to find out the knowledge of mother about the function/benefit of giving sprinkles to infant.

---

100) Have you regularly brought your infant to Posyandu?

posyandu_quest_9m

The purpose of question 100 is to find out whether mother regularly brings her infant to Posyandu or not.

1. Yes, once per month
2. Yes, once every two month
3. Sometimes (1-2 times)
4. Never
98. Don't know

101) How many snacks did you feed your infant yesterday?

snack_yest_quest_9m

This question asks about how many times the infant or child ate solid/semi-solid foods as snacks yesterday. Ask the mother/caregiver the question just as it is written. Solid/semi-solid foods include family foods, and also many special dishes prepared for infants.

1. 1 snacks/day
2. 2 snacks/day
3. 3 snacks/day
4. 4 snacks/day
5. 5 +snacks/day
98. Don't know
97. NA

102) Do you give your children a separate plate?

plate_quest_9m

The important thing is whether mother gives her children a separate plate or not.

1. Yes
0. No
97. NA
98. Don't know
99. Missing

103) Did you add ingredients to your infant's porridge such as mashed vegetables, beans, fruits, eggs, and small fish?

porridge_add_quest_9m

The important thing is whether mother add ingredients to the infant's porridge such as mashed vegetables, beans, fruits, eggs, and small fish or not.

1. Yes
0. No
97. NA
98. Don't know
99. Missing

104) During the last two weeks did your infant eat any of the following foods? *(ALLOW MULTIPLE ANSWERS, READ THE LIST)*

The important thing is whether during the last two weeks infant eat any foods listed in the question. Encourage mother to multiple answers, but remember not to mention the foods listed.

The important thing is whether during the last two weeks infant eat any foods listed in the question. Encourage mother to multiple answers, but remember not to mention the foods listed.

1. Cassava leaves cassava_leaf_quest_9m
2. Pumpkin leaves pumpkin_leaf_quest_9m
3. Carrots carrot_quest_9m
4. Pumpkin pumpkin_quest_9m
5. Groundnuts nuts2_quest_9m
6. Potato leaves potato_leaf_quest_9m
7. Mangoes mango_quest_9m
8. Papaya papaya_quest_9m
9. Eggs egg3_quest_9m
10. Meat flesh2_quest_9m
11. Fish fish_quest_9m
12. Banana banana_quest_9m
13. Avocado avocado_quest_9m
14. Sweet potato sweet_potato_quest_9m
15. Animal milk milk3_quest_9m
98. Don't know dk_quest_9m
97. NA na_quest_9m

105) Are you the person who fed [NAME] yesterday?

mom_feed_quest_9m

The purpose of question 105 is to confirm whether mother was someone that fed the infant 1terday or not.

1. Yes
0. No
97. NA
99. Missing

106) *Yesterday, at the main meal, did [NAME] eat all the food you thought he/she should?*

eat_all_quest_9m

The purpose of question 106 is whether [NAME] eat all the food mother's thought he/she should or not.

1. Yes
0. No
97. NA
98. Don't know
99. Missing

107) *During this meal, were you or another caregiver sitting with your infant?*

sit_together_quest_9m

This question asks whether mother or another caregiver were sitting with infant during this meal or not.

1. Yes
0. No
97. NA
99. Missing

108) *If sitting with the infant, how were you two positioned?*

position_quest_9m

The question asks how about the position of mother/or caregiver to infant. Whether can see the infant's face or not.

1. Mother/caregiver positioned so she could NOT see infant's face
2. Mother/caregiver positioned so she could see infant's face frontways or sideways
97. NA
99. Missing

109) *How was the infant positioned?*

infant_position_quest_9m

The question asks how about the position of infant.

1. Infant held in mother or caregiver's arms
2. Infant lying in mother or caregiver's lap
3. Infant sitting in mother or caregiver's lap
4. Infant standing up or running around
5. Infant sitting on a chair, stool, mat or bed
97. NA
66. Other position_othr_quest_9m

110) Did your infant refuse to eat an offered mouthful of food?

refuse_quest_9m

This question asks whether infant refused to eat an offered mouthful of food or not.

1. Yes
0. No
97. NA
99. Missing

111) Did your infant cry during the meal yesterday?

cry__quest__9m

This question asks whether infant cried during the meal yesterday or not

1. Yes
0. No
97. NA
99. Missing

112) Did your infant try to leave the meal early yesterday?

leave_early_quest_9m

This question asks whether infant try to leave the meal early 1terday or not

1. Yes
0. No
97. NA
99. Missing

113) Did you have to control your infant while eating, such as by holding his/her head or body?

control_quest_9m

The purpose of question 113 is whether mother has to control your infant while eating, such as by holding his/her head or body.

1. Yes
0. No
97. NA
99. Missing

114) Yesterday at the evening meal, how was your infant behaviour?

behaviour_quest_9m

The purpose of question 114 is to find out infant behaviour on yesterday at the evening meal.

1. More fussy about eating than usual
2. Eating as she or he usually eats
3. Less fussy about eating than usual
98. Don't know
97. NA

115) Was your infant sick yesterday?

sick_yest_quest_9m

This question asks whether infant sick yesterday or not.

1. Yes

0. No
99. Missing

116) *Is your child able to feed themselves some of the meal?*
feed_themselves_quest_9m
This question asks whether infant able to feed themselves some of the meal or not
1. Yes
0. No
99. Missing

117) *If 1, at what age in months are children able to START feeding themselves some of the meal?*
feed_themself_age_quest_9m
The purpose of question 117 is to find out the specific age in months the children able to START feeding themselves some of the meal.

118) *Do you still often give porridge to [Name] ?*
porridges_still_quest_9m
1. Yes
0. No
99. Missing

119) *If 1, which type of porridge do you usually make for [Name]? Thick porridge or watery porridge?*
type_porridges_quest_9m
1. Thick porridge
2. Watery porridge
97. NA
98. Don't know
99. Missing

120) *Why do you choose that type of porridges?*
reason_quest_9m
The purpose of question 120 is to find out the reason why mother has chosen the type of porridge she made. .
1. Because the watery porridge is easier to be eaten.
2. Because the thick porridge is more nutritious or because it is prepared with different types of foods or ingridients (food diversity)
97. NA
98. Don't know
66. Other reason_othr_quest_9m

121) *To feed their children, many mothers give them rice porridge. Please tell me some ways to make rice porridge more nutritious or better for your baby's health.*
(Probe if necessary: Which foods or types of food can be added to rice porridge make it more nutritious?)
porridge_better_quest_9m

For this question, you have to ask the mother to explain some ways to make rice porridge more nutritious or better for her baby's health.

1. Animal-source foods (meat, poultry, fish, liver/organ meat, eggs, etc.)
2. Pulses and nuts: flours of groundnut and other legumes (peas, beans, lentils, etc.), sunflower seed, peanuts, soybeans
3. Vitamin-A-rich fruits and vegetables (carrot, orange-fleshed sweet potato, yelow pumpkin, mango, papaya, etc.)
4. Green leafy vegetables (e.g. spinach)
5. Energy-rich foods (e.g. oil, butter)

66. Other, please specify: ____
98. Don't know
99. Missing

AGES and STAGES 9 months

69) *Interviewer:**

interviewer_stages_9m

Use your initial every time you have to fill in the interviewer question.

1. AD : Aly Diana
2. DEL : Dimas Erlangga Luftimas
3. M : Monik
4. R : Rima
5. HF : Hana Fauzia
6. NN : Ninda
7. TF : Tri Mulya Fitriasari
8. S : Sofa Rahmannia
9. HK : Hendro Kasmanto

66. Other

70) *Date of interview (dd/mm/yyyy):**

date_stages_9m

Use format day/month/year to fill in this interviewer question.

71) *Mother's name:**

name_mom_stages_9m

*It is better to fill in the full name of mother. If it is not possible, you can fill in the forename, middle name or nick name. Please match the name that was told by mother with the name written in the paper**.*

72) *Mother's ID number:**

id_stages_9m

*It is no need of asking this question. It is written in the paper** given to you. Please re-check once again whether you have typed the ID number correctly.*

COMMUNICATION

73) *Does your infant make sounds like "da', "ga", "ka" and "ba"?*

q1_sound_da_9m

1 yes

2 sometimes

3 no

74) *Observed/ reported*

1 observed

q1_observed_9m

2 reported

q1_reported_9m

3 discrepancy between observation and report

q1_discrepancy_9m

75) If you imitate the sound came from your baby, does he/she imitating the sound of the sameback to you?
q2_imitating_sound_9m
1 yes
2 sometimes
3 no

76) Observed/ reported
1 observed
q2_observed_9m
2 reported
q2_reported_9m
3 discrepancy between observation and report
q2_discrepancy_9m

77) Has your baby been able to make two similar sounds such as"da-da" "ba-ba" "ga-ga" (the sound does not have to have a certain meaning)?
q3_similar_sound_9m
1 yes
2 sometimes
0 no

78) Observed/ reported
1 observed
q3_observed_9m
2 reported
q3_reported_9m
3 discrepancy between observation and report
q3_discrepancy_9m

79) If you ask he/she to do at least one motion of children's games, could she/he do that even if you do not provide examples (eg, 'bye', 'peekaboo', 'applause')?
q4_do_one_motion_9m
1 yes
2 sometimes
0 no

80) Observed/ reported
1 observed
q4_observed_9m
2 reported
q4_reported_9m
3 discrepancy between observation and report
q4_discrepancy_9m

81) Has your baby been able to follow simple commands, such as "come here", "Give it to me", "put it back" without you using gestures?
q5_simple_command_9m
1 yes
2 sometimes

0 no

82) Observed/ reported
1 observed
q5_observed_9m
2 reported
q5_reported_9m
3 discrepancy between observation and report
q5_discrepancy_9m

83) Has your baby been able to mention three words like "mama", "baba", "dada" (the word is the sound that your baby consistently said to shows someone or something)?
q6_three_words_9m
1 yes
2 sometimes
0 no

84) Observed/ reported
1 observed
q6_observed_9m
2 reported
q6_reported_9m
3 discrepancy between observation and report
q6_discrepancy_9m

GROSS MOTOR

85) If you hold both hands to balance your baby, is he/she could hold his weight when standing?
q7_hold_wieght_standing_9m
1 yes
2 sometimes
0 no

86) Observed/ reported
1 observed
q7_observed_9m
2 reported
q7_reported_9m
3 discrepancy between observation and report
q7_discrepancy_9m

87) While sitting on the floor, does your baby sit upright for a few minutes without using his hands to hold?
q8_upright_sitting_9m
1 yes
2 sometimes
0 no

88) ***Observed/ reported***
1 observed
q8_observed_9m
2 reported
q8_reported_9m
3 discrepancy between observation and report
q8_discrepancy_9m

89) ***When you put on your baby on standing position near the fence furniture or a baby crib, does he/she hold the furniture without his/her chest leaning on the crib/furniture?***
q9_hold_without_leaning_9m
1 yes
2 sometimes
0 no

90) ***Observed/ reported***
1 observed
q9_observed_9m
2 reported
q9_reported_9m
3 discrepancy between observation and report
q9_discrepancy_9m

91) ***When clinging to the furniture, does your baby bent his/her legs to take the toys off the floor and then stand back up?***
q10_bent_legs_9m
1 yes
2 sometimes
0 no

92) ***Observed/ reported***
1 observed
q10_observed_9m
2 reported
q10_reported_9m
3 discrepancy between observation and report
q10_discrepancy_9m

93) ***When clinging to the furniture, does your baby lower his/her own body in a controlled manner (without falling)?***
q11_lower_body_9m
1 yes
2 sometimes
0 no

94) ***Observed/ reported***
1 observed

q11_observed_9m
2 reported
q11_reported_9m
3 discrepancy between observation and report
q11_discrepancy_9m

95) ***Is your baby able to walk beside furniture with holding using just one hand?***
q12_walk_holding_one_hand_9m
1 yes
2 sometimes
0 no

96) ***Observed/ reported***
1 observed
q12_observed_9m
2 reported
q12_reported_9m
3 discrepancy between observation and report
q12_discrepancy_9m

FINE MOTOR

97) ***Does your baby hold small toys with only one hand?***
q13_toys_holding_one_hand_9m
1 yes
2 sometimes
0 no

98) ***Observed/ reported***
1 observed
q13_observed_9m
2 reported
q13_reported_9m
3 discrepancy between observation and report
q13_discrepancy_9m

99) ***Does your baby have managed to take the crumbs with the thumb and all the fingers with gestures like scratching? (if he has been taking crumbs, mark yes to this question)***
q14_take_crumbs_9m
1 yes
2 sometimes
0 no

100) ***Observed/ reported***
1 observed
q14_observed_9m
2 reported
q14_reported_9m
3 discrepancy between observation and report

q14_discrepancy_9m

101) Does your baby take a small toy with the tips of the thumb and fingers? (You should see the space / gap between toy with palms)

q15_tip_fingers_9m

1 yes
2 sometimes
0 no

102) Observed/ reported

1 observed
q15_observed_9m
2 reported
q15_reported_9m
3 discrepancy between observation and report
q15_discrepancy_9m

103) After trying one to two times, can your baby take a string with his/her thumb and forefinger (the rope may be attached to the toy)?

q16_tip_fingers_9m

1 yes
2 sometimes
0 no

104) Observed/ reported

1 observed
q16_observed_9m
2 reported
q16_reported_9m
3 discrepancy between observation and report
q16_discrepancy_9m

105) Has your baby been able to take the crumbs with the tip of the thumb and fingers? He/she might put his hands or arms on the table when doing it.

q17_tip_fingers_9m

1 yes
2 sometimes
0 no

106) Observed/ reported

1 observed
q17_observed_9m
2 reported
q17_reported_9m
3 discrepancy between observation and report
q17_discrepancy_9m

107) Has your baby been able to put his/her little toy down without dropping it and release the toy out of his hands?

q18_put_toy_down_9m

1 yes
2 sometimes
0 no

108) Observed/ reported
1 observed
q18_observed_9m
2 reported
q18_reported_9m
3 discrepancy between observation and report
q18_discrepancy_9m

PROBLEM SOLVING

109) Can your baby flipping the toy from one hand to the other hand?
q19_flipping_toy_9m
1 yes
2 sometimes
0 no

110) Observed/ reported
1 observed
q19_observed_9m
2 reported
q19_reported_9m
3 discrepancy between observation and report
q19_discrepancy_9m

111) Has your baby able to take two small toys, one toy in each hand, and grip it for a minute?
q20_take_and_grip_9m
1 yes
2 sometimes
0 no

112) Observed/ reported
1 observed
q20_observed_9m
2 reported
q20_reported_9m
3 discrepancy between observation and report
q20_discrepancy_9m

113) When holding a toy in his hand, does your baby slamming it against another toy on the table?
q21_holding_and_slamming_9m
1 yes
2 sometimes
0 no

114) Observed/ reported

1 observed

q21_observed_9m

2 reported

q21_reported_9m

3 discrepancy between observation and report

q21_discrepancy_9m

115) If your baby holding a small toy in each hand, does your baby clap the toys together? (Like clapping cakes)

q22_clap_toys_9m

1 yes

2 sometimes

0 no

116) Observed/ reported

1 observed

q22_observed_9m

2 reported

q22_reported_9m

3 discrepancy between observation and report

q22_discrepancy_9m

117) Can your baby stirring or trying to get crumbs inside a transparent bottle? (Like a plastic soda bottle or baby bottle)

q23_stirring_transparent_bottle_9m

1 yes

2 sometimes

0 no

118) Observed/ reported

1 observed

q23_observed_9m

2 reported

q23_reported_9m

3 discrepancy between observation and report

q23_discrepancy_9m

119) After seeing you hide a small toy under the paper or clothes, is your baby able find it? (Make sure the toy is completely hidden)

q24_find_hid_toys_9m

1 yes

2 sometimes

0 no

120) Observed/ reported

1 observed

q24_observed_9m

2 reported

q24_reported_9m

3 discrepancy between observation and report
q24_discrepancy_9m

PERSONAL-SOCIAL

121) When your baby is in a supine position, is he/she put his foot in his mouth?
q25_supine_foot_mouth_9m
1 yes
2 sometimes
0 no

122) Observed/ reported
1 observed
q25_observed_9m
2 reported
q25_reported_9m
3 discrepancy between observation and report
q25_discrepancy_9m

123) Does your baby drink water, juice, or formula in the glass when you hold it?
q26_drink_glass_9m
1 yes
2 sometimes
0 no

124) Observed/ reported
1 observed
q26_observed_9m
2 reported
q26_reported_9m
3 discrepancy between observation and report
q26_discrepancy_9m

125) Has your baby been able to eat his/her biscuits or cake by his/her own?
q27_eat_themselves_9m
1 yes
2 sometimes
0 no

126) Observed/ reported
1 observed
q27_observed_9m
2 reported
q27_reported_9m
3 discrepancy between observation and report
q27_discrepancy_9m

127) When you stretch out your hand and ask forhis/her toy, does your baby to give it to your hand although he/she doesn't want to let go? (If he/she is willing to give the toy to your hand give a"yes" to this question)

q28_willing_to_give_9m

1 yes
2 sometimes
0 no

128) Observed/ reported

1 observed
q28_observed_9m
2 reported
q28_reported_9m
3 discrepancy between observation and report
q28_discrepancy_9m

129) When you are dressing your baby, is he/she pushes his arm to past the sleeves when the arm has reached the hole sleeves?

q29_push_sleeves_9m

1 yes
2 sometimes
0 no

130) Observed/ reported

1 observed
q29_observed_9m
2 reported
q29_reported_9m
3 discrepancy between observation and report
q29_discrepancy_9m

131) When you stretch out your hand and ask for his/her toy, does your baby to give it to your hand?

q30_give_toys_9m

1 yes
2 sometimes
0 no

132) Observed/ reported

1 observed
q29_observed_9m
2 reported
q29_reported_9m
3 discrepancy between observation and report
q29_discrepancy_9m

GENERAL

Parents or related parties can fill in the fields below for more information.

133) Does your baby use both hands and feet with good and balanced? If not, please explain

134) When you help your baby to stand, do his feet more often tread? If not, please explain

135) Are you worried because your baby is too quiet or not making noises likeother babies? If yes, please explain:

136) Do parents have a family history of hearing loss or deafness? If yes, please explain:

137) *Are you worried about your baby's vision? If yes, please explain:*

138) Has your baby been experiencing medical problems in thelast few months? If yes, please explain:

139) Are you worrying your baby's behaviour? If yes, please explain:

140) Are there certain things you are worried about your baby? If yes, please explain:

QUESTIONNAIRES 12 MONTH

Adequacy of Micronutrient Intakes and Status of

Breastfed Indonesian Infants

Fed Traditional Complementary Foods

Sumedang District

2014 - 2015

32) Interviewer:

interviewer_quest_12m

Interviewer is a field doctor who has been standardised.

Use your initial every time you have to fill in the interviewer question.

1. AD : Aly Diana
2. DEL : Dimas Erlangga Luftimas
3. M : Monik
4. R : Rima
5. HF : Hana Fauzia
6. NN : Ninda
7. TF : Tri Mulya Fitriasari
8. S : Sofa Rahmannia
9. HK : Hendro Kasmanto

66\. : Other

33) Date of interview (dd/mm/yyyy):

date_quest_12m

Use format day/month/year to fill in this interviewer question.

Every time you have to fill in the date, please use dd/mm/yyyy format.

34) Mother's name:*

mom_quest_12m

It is better to fill in the full name of mother. If it is not possible, you can fill in the forename, middle name or nick name. Please match the name that was told by mother with the name written in the paper.*

35) Mother's ID number:*

mom id_quest_12m

It is no need of asking this question. It is written in the paper given to you. Please re-check once again whether you have typed the ID number correctly.*

36) Has the interviewer asked the mother to record the conversation that will be done? Has the interviewer prepared the recorder?

record_quest_12m

Please remember that the conversations or the questioning process must be recorded as a back-up data, in case the off-line survey (SurveyGizmo) encountered any unwanted problem. The only answer of these questions is 1. Please make sure that the recorder functioned optimally during each visit.

Options:

1. Yes
2. Yes, but mother doesn't allow

37) Does your house have a garden or agricultural land?

garden_quest_12m

The important thing in this question is whether she has a garden or agricultural land. It is being asked to assess whether any vegetables or spices or herbs that the household consumed come from the garden or not.

1\. Yes

0\. No

8) Is any food for the household grown in the garden or agricultural land?

eat_quest_12m

You have to ask if there is any food for the household grown in the garden/agricultural land or not.

1. Yes
0. No

122) If the answer is 1, please specify the type of food:

plant_quest_12m

You have to specify the answer from mother about any food that she got from her garden. Please write the list of all food which come from her own garden/agricultural land.

Yesterday during the day or night (for questions 9-15):

These questions are asked to check the dietary diversity among the household. Please answer 1, if ANY members of the household consume the specified food. The number of the food is not of concern. Read the food lists. Underline the corresponding foods consumed and tick the column 1 or No depending on whether any food item of the list was consumed.

123) Did your household eat grains, roots and tubers?

	Code	1. Yes	0. No	99. Missing
Porridge, bread, rice, maize, noodles or other foods made from grains	ricehh_quest_12m			
White potatoes, white yams, cassava or any other foods made from roots	roothh_quest_12m			

124) Did your household eat any foods made from beans (kidney beans, mung beans, soy beans, tofu, tempeh), peas, lentils, nuts (peanuts) or seeds?

nutshh_ quest _12m

1. Yes
0. No

125) Did your household consume any dairy products?

	Code	1.Yes	0.No	99. Missing
Milk, such as tinned, powdered or fresh animal milk	milkhh_quest _12m			

Yogurt or drinking yogurt	yoghurthh_quest_12m			
Cheese or other dairy products	cheesehh_quest _12m			

126) Did your household eat any flesh foods?

	Code	1 Yes	0 No	99. Missing
Liver, kidney, heart or other organ meats	organhh_ quest _12m			
Any meat, such as beef, pork, lamb, goat, chicken or duck	fleshhh_ quest _12m			
Fresh or dried fish, shellfish or seafood	seafoodhh_ quest _12m			

127) Did your household eat chicken eggs, duck eggs or quail eggs?

egghh_quest_12m

1. Yes
0. No

128) Did your household eat yellow/red/orange fruits and/or dark green leafy vegetables?

	Code	1. Yes	0. No	99. Missing
Pumpkin, carrots, tomatoes, squash or sweet potatoes that are yellow or orange inside	yellowhh_quest_12m			
Any dark green leafy vegetables, e.g. spinach, cassava leaves, water spinach, broccoli, caweightage, lettuce	greenhh_ quest_12m			
Ripe mangoes (fresh or dried), ripe papayas	orangehh_quest_12m			

129) Did your household eat any other foods mentioned below?

Ask the mother whether household eat or not any food in classification, such as oils fat classification, sugary food classification, any sweet beverage classification, any coffee and/or tea, and any condiments classification.

	Code	1 Yes	0 No	99. Missing
Any oils, fats, or butter or foods made with any of these	oilhh_ quest _12m			
Any surgary foods, such as chocolates, sweets, candies, pastries, cake or biscuits	sugarhh_ quest _12m			
Any sweet beverage (soda, fruits flavoured drink)	beveragehh_quest_12m			
Any coffee and/or tea	coffeehh_ quest _12m			
Condiments for flavour, such as chilies, spices, herbs, soy sauce, ketchup, or fish powder	spicehh_ quest _12m			

130) Does this household own any farm animals, e.g. milk cows, cattle, fish, poultry?

animalshh_ quest _12m

Ask the mother whether her household own any farm animals or not. It is being asked to assess whether any animal protein that the household consumed come from the farm or not. In addition, it will also reflect the economic condition of the household.

1. Yes
0. No

131) If 1, please specify (type and number):

animalshh_type_ quest _12m

Ask the mother to specify the type of animals, and how many animals that she has.

132) Does your household eat meat, poultry, fish or eggs [produced in your farm]?

eathh_ quest _12m

You have to ask the mother whether household eat meat, poultry, fish or eggs produced in her farm.

1. Yes
0. No
97.NA

133) Number of meals per day your household usually eats (excluding snacks):

mealhh_ quest _12m

Please specify the number of meals per day her household usually eats, excluding snacks. Household eating schedule often related to infant's eating schedule.

1. 1
2. 2
3. 3
4. 4
5. 5+

99.Missing

134) *Number of snacks (made or bought) per day your household usually eats:*

snackhh_ quest _12m

Please specify the numberof snacks (made or bought) per day her household usually eats. Remember this question as only for the number of snacks.

1. 1
2. 2
3. 3
4. 4
5. 5+

99.Missing

135) *Has your household received any food assistance (e.g. from the government, neighbors, relatives) in the last 6 months?*

ricehh_ quest _12m

The important thing is whether her household received or not any food assistance in the last 6 months. It shows the economic level of household.

1. Yes
0. No

136) *Where has this week's food mainly come from? (only one answer is allowed)*

source_ quest _12m

Ask the mother whereweek's food mainly come from.Please choose only one answer. If the mother told you several food source, encourage her to choose only one source listed in the question. If the answer does not listed, write the answer in 'other' option.

1. Garden
2. Purchased
3. Both garden and purchased
4. Wages in kind after working
5. Eating wild foods not traditionally used in daily diet
6. Food gifts from relief organisations, churches, government

66.Other

137) *Has your infant been sick in the last 2 weeks?*

sick_quest_12m

*This question means whether r**infant has been sick in the last 2 weeks or not or don't know.***

1. Yes
0. No

98.Don't know

99.Missing

138) Has your infant been vomiting in the last 2 weeks?

vomit_quest_12m

The important thing is whether the infant has been vomiting in the last 2 weeks or not. Please choose '1' or 'no'. If mother work outside house and she doesn't know whether the infant vomit or not, please choose 'don't know'.

1. Yes
0. No
98. Don't know
99. Missing

139) Has your infant been ill with a fever at any time in the last 2 weeks?

fever_quest_12m

Ask the mother whether the infant has been ill or not with a fever at any time in the last 2 weeks. Underlying disease can cause any change in nutritional status.

1. Yes
0. No
98. Don't know
99. Missing

140) Has your child had diarrhea in the last 2 weeks?

diarrhea_quest_12m

The purpose of this question is to know whether the infant had diarrhea in the last 2 weeks or not.

1. Yes
0. No
98. Don't know
99. Missing

If No, then go to question number 31.

141) If 1, how many sachets of Oralit (ORS) had been given to your infant during the whole episode of diarrhea?

ors_quest_12m

The important thing of this question is how many sachet oralithad been given to the infant as oral rehydration solution during the whole episode of diarrhea (total number).

1. 0
2. 1-3
3. 4-5
4. 6+
97. NA

142) How many glass (ml) of ORS had been given to your infant every time he/she defecate?

ors_glass_quest_12m

The purpose of this question is to know the number of glass (ml) of ORS had been given to the infant every time he/she defecate.

1. 0
2. <50 ml

3. 50 ml (1/4 glass)
4. 100 ml (1/2 glass)
5. 150 ml (3/4 glass)
6. 200 ml (1 glass)
7. >200 ml
97. NA
98. Don't know

143) How many days was your infant given zinc supplements?

zinc_day_quest_12m

This question means duration that the infant consumed zinc supplements in days.

1. 0
2. 1-3
3. 4-6
4. 7-9
5. 10+

97.NA

144) How many tablet(s)/day was your infant given zinc supplements?

zinc_quest_12m

The important thing is the number of zinc supplements tablet consumed by the infant in a day.

1. ½
2. 1
3. 2
4. 3+

97.NA
98.Don't know

145) Has your child had an illness with a cough at any time in the last 2 weeks?

cough_quest_12m

This question means whether the infant had an illness with a cough at any time in the last 2 weeks or not.

1. Yes
0. No

98.Don't know
99.Missing

If No, then go to question number 33.

146) When your child had an illness with a cough in the last 2 weeks, did he/she breathe faster than usual with short, rapid breaths or have difficulty breathing?

apnea_quest_12m

The important thing is when the infant had an illness with a cough in the last 2 weeks, whether she breathe faster than usual with short, rapid breaths or have difficulty breathing or not. The purpose of this question is to determine the possibility of pneumonia cases.

1. Yes
0. No

97.NA
98.Don't know

99.Misssing

147) Has your infant had dengue fever in the last 3 months?

dengue_quest_12m

*This question means whether **infant has** had dengue fever **or not or don't know in the last 3 months.***

1. Yes
0. No
98.Don't know
99.Missing

148) Has your child been feeding poorly or refusing food in the last 2 weeks?

refuse_eat_quest_12m

*This question means whether **infant has** been feeding poorly or refusing food in the last 2 weeks **or not or don't know.***

1. Yes
0. No
98.Don't know
99.Missing

149) Has your infant been given worm medicine in the last 3 months?

wormmed_quest_12m

The important thing is whether the infant has been given worm medicine in the last 3 months or not. The consumption of worm medicine can eradicate the worms in infant gut.

1. Yes
0. No
98.Don't know
99.Missing

150) Has your infant been sick since born?

ever_sick_quest_12m

*This question means whether **infant has** been sick since born **or not or don't know.***

1. Yes
0. No
99.Missing

If No, then go to question number 39.

151) Did you seek advice for any of the illnesses from health workers when (NAME) got sick in the last 3 months?

seek_health_quest_12m

This question means whether mother seek advice for any of the illnesses from health workers or not.

1. Yes
0. No
97.NA
98.Don't know
99.Missing

If No, then go to question number 39.

152) If 1, which health workers you visited (most often)in the last 3 months?

health_quest_12m

The purpose of this question is to know which health workers that mother visited (most often) in the last 3 months. Please choose one. (NAME refers to child's name)

1. Doctor
2. Nurse
3. Midwife
4. Cadre

97.NA

99.Missing

66.Other

153) Currently, has (NAME) received any medications?

medicine_quest_12m

The important thing is whether (NAME) currently received any medications or not.

1. Yes

0. No

99.Missing

If No, then go to question number 41.

154) Could you please show the medication (if you still keeping the blister/package/bottle)?

medicine_show_quest_12m

Whether it is yes/No, please specify (name, dose, days of administration) of all medications received:

Ask the mother to specify the name, dose, days of administration of all medications received. Ask mother to show the medication if she still keeping the blister/package/bottle. The purpose of showing the blister/package/bottle is to make sure the drug consumed by the infant. We can also know the dosage given to infant precisely.

155) Has (NAME) received any drug in the last 1 month?

drug_onemonth_quest_12m

This question means whether (NAME) has received any drug in the last 1 month ***or not or don't know.***

1. Yes

0. No

97.NA

99.Missing

If No, then go to question number 44

156) Has (NAME) received any antibiotics in the last 1 month?

ab_1m_quest_12m

Ask the mother whether (NAME) received or not any antibiotics in the last 1 month. Antibiotic consumption can influence gut micro bacteria examined in infant. reference

1. Yes
0. No
97.NA
98.Don't Know
99.Missing

157) Has (NAME) received any antifungal in the last 1 month?

af_quest_12m

Ask the mother whether (NAME) received or not any antifungal in the last 1 month. Antifungal consumption can influence gut parasite examined in infant. reference

1. Yes
0. No
97.NA
98.Don't Know
99.Missing

158) When is the last time (NAME) received antibiotics?

ab_quest_12m

Ask the mother when the the last time (NAME) received antibiotics. You can fill in this question in date format or using adverb of time such as 2 weeks ago.

159) What is the name of the last antibiotics?

ab_ name_quest _12m

Fill in this question with the name of the last antibiotics that (NAME) consumed since born.

160) How many days was the antibiotics given?

ab_ day_quest _12m

Fill in this question with the duration of the last antibiotics that (NAME) consumed since born.

161) When is the last time (NAME) received antifungal?

af_ quest _12m

Ask the mother when the the last time (NAME) received antifungal. You can fill in this question in date format or using adverb of time such as 3 weeks ago.

162) What is the name of the antifungal?

af_name_quest_12m

Fill in this question with the name of the last antibiotics that (NAME) consumed since born.

163) How many days was the antifungal given?

af_ day_quest _12m

Fill in this question with the duration of the last antifungal that (NAME) consumed since born.

164) How did you give breastfeeds and complementary food during the time your baby was sick in the last 3 months (in general)?

cf_sick_quest_12m

The important thing is mother give the same number of breastfeeds and the same amount of complementary food during the time her baby was sick or not.

1. More than usual
2. Less than usual
3. Same as usual

97.NA
99.Missing

165) If 1, please specify (type and number):

cf_type_ quest _12m

166) Do you withhold certain food from your infant when (NAME) is ill in the last 3 months (in general)?

cf_limit_quest_12m

Fill in this question whether mother withhold certain food from her infant when (NAME) is ill (in general) or not.

1. Yes
0. No

97.NA
98.Don't Know
99.Missing

167) If yes, please specify:

cf_limit_name_quest_12m

Ask the mother to specify what the certain food is. Try to get the detail information.

168) Do you give any special foods to your infant after illness in the last 3 months (in general)?

cf_after_sick_quest_12m

Fill in this question whether mother give special foods to her after illness (in general) or not.

1. Yes
0. No

97.NA
98.Don't Know
99.Missing

169) If 1, please specify:

cf_as_name_quest_12m

Ask the mother to specify what the special food is. Try to get the detail information.

170) Has your infant been hospitalized in the last 3 months?

hospital_ever_quest_12m

The important thing is whether infant has been hospitalised or not or don't know.

1. Yes

0. No
98.Don’t Know
99.Missing

If No, then go to question number 59

171) If 1, how many times has your infant been hospitalised?
hospital_number_quest_12m
Ask the mother to specifyhow many times her infant has been hospitalised or don’t know.
1. 1
2. 2
3. 3+
98.Don’t know
99.Missing

172) Please specify the reason(s) for hospitalisation:
hospital_reason_quest_12m
Ask the mother to specifythe reason(s) for hospitalisation.

173) Note infant birthweight (gram) as written in the KMS :
birthweight_KMS_quest_12m

____

174) Has your infant been immunised?
immunisation_quest_12m
The important thing is whether the infant has been immunized or not. It is very important that you ask each question exactly as it is written on the questionnaire. Even though the mother said only once, choose ‘1’ choice button.
1. Yes
0. No
99.Missing

175) Can mother show the infant’s immunisation card?
immunisation_card_quest_12m
Ask the mother whether mother can show the infant’s immunisation card or not. Be sure that she understands that the “card” refers to vaccination documentation. In some cases the mother may not be willing to take time to look for the vaccination card, thinking that you are in a hurry. Encourage her to look for (NAME’s) card. It is highly desirable to obtain written documentation of the child’s immunization history; therefore, be patient if the respondent needs to search for the card.
1. Yes
0. No
99.Missing

If 1, please TAKE PICTURE of the IMMUNISATION CARD, and fill these questions based on the information in IMMUNISATION CARD! If NOT, Ask the mother when the

interviewer can go back and see thatIMMUNISATION CARD. If there is not card, fill DONT KNOW for questions number 55-59.

176) Tuberculosis vaccination (BCG)?

bcg_quest_12m

See in the card whether the infant has received tuberculosis vaccination (BCG) or not.

1. Yes
0. No
97.NA
98.Don't know
99.Missing

177) Polio vaccination?

polio_quest_12m

See in the card whether the infant has received Polio vaccination or not.

1. Yes
0. No
97.NA
98.Don't know
99.Missing

178) Diptheria, Pertussis and Tetanus (DPT) vaccination?

dpt_quest_12m

See in the card whether the infant has received Diptheria, Pertussis and Tetanus (DPT)or not.

1.Yes
0.No
97.NA
98.Don't know
99.Missing

179) Measles vaccination?

measles_quest_12m

See in the card whether the infant has received Measles vaccination or not.

1. Yes
0. No
97. NA
98. Don't know
99. Missing

180) Hepatitis B vaccinations?

hep_b_quest_12m

See in the card whether the infant has received Hepatitis B vaccinations or not.

1. Yes
0. No
97. NA
98. Don't know
99. Missing

181) At any time in the past 3 months, has anyone come into your house to spray the inside walls against mosquitoes?

fogging_quest_12m

Fill in the question whether the house has been fogged inside walls against mosquitoes or not. If mother doesn't know, choose 'don't know'.

1. Yes
0. No
98.Don't know
99.Missing

182) Does your household have any insecticide treated mosquito nets that can be used while sleeping?

bednet_ quest _12m

Fill in the question whether household have any insecticide treated mosquito nets that can be used while sleeping or not.

1. Yes
0. No
98. Don't know
99. Missing

If No, then go to question number 70

183) If 1, did your infant sleep under an insecticide treated mosquito net last night?

bednet_child_quest_12m

The important of this question is whether the infant sleep under an insecticide treated mosquito net last night or not.

1. Yes
0. No
97. NA
98. Don't know
99. Missing

184) Who usually cooks your infant's food in the last 3 months?

cook_ quest_12m

Ask the mother who usually cooks the infant's food to fill in this question. Remember to choose only one.

1. Myself (mother)
2. Mother in law
3. Mother
4. Caregiver
5. Sibling
66. Other

185) Who usually feeds your infant in the last 3 months?:

feed_quest_12m

Ask the mother who usually feeds her infant. Please choose one answer.

1. Myself (mother)
2. Mother in law
3. Mother

4. Caregiver
5. Sibling
66. Other

186) Do you always cover food until it is eaten?

food_cover_ quest _12m

Fill in the question whether mother always cover food until it is eaten or not.

1. Yes
0. No
99. Missing

187) There are key moments when you need to wash your hands to prevent germs from reaching food. What are these key moments?

In this question you can choose more than one answer. Fill in the key moments when mother need to wash her hands to prevent germs from reaching food.

1. After going to the toilet/latrine wash_toilet_ quest _12m
2. After cleaning the baby's bottom/changing a baby's nappy wash_diaper_ quest _12m
3. Before preparing/handling food wash_cook_ quest _12m
4. Before feeding a child wash_feed_ quest _12m
5. Before eating wash_eat_ quest _12m
6. After handling raw food wash_raw_ quest _12m
7. After handling garbage wash_trash_ quest _12m
98. Don't know wash_dk_ quest _12m
66. Other wash_othr_ quest _12m

188) Did (NAME) drink anything from a bottle with a nipple yesterday or last night?

bottle_quest_12m

Ask mother whether the infant or child drank anything from a bottle with a nipple yesterday. Baby bottles are not recommended, because they are very difficult to clean. Spoons, cups, and gourds may also be dirty, but bottles are even more likely to be contaminated and to make the baby sick. Fill in this question whether (NAME) drank anything from a bottle with a nipple yesterday or last night or not.

1. Yes
0. No
98. Don't know
99. Missing

189) Was (NAME) breastfed of did he/she consume breastmilk during the day or at night?

yest_breast_12m

Fill in this question whether the infant was breastfed during all day or not.

1. Yes
0. No
98. Don't know
99. Missing

If 1, continue to question 78

190) If No, for how long did you breastfeed (NAME)? (in months)

long_quest_12m

Ask the mother how long she breastfeed (NAME) in month.

191) Why did you stop breastfeeding?

stop_quest_12m

The important thing is the reason why the mother stopped breastfeeding. Then fill in this question.

1. Mother pregnant
2. Mother unwell
3. Mother tired of breastfeeding
4. Introduced solids
5. Breast milk making child sick
6. Not enough milk
7. Infant refused breast milk
8. Formula milk is better

66. Other
97. NA
98. Don't know
99. Missing

Now I would like to ask you about other foods your child ate over the last 24 hours (question 78-87). I am interested in whether your child had the item even if it was combined with other foods. Yesterday, did your child eat:

192) Grains, roots and tubers?

Some foods in the list are listed as a single item – for example, white potatoes – but may usually be eaten in a soup or stew. If the infant or child has eaten a mixed food like a soup,or stew, record all the food groups in the mixed food. For example, if the child ate a stew ofpotatoes, maize, there should be a check mark for each of the two food groups that contain these foods. Do not check off foods that have been added in very small amounts, or for seasoning.

	Code	1 Yes	0 No	99 Missing
Porridge, bread, rice, maize, noodles or other foods made from grains	rice24_quest _12m			
White potatoes, white yams, cassava or any other foods made from roots	root24_quest _12m			

193) Any foods made from beans (kidney beans, mung beans, soy beans, tofu, tempeh), peas, lentils, nuts (peanuts) or seeds?

nuts24_quest_12m

The important thing is whether infant eat any foods made from beans (kidney beans, mung beans, soy beans, tofu, tempeh), peas, lentils, nuts (peanuts) or

seeds or not. Do not check off foods that have been added in very small amounts, or for seasoning.

1. Yes
0. No
99. Missing

194) Any dairy products?

Some foods in the list are listed as a single item. If the infant or child has eaten a mixed food record all the food groups in the mixed food. Please mark '1' or 'no' button

		1 Yes	0 No	99 Missing
Milk, such as tinned, powdered or fresh animal milk	milk24_quest_12m			
Formula milk	formula24_quest_12m			
Yogurt or drinking yogurt	yoghurt24_quest_12m			
Cheese or other dairy products	cheese24_quest_12m			

195) Any flesh foods?

Some foods in the list are listed as a single item. If the infant or child has eaten a mixed food record all the food groups in the mixed food. Please mark '1' or 'no' button. Do not check off foods that have been added in very small amounts, or for seasoning. For example, if a spoon of fish powder is added to a pot of stew, do not record that the infant or child has eaten fish

	Code	1 Yes	0 No	99 Missing
Liver, kidney, heart or other organ meats	organ24_ quest _12m			
Any meat, such as beef, pork, lamb, goat, chicken or duck	flesh24_ quest _12m			
Fresh or dried fish, shellfish or seafood	seafood24_ quest _12m			

196) Chicken eggs, duck eggs or qual eggs?

egg24_ quest _12m

The important thing is whether the infant eat chicken eggs, duck eggs or qual eggs or not.

1. Yes

0. No
99. Missing

197) Yellow/red/orange fruits and/or any dark green leafy vegetables?

Some foods in the list are listed as a single item – for example, pumpkin – but may usually be eaten in a soup or stew. If the infant or child has eaten a mixed food like a soup, or stew, record all the food groups in the mixed food. For example, if the child ate a stew of pumpkin, papaya, and green leaves, there should be a check mark for each of the three food groups that contain these foods. Do not check off foods that have been added in very small amounts, or for seasoning. For example, If one fifth of tomato is included in the family pot, do not record that as an "other fruit or vegetable."

		1 Yes	0 No	99 Missing
Pumpkin, carrots, tomatoes, squash or sweet potatoes that are yellow or orange inside	yellow24_quest _12m			
Any dark green leafy vegetables, e.g. spinach, cassava leaves, water spinach, broccoli, caweightage, lettuce	green24_ quest _12m			
Ripe mangoes (fresh or dried), ripe papayas	orange24_quest _12m			

198) Any other foods mentioned below?

Some foods in the list are listed as a single item – for example, biscuit – but may usually be eaten together such as buttered biscuit. If the infant or child has eaten a mixed food like buttered biscuit, record all the food groups in the mixed food. For example, if the child ate a buttered biscuit, there should be a check mark for each of the two food groups that contain these foods. Do not check off foods that have been added in very small amounts, or for seasoning. For example, If one chili pepper is included in the family pot, do not record that as an "other fruit or vegetable."

	Code	1 Yes	0 No	99 Missing
Any oils, fats, or butter or foods made with any of these	oil24_ quest _12m			
Any surgary foods, such as chocolates, sweets, candies, pastries, cake or biscuits	sugar24_ quest _12m			
Any sweet beverage, such as soda, fruit flavoured drink	beverage24_quest_12m			

Any tea and/or coffee	coffee24_ quest _12m			
Condiments for flavour, such as chilies, spices, herbs, soy sauce, ketchup, or fish powder	spice24_ quest _12m			

199) How many times did (NAME) eat foods, that is meals and snacks other than liquids yesterday during the day or at night?

eat_yest_ quest _12m

This question asks about how many times the infant or child ate solid/semi-solid foods as meals and snacks 1terday. Ask the mother/caregiver the question just as it is written. Solid/semi-solid foods include family foods, and also many special dishes prepared for infants. Thick soups and stews should be included. Thick porridges are also included. Very thin, watery soups and gruels should not be included because infants and young children do not get enough energy (calories) from very thin soups and gruels. Liquids do not count for this question. Also, very small snacks, such as a bite or two of someone else's food, should not be counted.

1. 1
2. 2
3. 3
4. 4
5. 5
6. 6+

97. NA

200) How many meals (excluding snacks) did you feed your infant yesterday?

meal_ yest_quest _12m

This question asks about how many times the infant or child ate solid/semi-solid foods as meals 1terday. Ask the mother/caregiver the question just as it is written. Solid/semi-solid foods include family foods, and also many special dishes prepared for infants.

1. 1 meals/day
2. 2 meals/day
3. 3 meals/day
4. 4 meals/day
5. 5 meals/day
6. 6+ meals/day

97. NA
98. Don't know
99. Missing

201) Did your child eat any food prepared with flour yesterday during the day or night?

flour_yest_ quest _12m

This question asks whether infant ate or not any food prepared with flour that was fortified with micronutrients yesterday.

1. Yes, please write the brand:

0. No

97. NA
98. Don't know
99. Missing

202) *Has your infant ever received salt fortified by iodine?*

salt_quest_12m

This question asks whether infant ever received salt fortified by iodine or not. Iodine is important for children growth and intelligent. Iodine deficiency also can cause endemic goiter.

1. Yes
0. No
98. Don't know
99. Missing

203) *Please show the salt used in your household. Write down the brand:*

salt_name_ quest _12m

This question asks the mother to show the salt used in her household. Write down the brand.

204) *Has your infant ever received Vitamin A supplements in the last 3 months? (Please show vitamin A capsules)*

vit_a_ quest _12m

The purpose of question 90 is to find out whether or not the infant/child received a doseof vitamin A in the last 6 months. Lack of vitamin A causes children to get sick more easily andin extreme cases lack of vitamin A can cause eye damage and blindness. Vitamin A supplements are given because many children do not get enough vitamin A from the foods theyeat.

You will need examples of the vitamin A capsules or ampules that have been used in the project area. Show the mother/caregiver the capsule or ampule to help her remember if her infant or child ever received one.

1. Yes
0. No
98. Don't know
99. Missing

205) *Has your infant received multivitamin and minerals pills or syrup in the last month?*

multivitamin_ quest _12m

The purpose of question 28 is to find out whether or not the infant/child received a dose of multivitamin and minerals pills or syrup in the last month.

1. Yes
0. No
98. Don't know
99. Missing

206) *Has your infant received sprinkles (TABURIA) in the last 3 months? (Please show TABURIA)*

taburia_ quest _12m

This question asks whether infant has received sprinkles (TABURIA) in the last 6 months or not. (Please show TABURIA)

1. Yes

0. No
98. Don't know
99. Missing

If No or Don't Know , then go to question number 100

207) From whom have you received the sprinkles (TABURIA)?

taburia_whom_ quest _12m

The purpose of question 93 is to find out from whothe mother has received the sprinkles (TABURIA).

1. Puskesmas staff
2. Village Midwife
3. Cadres
66. Other
97. NA
99. Missing

208) When did you receive it?

taburia_when_ quest_12m

The purpose of question 94 is to find out the time that mother has received the sprinkles (TABURIA). TABURIA better been given at 6 month until 24 month old infant.

1. This month
2. Last month
3. Two months ago
4. Three months ago
5. Four months ago (or more)
97. NA
99. Missing

209) How many sachets of sprinkles have you received in the last 3 months?

taburia_number_ quest_12m

The purpose of question 95 is to find out how many sachets of sprinkles mother has received in the last 3 months

1. <15 sachets
2. 15 – 30 sachets
3. 30 – 60 sachets
4. >60 sachets
97. NA
98. Don't know
99. Missing

210) How many sachets of sprinkles have you given to (NAME) in the last 3 months?

taburia_eat_ quest _12m

The purpose of question 96 is to find out how many sachets of sprinkles mother give to (NAME) in the last 3 months.

1. <5 sachets
2. 5 – 10 sachets
3. 10 – 15 sachets
4. >15 sachets

97. NA
99. Missing

211) How did you give the sprinkles to (NAME)?

This question asks how mother give the sprinkles to (NAME). It should be given once every two days.

1. Twice a day taburia_twice_quest_12m
2. Every day taburia_once_quest_12m
3. Every other day taburia_days_quest_12m
4. Sprink it into infant porridge (cool) taburia_cold_quest_12m
5. Sprink it into infant porridge (hot) taburia_hot_quest_12m
6. Mix it with porridge/soup while cooking taburia_cook_quest_12m
7. Never taburia_never_quest_12m

97. NA taburia_na_quest_12m
66. Other othr6_quest_12m

212) How did (NAME) react to the addition of sprinkles (Taburia) to his/her food?

taburia_reaction_quest_12m

This question asks how (NAME) did react to the addition of sprinkles (Taburia) to his/her food.

1. Dislike very much
2. Dislike a little
3. Not sure
4. Like a little
5. Like very much

97. NA

213) Do you know what is the function/benefit of giving sprinkles to your infant? Please mention:

taburia_benefit_quest_12m

The purpose of question 99 is to find out the knowledge of mother about the function/benefit of giving sprinkles to infant.

---

214) Have you regularly brought your infant to Posyandu?

posyandu_quest_12m

The purpose of question 100 is to find out whether mother regularly brings her infant to Posyandu or not.

1. Yes, once per month
2. Yes, once every two month
3. Sometimes (1-2 times)
4. Never

98. Don’t know

215) How many snacks did you feed your infant yesterday?

snack_yest_quest_12m

This question asks about how many times the infant or child ate solid/semi-solid foods as snacks yesterday. Ask the mother/caregiver the question just as it is written. Solid/semi-solid foods include family foods, and also many special dishes prepared for infants.

1. 1 snacks/day

2. 2 snacks/day
3. 3 snacks/day
4. 4 snacks/day
5. 5 +snacks/day
98. Don't know
97. NA

216) Do you give your children a separate plate?
plate_quest_12m
The important thing is whether mother gives her children a separate plate or not.
1. Yes
0. No
97. NA
98. Don't know
99. Missing

217) Did you add ingredients to your infant's porridge such as mashed vegetables, beans, fruits, eggs, and small fish?
porridge_add_quest_12m
The important thing is whether mother add ingredients to the infant's porridge such as mashed vegetables, beans, fruits, eggs, and small fish or not.
1. Yes
0. No
97. NA
98. Don't know
99. Missing

218) During the last two weeks did your infant eat any of the following foods? *(ALLOW MULTIPLE ANSWERS, READ THE LIST)*
The important thing is whether during the last two weeks infant eat any foods listed in the question. Encourage mother to multiple answers, but remember not to mention the foods listed.
The important thing is whether during the last two weeks infant eat any foods listed in the question. Encourage mother to multiple answers, but remember not to mention the foods listed.
1. Cassava leaves cassava_leaf_quest_12m
2. Pumpkin leaves pumpkin_leaf_quest_12m
3. Carrots carrot_quest_12m
4. Pumpkin pumpkin_quest_12m
5. Groundnuts nuts2_quest_12m
6. Potato leaves potato_leaf_quest_12m
7. Mangoes mango_quest_12m
8. Papaya papaya_quest_12m
9. Eggs egg3_quest_12m
10. Meat flesh2_quest_12m
11. Fish fish_quest_12m
12. Banana banana_quest_12m
13. Avocado avocado_quest_12m
14. Sweet potato sweet_potato_quest_12m

15. Animal milk milk3_quest_12m
98. Don't know dk_quest_12m
97. NA na_quest_12m

219) Are you the person who fed [NAME] yesterday?

mom_feed_quest_12m

The purpose of question 105 is to confirm whether mother was someone that fed the infant 1terday or not.

1. Yes
0. No
97. NA
99. Missing

220) Yesterday, at the main meal, did [NAME] eat all the food you thought he/she should?

eat_all_quest_12m

The purpose of question 106 is whether [NAME] eat all the food mother's thought he/she should or not.

1. Yes
0. No
97. NA
98. Don't know
99. Missing

221) During this meal, were you or another caregiver sitting with your infant?

sit_together_quest_12m

This question asks whether mother or another caregiver were sitting with infant during this meal or not.

1. Yes
0. No
97. NA
99. Missing

222) If sitting with the infant, how were you two positioned?

position_quest_12m

The question asks how about the position of mother/or caregiver to infant. Whether can see the infant's face or not.

1. Mother/caregiver positioned so she could NOT see infant's face
2. Mother/caregiver positioned so she could see infant's face frontways or sideways
97. NA
99. Missing

223) How was the infant positioned?

infant_position_quest_12m

The question asks how about the position of infant.

1. Infant held in mother or caregiver's arms
2. Infant lying in mother or caregiver's lap
3. Infant sitting in mother or caregiver's lap
4. Infant standing up or running around

5. Infant sitting on a chair, stool, mat or bed
97. NA
66. Other position_othr_quest_12m

224) *Did your infant refuse to eat an offered mouthful of food?*
refuse_quest_12m
This question asks whether infant refused to eat an offered mouthful of food or not.
1. Yes
0. No
97. NA
99. Missing

225) *Did your infant cry during the meal yesterday?*
cry__quest__12m
This question asks whether infant cried during the meal yesterday or not
1. Yes
0. No
97. NA
99. Missing

226) *Did your infant try to leave the meal early yesterday?*
leave_early_quest_12m
This question asks whether infant try to leave the meal early 1terday or not
1. Yes
0. No
97. NA
99. Missing

227) *Did you have to control your infant while eating, such as by holding his/her head or body?*
control_quest_12m
The purpose of question 113 is whether mother has to control your infant while eating, such as by holding his/her head or body.
1. Yes
0. No
97. NA
99. Missing

228) *Yesterday at the evening meal, how was your infant behaviour?*
behaviour_quest_12m
The purpose of question 114 is to find out infant behaviour on yesterday at the evening meal.
1. More fussy about eating than usual
2. Eating as she or he usually eats
3. Less fussy about eating than usual
98. Don't know
97. NA

229) *Was your infant sick yesterday?*
sick_yest_quest_12m
This question asks whether infant sick yesterday or not.
1. Yes
0. No
99. Missing

230) *Is your child able to feed themselves some of the meal?*
feed_themselves_quest_12m
This question asks whether infant able to feed themselves some of the meal or not
1. Yes
0. No
99. Missing

231) *If 1, at what age in months are children able to START feeding themselves some of the meal?*
feed_themself_age_quest_12m
The purpose of question 117 is to find out the specific age in months the children able to START feeding themselves some of the meal.

232) *Do you still often give porridge to [Name] ?*
porridges_still_quest_12m
1. Yes
0. No
99. Missing

233) *If 1, which type of porridge do you usually make for [Name]? Thick porridge or watery porridge?*
type_porridges_quest_12m
1. Thick porridge
2. Watery porridge
97. NA
98. Don't know
99. Missing

234) *Why do you choose that type of porridges?*
reason_quest_12m
The purpose of question 120 is to find out the reason why mother has chosen the type of porridge she made. .
1. Because the watery porridge is easier to be eaten.
2. Because the thick porridge is more nutritious or because it is prepared with different types of foods or ingridients (food diversity)
97. NA
98. Don't know
66. Other reason_othr_quest_12m

235) *To feed their children, many mothers give them rice porridge. Please tell me some ways to make rice porridge more nutritious or better for your baby's health.*

(Probe if necessary: Which foods or types of food can be added to rice porridge make it more nutritious?)
porridge_better_quest_12m
For this question, you have to ask the mother to explain some ways to make rice porridge more nutritious or better for her baby's health.

1. Animal-source foods (meat, poultry, fish, liver/organ meat, eggs, etc.)
2. Pulses and nuts: flours of groundnut and other legumes (peas, beans, lentils, etc.), sunflower seed, peanuts, soybeans
3. Vitamin-A-rich fruits and vegetables (carrot, orange-fleshed sweet potato, yelow pumpkin, mango, papaya, etc.)
4. Green leafy vegetables (e.g. spinach)
5. Energy-rich foods (e.g. oil, butter)

66. Other, please specify: ______________________________

98. Don't know

99. Missing

236) *Children can start to be fed these foods in :*

	Code	*1. 0-3 months*	*2. 4-5 months*	*3. 6 months*	*4. 7-8 months*	*5. 9-11 months*	*6. 12-24 months*	*7. >24 months*	*97. NA*
Mashed egg	start_egg_12m								
Mashed chicken	start_chicken_12m								
Mashed fish	start_fish_12m								
Mashed vegetables	start_veg_12m								
Mashed fruits	start_fruit_12m								
Mashed tofu/tempe	start_tofu_12m								
Mashed family meals	start_fam_12m								

237) *What is your opinion if the 6-9 months baby is fed instant porridge every day?*
instant_porridge_12m

1. Strongly agree
2. Agree
3. Disagree
4. Strongly disagree

98. Don't know
99. Missing

238) What is your reason related to your answer in question 123?

reason_instant_12m

1. Healthy
2. Expensive
3. Nausea/ vomiting
4. Dislike
5. Easy
6. Cheap
7. Not healthy/ thin child
8. Told not to do (by midwife, parents / others)
9. Told to do (by midwife, parents/ others)

98. Don't know
99. Missing

239) What is your opinion if 6 months baby is fed fish?

fed_fish_12m

1. Strongly agree
2. Agree
3. Disagree
4. Strongly disagree

98. Dont't know
99. Missing

240) What is your reason related to your answer in question124?

reason_fish_12m

1. Worm infection
2. Expensive
3. Nausea/ vomiting
4. Dislike
5. Unready digestion tract
6. Fishbone choking
7. Not healthy/ thin child
8. Told not to do (by midwife, parents / others)
9. Told to do (by midwife, parents/ others)
10. Nutritious, rich of calcium
11. Healthy

98. Don't know
99. Missing

AGES and STAGES 12 months

141) Interviewer :
interviewer_stages_12m

Use your initial every time you have to fill in the interviewer question.
1. AD : Aly Diana
2. DEL : Dimas Erlangga Luftimas
3. M : Monik
4. R : Rima
5. HF : Hana Fauzia
6. NN : Ninda
7. TF : Tri Mulya Fitriasari
8. S : Sofa Rahmannia
9. HK : Hendro Kasmanto
66. Other

142) Date of interview (dd/mm/yyyy):
date_stages_12m
Use format day/month/year to fill in this interviewer question.

143) Mother's name:*
name_mom_stages_12m

It is better to fill in the full name of mother. If it is not possible, you can fill in the forename, middle name or nick name. Please match the name that was told by mother with the name written in the paper.*

144) Mother's ID number:*
id_stages_12m

It is no need of asking this question. It is written in the paper given to you. Please re-check once again whether you have typed the ID number correctly.*

COMMUNICATION

145) Has your baby been able to make two similar sounds such as"da-da" "ba-ba" "ga-ga" (the sound does not have to have a certain meaning)?
q1_similar_sound_12m
1 yes
2 sometimes
0 no

146) Observed/ reported
1 observed
q1_observed_12m
2 reported
q1_reported_12m
3 discrepancy between observation and report
q1_discrepancy_12m

147) If you ask he/she to do at least one motion of children's games, could she/he do that even if you do not provide examples (eg, 'bye', 'peekaboo', 'applause')?

q2_do_one_motion_12m

1 yes

2 sometimes

0 no

148) Observed/ reported

1 observed

q2_observed_12m

2 reported

q2_reported_12m

3 discrepancy between observation and report

q2_discrepancy_12m

149) Has your baby been able to follow simple commands, such as "come here", "Give it to me", "put it back" without you using gestures?

q3_simple_command_12m

1 yes

2 sometimes

0 no

150) Observed/ reported

1 observed

q3_observed_12m

2 reported

q3_reported_12m

3 discrepancy between observation and report

q3_discrepancy_12m

151) Has your baby been able to mention three words like "mama", "baba", "dada" (the word is the sound that your baby consistently said to shows someone or something)?

q4_three_words_12m

1 yes

2 sometimes

0 no

152) Observed/ reported

1 observed

q4_observed_12m

2 reported

q4_reported_12m

3 discrepancy between observation and report

q4_discrepancy_12m

153) When you asked your baby "Where is ball (hat, shoes,etc)", has your baby been able to look to that object? (please make sure that the object is exist, and answer "yes" if she/he knows the object)

q5_look_object_12m
1 yes
2 sometimes
0 no

154) Observed/ reported

1 observed
q5_observed_12m
2 reported
q5_reported_12m
3 discrepancy between observation and report
q5_discrepancy_12m

155) When your baby wants something, does he /she tell you by pointing the object which he/she wants?

q6_point_object_12m
1 yes
2 sometimes
0 no

156) Observed/ reported

1 observed
q6_observed_12m
2 reported
q6_reported_12m
3 discrepancy between observation and report
q6_discrepancy_12m

GROSS MOTOR

157) When clinging to the furniture, does your baby bent his/her legs to take the toys off the floor and then stand back up?

q7_bent_legs_12m
1 yes
2 sometimes
0 no

158) Observed/ reported

1 observed
q7_observed_12m
2 reported
q7_reported_12m
3 discrepancy between observation and report
q7_discrepancy_12m

159) When clinging to the furniture, does your baby lower his/her own body in a controlled manner (without falling)?

q8_lower_body_12m

1 yes
2 sometimes
0 no

160) Observed/ reported

1 observed
q8_observed_12m
2 reported
q8_reported_12m
3 discrepancy between observation and report
q8_discrepancy_12m

161) Is your baby able to walk beside furniture with holding using just one hand?

q9_walk_holding_one_hand_12m

1 yes
2 sometimes
0 no

162) Observed/ reported

1 observed
q9_observed_12m
2 reported
q9_reported_12m
3 discrepancy between observation and report
q9_discrepancy_12m

163) When you hold both of your baby's hands, just to balance his/her body, does she/he walk a few steps without tripping or falling? (If your baby was able to walk alone, answer "yes" for this question)

q10_hold_both_steps_12m

1 yes
2 sometimes
0 no

164) Observed/ reported

1 observed
q10_observed_12m
2 reported
q10_reported_12m
3 discrepancy between observation and report
q10_discrepancy_12m

165) When you hold one of your baby's, just to balance his/her body, does she/he walk few steps ahead? (If your baby was able to walk alone, answer "yes" for this question)

q11_hold_one_steps_12m

1 yes

2 sometimes
0 no

166) Observed/ reported

1 observed
q11_observed_12m
2 reported
q11_reported_12m
3 discrepancy between observation and report
q11_discrepancy_12m

167) Does your baby stand up by him/herself and walk a few steps ahead?

q12_stand_themselves_12m
1 yes
2 sometimes
0 no

168) Observed/ reported

1 observed
q12_observed_12m
2 reported
q12_reported_12m
3 discrepancy between observation and report
q12_discrepancy_12m

FINE MOTOR

169) After trying one to two times, can your baby take a string with his/her thumb and forefinger (the rope may be attached to the toy)?

q13_thumb_forefinger_12m
1 yes
2 sometimes
0 no

170) Observed/ reported

1 observed
q13_observed_12m
2 reported
q13_reported_12m
3 discrepancy between observation and report
q13_discrepancy_12m

171) Has your baby been able to take the crumbs with the tip of the thumb and fingers? He/she might put his hands or arms on the table when doing it.

q14_tip_fingers_12m
1 yes
2 sometimes
0 no

172) Observed/ reported

1 observed
q14_observed_12m
2 reported
q14_reported_12m
3 discrepancy between observation and report
q14_discrepancy_12m

173) Has your baby been able to put his/her little toy down without dropping it and release the toy out of his hands?
q15_put_toy_down_12m
1 yes
2 sometimes
0 no

174) Observed/ reported
1 observed
q15_observed_12m
2 reported
q15_reported_12m
3 discrepancy between observation and report
q15_discrepancy_12m

175) Without put your baby hands on the table, does she/he take the crumbs using the tip of thumb and one finger?
q16_crumbs_thumb_finger_12m
1 yes
2 sometimes
0 no

176) Observed/ reported
1 observed
q16_observed_12m
2 reported
q16_reported_12m
3 discrepancy between observation and report
q16_discrepancy_12m

177) Does your baby throw a small ball by moving forward his/her arms? (If he/she only drops the ball, answer "no" for this question)
q17_throw_moving_arm_12m
1 yes
2 sometimes
0 no

178) Observed/ reported
1 observed
q17_observed_12m
2 reported
q17_reported_12m
3 discrepancy between observation and report

q17_discrepancy_12m

179) Has your baby been able to open sheets of book?

q18_open_books_12m

1 yes
2 sometimes
0 no

180) Observed/ reported

1 observed
q18_observed_12m
2 reported
q18_reported_12m
3 discrepancy between observation and report
q18_discrepancy_12m

PROBLEM SOLVING

181) If your baby holding a small toy in each hand, does your baby clap the toys together? (Like clapping cakes)

q19_clap_toys_12m

1 yes
2 sometimes
0 no

182) Observed/ reported

1 observed
q19_observed_12m
2 reported
q19_reported_12m
3 discrepancy between observation and report
q19_discrepancy_12m

183) Can your baby stirring or trying to get crumbs inside a transparent bottle? (Like a plastic soda bottle or baby bottle)

q20_stirring_transparent_bottle_12m

1 yes
2 sometimes
0 no

184) Observed/ reported

1 observed
q20_observed_12m
2 reported
q20_reported_12m
3 discrepancy between observation and report
q20_discrepancy_12m

185) After seeing you hide a small toy under the paper or clothes, is your baby able to find it? (Make sure the toy is completely hidden)

q21_find_hid_toys_12m

1 yes
2 sometimes
0 no

186) Observed/ reported

1 observed
q21_observed_12m
2 reported
q21_reported_12m
3 discrepancy between observation and report
q21_discrepancy_12m

187) When you put a small toy into the bowl or the box, does your baby follow you to put his/her toy, even maybe he/she doesn't let it go? (If he/she has let her/his toy inside the bowl or the box, answer "yes" for this question)

q22_toys_into_bowl_12m

1 yes
2 sometimes
0 no

188) Observed/ reported

1 observed
q22_observed_12m
2 reported
q22_reported_12m
3 discrepancy between observation and report
q22_discrepancy_12m

189) Does your baby put two small toys, one by one, into the bowl or the box? (You might show him/her how to do it)

q23_two_toys_12m

1 yes
2 sometimes
0 no

190) Observed/ reported

1 observed
q23_observed_12m
2 reported
q23_reported_12m
3 discrepancy between observation and report
q23_discrepancy_12m

191) When you make a doodle on the paper using crayon, does your baby make a doodle too? (If he/she has made a doodle by him/herself, answer "yes" for this question)

q24_make_doodle_12m

1 yes

2 sometimes
0 no

192) Observed/ reported
1 observed
q24_observed_12m
2 reported
q24_reported_12m
3 discrepancy between observation and report
q24_discrepancy_12m

PERSONAL-SOCIAL

193) When you stretch out your hand and ask forhis/her toy, does your baby to give it to your hand although he/she doesn't want to let go? (If he/she is willing to give the toy to your hand give a"yes" to this question)
q25_willing_to_give_12m
1 yes
2 sometimes
0 no

194) Observed/ reported
1 observed
q25_observed_12m
2 reported
q25_reported_12m
3 discrepancy between observation and report
q25_discrepancy_12m

195) When you are dressing your baby, is he/she pushes his arm to past the sleeves when the arm has reached the hole sleeves?
q26_push_sleeves_12m
1 yes
2 sometimes
0 no

196) Observed/ reported
1 observed
q26_observed_12m
2 reported
q26_reported_12m
3 discrepancy between observation and report
q26_discrepancy_12m

197) When you stretch out your hand and ask for his/her toy, does your baby to give it to your hand?
q27_give_toys_12m
1 yes
2 sometimes

0 no

198) Observed/ reported

1 observed
q27_observed_12m
2 reported
q27_reported_12m
3 discrepancy between observation and report
q27_discrepancy_12m

199) When you put on pants, socks, or shoes to your baby, does he/she lift his/her legs?

q28_pants_lift_legs_12m
1 yes
2 sometimes
0 no

200) Observed/ reported

1 observed
q28_observed_12m
2 reported
q28_reported_12m
3 discrepancy between observation and report
q28_discrepancy_12m

201) Does your baby rotate or throw back the ball to you? Hence, you can give it back to your child.

q29_throw_back_ball_12m
1 yes
2 sometimes
0 no

202) Observed/ reported

1 observed
q29_observed_12m
2 reported
q29_reported_12m
3 discrepancy between observation and report
q29_discrepancy_12m

203) Does your child play with his/her dolls or pet and hug it ?

q30_hug_dolls_pet_12m
1 yes
2 sometimes
0 no

204) Observed/ reported

1 observed
q30_observed_12m
2 reported

q30_reported_12m
3 discrepancy between observation and report
q30_discrepancy_12m

GENERAL
Parents or related parties can fill in the fields below for more information.

205) Does your baby use both hands and feet good and balanced? If not, please explain

206) When you help your baby to stand, do his feet more often tread? If not, please explain

207) Are you worried because your baby is too quiet or not making noises like other babies? If yes, please explain:

208) Do parents have a family history of hearing loss or deafness? If yes, please explain:

209) Are you worried about your baby's vision? If yes, please explain:

210) Has your baby been experiencing medical problems in the last few months? If yes, please explain:

211) Are you worried about your baby's behaviour? If yes, please explain:

212) Are there certain things you are worried about your baby? If yes, please explain:

WASH (Water, Sanitation, and Hygiene) Projects

ELIGIBILITY

This form must be completed at the time of the first visit

ID : ______

Interviewer : ___________________________

Mother's name : ___________________________

Child's name : ___________________________

Date Interview(dd/mm/yyyy) : ___/___/______

1. Children aged 54-66 months (Born 1 April 2014 until 29 September 2013):

   Yes O No O

2. Following the Baseline Research in 2014 :

   Yes O No O

3. Live in Tanjungsari/Pamulihan/Sukasari Subdistricts:

   Yes O No O

4. Have experienced changes in water sources at home since 2014 until now? :

   Yes O No O→ to question 6

5. If "Yes" Since When ______________:

6. Have experienced changes in drinking water sources since 2014 until now?

   Yes O→ to question 7 No O

7. If "Yes" Since when?__________

If one of the answer from question 1-3 is "No", then the mother and toddler can't participate in this study.

Hasil : 1) Can participate

2) Can't participate

3) Refuse

If 3) write the reasons here:

____

WASH (Water, Sanitation, and Hygiene) Projects

ANTHROPOMETRY QUESTIONNAIRE

Interviewer		
Date Measurement (dd/mm/yyyy)		
Measurement location		
ID		
Mother Name		
Child Name		**M/F**
Date of birth child		

Anthropometry Measurements of Toddlers.
Always do two measurements. Make third measurement if the difference between the first and second measurements exceeds the allowed difference (AD).

	Observation 1	Observation 2	Observation 3
Weight (Kg)<br>AD = 0.1 kg			
Height (cm)<br>AD = 0.5 cm			

**WAZ**	**HAZ**	**BMIZ**

Anthropometry Measurements of Mothers.
Always do two measurements. Make third measurement if the difference between the first and second measurements exceeds the allowed difference (AD).

	Observation 1	Observation 2	Observation 3
Weight (Kg)<br>AD=0.1 kg			
Height (Cm)<br>AD=0.5 cm			
Waist circumference			
Hip circumference			
Body Mass Index			

Ages & Stages Questionnaires (60 Month)

Interviewer : _____ Mother Name : ____________
ID : _____ Child Name : ____________

COMMUNICATION

No	Question	Yes	Sometimes	Not Yet	Score
1	Without your giving help by pointing or repeating directions, does your child follow three directions that are unrelated to one another? Give all three directions before your child starts. For example, you may ask your child, "Clap your hands, walk to the door, and sit down," or "Give me the pen, open the book, and stand up."				
2	Does your child use four- and five-word sentences? For example, does your child say, "I want the car"? Please write an example:				
3	When talking about something that already happened, does your child use words that end in "-ed," such as "walked," "jumped," or "played"? Ask your child questions, such as "How did you get to the store?" ("We walked.") "What did you do at your friend's house?" ("We played.") Please write an example:				
4	Does your child use comparison words, such as "heavier," "stronger," or "shorter"? Ask your child questions, such as "A car is big, but a bus is ____ " (bigger); "A cat is heavy, but a man is ____" (heavier); "A TV is small, but a book is ____" (smaller). Please write an example:				
5	Does your child answer the following questions? (Mark "sometimes" if your child answers only one question.)<br><br>"What do you do when you are hungry?" (Acceptable answers include "get food," "eat," "ask for something to eat," and "have a snack.") Please write your child's response:<br><br>What do you do when you are tired?" (Acceptable answers include: "take a nap," "rest," "go to sleep," "go to bed," "lie down," and "sit down.") Please write your				

	child's response:				
6	Does your child repeat the sentences shown below back to you, with- out any mistakes? (Read the sentences one at a time. You may repeat each sentence one time. Mark "yes" if your child repeats both sen- tences without mistakes or "sometimes" if your child repeats one sen- tence without mistakes.)<br><br>Jane hides her shoes for Maria to find.<br><br>Al read the blue book under his bed.				

GROSS MOTORIC

No	Question	Yes	Sometimes	Not yet	Score
1	While standing, does your child throw a ball overhand in the direction of a person standing at least 6 feet away? To throw overhand, your child must raise his arm to shoulder height and throw the ball forward. (Dropping the ball or throwing the ball underhand should be scored as "not yet.") 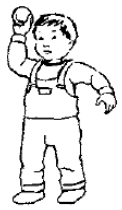				
2	Does your child catch a large ball with both hands? (You should stand about 5 feet away and give your child two or three tries before you mark the answer.) 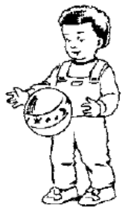				
3	Without holding onto anything, does your child stand on one foot for at least 5 seconds without losing her balance and putting her foot down? (You may give your child two or three tries before you mark the answer.) 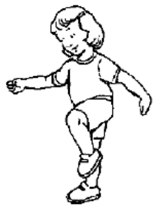				
4	Does your child walk on his tiptoes for 15 feet (about the length of a large car)? (You may show him how to do this.)				
5	Does your child hop forward on one foot for a distance of 4–6 feet without putting down the other foot? (You may give her two tries on each foot. Mark "sometimes" if she can hop on one foot only.)				
6	Does your child skip using alternating feet? (You may show him how to do this.)				

FINE MOTORIC

No	Question	Yes	Sometimes	Not yet	Score
1	Ask your child to trace on the line below with a pencil. Does your child trace on the line without going off the				

	line more than two times? (Mark “sometimes” if your child goes off the line three times.)				
2	Ask your child to draw a picture of a person on a blank sheet of paper. You may ask your child, “Draw a picture of a girl or a boy.” If your child draws a person with head, body, arms, and legs, mark “yes.” If your child draws a person with only three parts (head, body, arms, or legs), mark “sometimes.” If your child draws a person with two or fewer parts (head, body, arms, or legs), mark “not yet.” Be sure to include the sheet of paper with your child’s drawing with this questionnaire.				
3	Draw a line across a piece of paper. Using child-safe scissors, does your child cut the paper in half on a more or less straight line, making the blades go up and down? (Carefully watch your child’s use of scissors for safety reasons.) 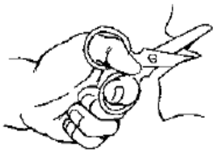				
4	Using the shapes below to look at, does your child copy the shapes in the space below without tracing? (Your child’s drawings should look similar to the design of the shapes below, but they may be different in size. Mark “yes” if she copies all three shapes; mark “sometimes” if your child copies two shapes.)<br>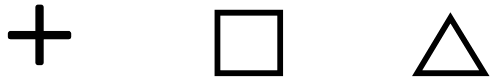<br>(Space for child’s shapes)				
5	Using the letters below to look at, does your child copy the letters without tracing? Cover up all of the letters except the letter being copied. (Mark “yes” if your child copies four of the letters and you can read them. Mark “sometimes” if your child copies two or three letters and you can read them.)<br>**V H T C A**<br>(*Space for child’s letters*)				
6	Print your child’s first name. Can your child copy the letters? The letters may be large, backward, or reversed. (Mark “sometimes” if your child copies about half of the letters.)<br>(*Space for adult’s printing*)				

PROBLEM SOLVING

No	Question	Yes	Sometimes	Not yet	Score
1	When asked, “Which circle is smallest?” does your child point to the smallest circle? (Ask this question without providing help by pointing, gesturing, or looking at the smallest circle.)<br>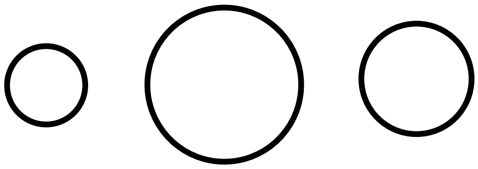				
2	When shown objects and asked, “What color is this?” does your child name five different colors like red, blue, yellow, orange, black, white, or pink? (Mark “yes” only if your child answers the question correctly using five colors.)				
3	Does your child count up to 15 without making mistakes? If so,<br>mark “yes.” If your child counts to 12 without making mistakes, mark “sometimes.”				
4	Does your child finish the following sentences using a word that means the opposite of the word that is italicized? For example: “A rock is hard, and a pillow is soft.”<br><br>Please write your child’s responses below:<br><br>A cow is big, and a mouse is<br><br>Ice is cold, and fire is<br><br>We see stars at night, and we see the sun during the<br><br>When I throw the ball up, it comes<br><br>(Mark “yes” if he finishes three of four sentences correctly. Mark “sometimes” if he finishes two of four sentences correctly.)				
5	Does your child know the names of numbers? (Mark “yes” if she identi- fies the three numbers below. Mark “sometimes” if she identifies two numbers.)<br>**3 1 2**				
6	Does your child name at least four letters in her name? Point to the letters and ask, “What letter is this?” (Point to the letters out of order.)				

PERSONAL SOCIAL

No	Question	Yes	Sometimes	Not yet	Score
1	Can your child serve himself, taking food from one container to another, using utensils? For example, does your child use a large spoon to scoop applesauce from a jar into a bowl?				
2	Does your child wash her hands and face using soap and water and dry off with a towel without help?				
3	Does your child tell you at least four of the following? Please mark the items your child knows.<br>○ A. First name<br>○ B. Age<br>○ C. City he lives in<br>○ D. Last name<br>○ D. Boy or Girl<br>○ E. Telephone number				
4	Does your child dress and undress himself, including buttoning medium-size buttons and zipping front zippers?				
5	Does your child use the toilet by herself? (She goes to the bathroom, sits on the toilet, wipes, and flushes.) Mark "yes" even if she does this after you remind her.				
6	Does your child usually take turns and share with other children?				

OVERALL QUESTIONS

1. *Do you think your child hears well? If no, explain::*..
....
.....

2. *Do you think your child talks like other children her age? If no, explain*……………………………….
....
.....

3. *Can you understand most of what your child says? If no, explain*..
....
.....

4. *Can other people understand most of what your child says? If no, explain*……………………….
....
....

5. *Do you think your child walks, runs, and climbs like other children his age? If no, explain:*……….
....
.....

6. *Does either parent have a family history of childhood deafness or hearing impairment? If yes, explain*..
....
....
.....

7. *Do you have any concerns about your child's vision? If yes, explain*:..
....
...

8. *Has your child had any medical problems in the last several months? If yes, explain*:................
....
....

9. *Do you have any concerns about your child's behavior? If yes, explain*………………….............
....
...

10. *Does anything about your child worry you? If yes, explain*...
....
..

WASH (Water, Sanitation, and Hygiene) Projects

HOME & ENVIRONMENT SANITATION OBSERVATION

Interviewer : ……………………………………….
Date Interview (dd:mm:yyyy): ……………………………………….
ID : ……………………………………….
Mother's Name : ……………………………………….
Toddler's Name : ……………………………………….

HOME OBSERVATION

1. What type of water source is most used for household use?
   (1) Piped water (5) Unprotected dug well (9) River/stream
   (2) Buy clean water (6) Protected spring (10) Other ______________
   (3) well/pump water* (7) Unprotected spring
   (4) Protected dug well** (8) Rainwater
   *depth > 20 meter
   **depth 5-15 meter

2. What type of drinking water source is most used for household use?
   (1) Bottled water (branded) (6) Tube well or borehole (11) Rainwater
   (2) Gallon (branded) (7) Protected dug well (12) River/laku/irrigation
   (3) Non-branded refilled water (8) Unprotected dug well (13) Other _______________
   (4) Piped water into dwelling (9) Protected spring
   (5) tanker truck/cart (10) Unprotected spring

3. If number 2 = 6 - 10 (pump/dug well/spring) how far the distance from the nearest fecal disposal?
   (1) <10 metre
   (2) >= 10 metre
   (3) Don't know

4. Is water for drinking easily obtained throughout the year?
   (1) Yes (easily) → go to question 6
   (2) Difficult in dry season
   (3) Difficult throughout the year

5. If "difficult in dry season/difficult throughout the year" how to supply drinking water for household?
   ____

6. Do you treat your water to make it safer to drink?
   (1) Boiled
   (2) Using UV light
   (3) Chlorine tablets
   (4) Dispenser with heater and cooler
   (5) Filtration
   (6) Other treatment : __________________
   (7) Without treatment

7. What type of water storage for drinking water?
   (1) Dispenser
   (2) teapot/jerry can
   (3) jug
   (4) Bucket/covered pot
   (5) Other______________________

8. What type of toilet facilities do members of your household use?
   (1) Own household → **go to question 10**
   (2) Shared toilet
   (3) Public toilet
   (4) No Facilities

9. If "Shared toilet/Public toilet" how many households use this toilet facilities?
   (1) <10
   (2) >10
   (3) Don't know

10. What type of toilet in your household?
    (1) Water sealed latrine
    (2) Pit latrine
    (3) Overhung latrine
    (4) No toilet facility
    (5) Other __________

11. What type of fecal disposal in your household?
    (1) Septic Tank
    (2) Piped sewer system*
    (3) Pool/rice field
    (4) River/Lake/Sea
    (5) Ground hole
    (6) Beach/Field/farm
    (7) Other __________________
    *disposal waste transferred to a typical shelter

12. Waste water disposal from bathroom/kitchen/wastafel
    (1) Piped sewer system
    (2) waste water shelter in yard
    (3) open waste water shelter in yard
    (4) waste water shelter outside the yard
    (5) no facilities/open space or field → **go to question 14**

(6) Sewer/river → **go to question 14**
(7) Other _______________________ → **go to question 14**

13. Do you share this water disposal facility with other households?
(1) Own Household
(2) Communal
(3) Other ______________

14. Is your home near any industrial areas?
(1) Yes, explain ____________ → **go to question 15**
(2) Tidak

15. How far is it between your home and the industrial areas?
(1) <11 m
(2) 11 – 50 m
(3) >50 m

WASH (Water, Sanitation, and Hygiene) Projects
Water and Tissue Collection

ID : ___ Mother's name : ______________ Child's Name : ________________

I. Water Collection	
Field worker	
Date of water collection (dd/mm/yy)	_____ /_____ /_____
Time of water collection (hh:mm)	____:____
	Time (hh:mm)
Time of water storage in coolbox (hh:mm)	____:____
Time of water storage in lab (hh:mm)	____:____

II. Container Stool Collection	
Field worker	
Date of stool collection (dd/mm/yy)	_____ /_____ /_____
Time of stool collection (hh:mm)	____:____
Time of stool storage in container (hh:mm)	____:____
Date of defecate (dd/mm/yy)	_____ /_____ /_____
Time of defecate (hh:mm)	____:____

II. Microtube Stool Collection		
Field worker		
Date of stool collection (dd/mm/yy)	_____ /_____ /_____	
Time of stool collection (hh:mm)	____:____	
	Time	Temperature
Time of stool storage in container (hh:mm)	____:____	
Time of stool storage in biofreeze (hh:mm)	____:____	
Time of stool storage in freezer (hh:mm)	____:____	
Date of defecate (dd/mm/yy)	_____ /_____ /_____	
Time of defecate (hh:mm)	____:____	

WASH (Water, Sanitation, and Hygiene) Projects

SANITATION & HYGIENE QUESTIONNAIRES

Interviewer : ____________________
Date of Interview : ____________________
ID number : ____________________
Mother's name : ____________________
Child's name : ____________________

HYGIENE INDEX

A. Drinking Water Index (DWI)

Parameter	Yes	No	Skor
Interior water container is covered			
Exterior water container is clean			
Pila contains water*			

**A pila is general use water storage container*

B. Food Index (FI)

Parameter	Yes	No	Skor
Clean dishes are covered			
Clean dishes are stored high			
All food is covered			

C. Personal Hygiene Index (PHI)

Parameter	Yes	No	Skor
Mother is wearing shoes			
Mother's hands are clean :			
Before eating			
After going to the toilet			
Before handling the child			
After changing diaper			
Before preparing food			
After touching animal			
Index child's hands are clean :			
Cutting nails (once /week)			
Nails are not long			

Washes hand using soap			

D. Household Index (HHI)

Parameter	Yes	No	Skor
No trash outside house			
No trash inside house			
No unrestrained animal in patio or house			
No accumulation of dirty clothes			
Insignificant number of flies in house			
No standing water in patio or around house			

E. Summary Hygiene Index

Parameter	Skor
Drinking Water Index (DWI)	
Food Index (FI)	
Personal Hygiene Index (PHI)	
Household Hygiene Index (HHI)	
Total score	

Cross-cultural study of household water insecurity

Interviewer__________________

Date __________________________

Time at start of interview _______

Time at end of interview: __________________________

Place of Interview______________

Region: ____________

Participant ID : ________________

Participant Name __________________________

Participant gender : (0) Male (1) Female

Participant place of residence : (0) Rural (2) Peri-urban (3) Urban

Participant Ethnicity:

Participant ID should start with 1st two letters of country, eg. BA, NE, GU, GH…

1. Screening Questions

Code Name	Question	Coding Classification
SQ1a	Do you agree to participate in this survey?	0…No<br>1…Yes
SQ2	Are you 16 years of age or older?	0…No<br>1…Yes
SQ3	Would you consider yourself knowledgeable about water acquisition and use within your household?	0…No<br>1…Yes
SQ4	If no, who is most knowledgeable about water acquisition and use within your household? Who should I talk to?	

2. Socio Demographic Questions

SD1	Role in household: What is your relationship to the head of your household? By household, I mean all people who sleep under the same roof and take food from the same pot.	1….Self<br>2….Spouse/partner<br>3….Adult Child<br>4….Other
SD2	What is the gender of household head?	0….Male<br>1….Female
SD3	What is your current relationship status?	1…. Single/separated or divorced<br>2…. Widowed<br>3…. Have a partner who lives separately<br>4…. Have a partner who lives with you<br>5…. Married
SD4	How old are you?	

SD5	Who is primarily responsible for making sure there is enough water in the house? By household, I mean all people who sleep under the same roof and take food from the same pot.<br><br>*If primary responsibility is shared, choose (5) and then also circle the two categories of people who share the responsibility.*	1….Self<br>2….Spouse/partner<br>3….Children<br>4….Other family members<br>5….Shared responsibility
SD6	How many children (≤16 years) live in your household? By household, I mean all people who sleep under the same roof and take food from the same pot.	
SD7	How many adults (>16 years) including yourself live in your household? By household, I mean all people who sleep under the same roof and take food from the same pot.	
SD8	What type of housing do you live in?	1…. House/condominium (owned)<br>2…. House/condominium (rented)<br>3…. Apartment (owned)<br>4…. Apartment (rented)<br>5…. Farm (own)<br>6…. Farm (lease)<br>7…. Informal settlement/squatter community<br>8…. Refugee/internally displaced person camp<br>9….Other

3. Household Water Insecurity Experiences Scale

Now I'm going to ask you about your own personal experiences with water. For each of the items, please indicate how many times within the past 4 weeks or 30 days. Interviewer, please mark the response that best corresponds with the coding classification. For example, if participants says 15 times, you need to mark/circle code 4.

Code name	Question	Coding Classification

HWISE1	In the last 4 weeks, how frequently did you or anyone in your household **worry** you would not have enough water for all of your household needs?	1… Never (0 times in the last 4 weeks)<br>2… Rarely (1–2 times in the last 4 weeks)<br>3… Sometimes (3–10 times in the last 4 weeks)<br>4… Often (11-20 times in the last 4 weeks)<br>5… Always (More than 20 times in the last 4 weeks)<br>99…Don't know<br>88…Not applicable/I don't have this
HWISE2	In the last 4 weeks, how frequently has your household water supply from your main water source been **interrupted** or **limited** (e.g. water pressure, less water than expected)?	1… Never (0 times in the last 4 weeks)<br>2… Rarely (1–2 times in the last 4 weeks)<br>3… Sometimes (3–10 times in the last 4 weeks)<br>4… Often (11-20 times in the last 4 weeks)<br>5… Always (More than 20 times in the last 4 weeks)<br>99…Don't know<br>88…Not applicable/I don't have this
HWISE3	In the last 4 weeks, how frequently has there not been enough water in the household to **wash clothes**?	1… Never (0 times in the last 4 weeks)<br>2… Rarely (1–2 times in the last 4 weeks)<br>3… Sometimes (3–10 times in the last 4 weeks)<br>4… Often (11-20 times in the last 4 weeks)<br>5… Always (More than 20 times in the last 4 weeks)<br>99…Don't know<br>88…Not applicable/I don't have this
HWISE4	In the last 4 weeks, how frequently has you or anyone in your household had to **change schedules/plans** due to problems with your water situation, such as problems getting or distributing water within the household? Activities that may have been interrupted include caring for others, doing household chores, etc.	1… Never (0 times in the last 4 weeks)<br>2… Rarely (1–2 times in the last 4 weeks)<br>3… Sometimes (3–10 times in the last 4 weeks)<br>4… Often (11-20 times in the last 4 weeks)<br>5… Always (More than 20 times in the last 4 weeks)<br>99…Don't know<br>88…Not applicable/I don't have this
HWISE5	In the last 4 weeks, how frequently have you or anyone in your household had to **change what was being eaten** because there were problems with water (e.g. for washing foods, cooking, etc.)?	1… Never (0 times in the last 4 weeks)<br>2… Rarely (1–2 times in the last 4 weeks)<br>3… Sometimes (3–10 times in the last 4 weeks)<br>4… Often (11-20 times in the last 4 weeks)<br>5… Always (More than 20 times in the last 4 weeks)<br>99…Don't know<br>88…Not applicable/I don't have this

HWISE6	In the last 4 weeks, how frequently have you or anyone in your household had to go **without washing hands** after **dirty activities (**e.g., defecating or changing diapers, cleaning animal dung) because of problems with water?	1… Never (0 times in the last 4 weeks)<br>2… Rarely (1–2 times in the last 4 weeks)<br>3… Sometimes (3–10 times in the last 4 weeks)<br>4… Often (11-20 times in the last 4 weeks)<br>5… Always (More than 20 times in the last 4 weeks)<br>99…Don't know<br>88…Not applicable/I don't have this
HWISE7	In the last 4 weeks, how frequently have you or anyone in your household had to go without **washing their body** because of problems with water (e.g. not enough water, dirty, unsafe)?	1… Never (0 times in the last 4 weeks)<br>2… Rarely (1–2 times in the last 4 weeks)<br>3… Sometimes (3–10 times in the last 4 weeks)<br>4… Often (11-20 times in the last 4 weeks)<br>5… Always (More than 20 times in the last 4 weeks)<br>99…Don't know<br>88…Not applicable/I don't have this
HWISE8	In the last 4 weeks, how frequently has there not been **as much water to drink** as you would like for you or anyone in your household?	1… Never (0 times in the last 4 weeks)<br>2… Rarely (1–2 times in the last 4 weeks)<br>3… Sometimes (3–10 times in the last 4 weeks)<br>4… Often (11-20 times in the last 4 weeks)<br>5… Always (More than 20 times in the last 4 weeks)<br>99…Don't know<br>88…Not applicable/I don't have this
HWISE9	In the last 4 weeks, how frequently did you or anyone in your household feel **angry** about your water situation?	1… Never (0 times in the last 4 weeks)<br>2… Rarely (1–2 times in the last 4 weeks)<br>3… Sometimes (3–10 times in the last 4 weeks)<br>4… Often (11-20 times in the last 4 weeks)<br>5… Always (More than 20 times in the last 4 weeks)<br>99…Don't know<br>88…Not applicable/I don't have this
HWISE10	In the last 4 weeks, how frequently have you or anyone in your household gone to **sleep thirsty** because there wasn't any water to drink?	1… Never (0 times in the last 4 weeks)<br>2… Rarely (1–2 times in the last 4 weeks)<br>3… Sometimes (3–10 times in the last 4 weeks)<br>4… Often (11-20 times in the last 4 weeks)<br>5… Always (More than 20 times in the last 4 weeks)<br>99…Don't know<br>88…Not applicable/I don't have this

HWISE11	In the last 4 weeks, how frequently has there been **no useable or drinkable water** whatsoever in your household?	1… Never (0 times in the last 4 weeks)<br>2… Rarely (1–2 times in the last 4 weeks)<br>3… Sometimes (3–10 times in the last 4 weeks)<br>4… Often (11-20 times in the last 4 weeks)<br>5… Always (More than 20 times in the last 4 weeks)<br>99…Don't know<br>88…Not applicable/I don't have this
HWISE12	In the last 4 weeks, how frequently have problems with water caused you or anyone in your household to **feel ashamed**/excluded/stigmatized?	1… Never (0 times in the last 4 weeks)<br>2… Rarely (1–2 times in the last 4 weeks)<br>3… Sometimes (3–10 times in the last 4 weeks)<br>4… Often (11-20 times in the last 4 weeks)<br>5… Always (More than 20 times in the last 4 weeks)<br>99…Don't know<br>88…Not applicable/I don't have this

WASH (Water, Sanitation, and Hygiene) Projects

SANITATION & HYGIENE QUESTIONNAIRES

Interviewer : ____________________

ID number : ____________________

1. Has your child had diarrhea in the last 2 weeks (diarrhea if you have loose stools three or more times in one day and more liquid)?
   a. Yes
   b. No
2. Has your member of household had diarrhea in the last 2 weeks?
   a. Yes
   b. No
3. Has your child been ill with a fever in the last 2 weeks?
   a. Yes
   b. No

4. Has your child had an illness with a cough in the last 2 weeks?
   a. Yes
   b. No → Go to question 6

5. When your child had an illness with a cough, did he/she breathe faster than usual with short, rapid breaths or have difficulty breathing?
   a. Yes
   b. No

6. Has your child been hospitalized?
   a. Yes
   b. No

7. If yes, explain why :
   ____

8. Has your child been given worm medicine in heatlh center /medical personnel?
   a. Yes
   b. No → go to question 10
   c. Don't know
9. If Yes, when the last time your child been given worm medicine?
   ____

10. Has your child been consume worm medicine in last 6 month?
    a. Yes
    b. No
    c. Don't know

11. If Yes, where did you get that?

____
_

12. Has your child consume antibiotics medicine in last 2 weeks
    a. Yes
    b. No

13. if Yes, what’s the name of antibiotics?

____

14. Has your child consume fortified biscuits or crackers?
    a. Yes
    b. No

15. if yes, explain what the name of biscuit/crackers and frequency consume of biscuits/crackers

____

ID :…………………
Date of observation :…………………

WASHING HAND OBSERVATION

Step	Checklist (√)
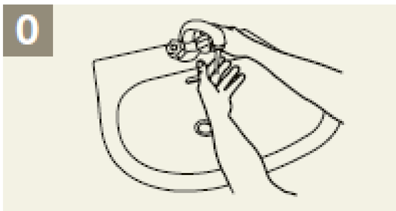 Wet hands with water;	
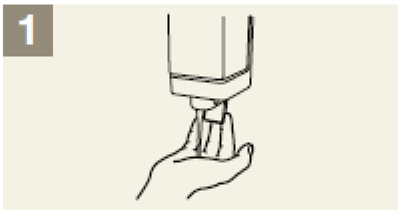 Apply enough soap to cover all hand surfaces;	
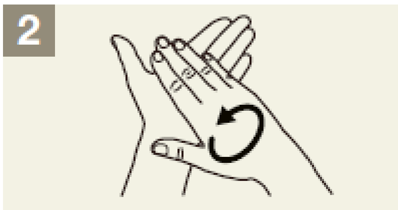 Rub hands palm to palm;	
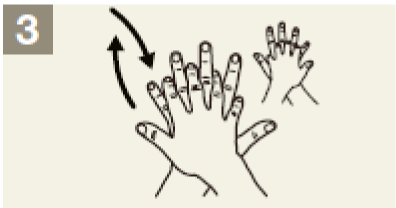 Right palm over left dorsum with interlaced fingers and vice versa;	
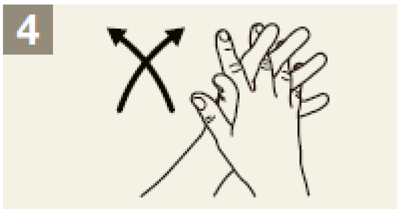 Palm to palm with fingers interlaced;	

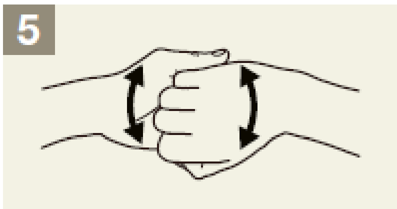<br>Backs of fingers to opposing palms with fingers interlocked;	
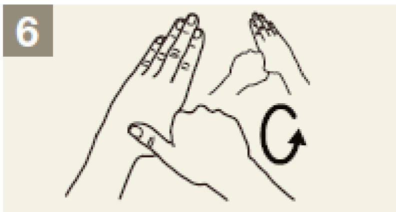<br>Rotational rubbing of left thumb clasped in right palm and vice versa;	
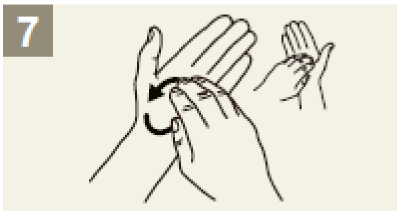<br>Rotational rubbing, backwards and forwards with clasped fingers of right hand in left palm and vice versa;	
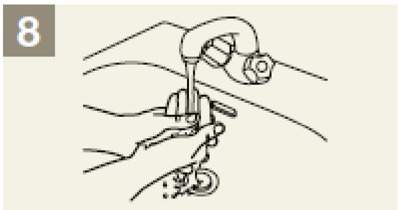<br>Rinse hands with water;	
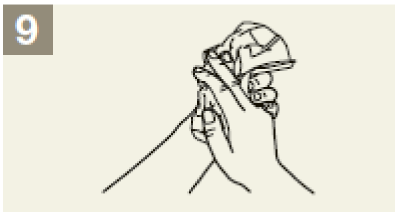<br>Dry hands thoroughly with a single use towel;	
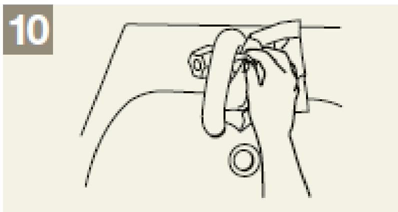<br>Use towel to turn off faucet;	

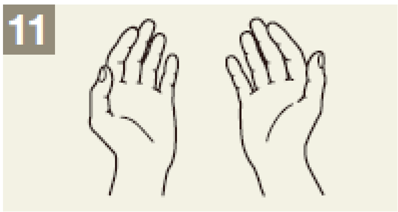Your hands are now safe.	

WASH (Water, Sanitation, and Hygiene) Projects

Children's Health Status

Interviewer : ___________________

ID : ___________________

Characteristics of Children and Mothers

1. How many child do you have:
   a. 1 child
   b. 2 children
   c. 3 children
   d. More than 3 children
2. Child who participated in this project is the child number:
   a. First child
   b. Second child
   c. Third child
   d. Fourth child
3. Do you have KIA book?
   a. Yes
   b. No
4. Do you like to read the KIA book?
   a. Yes
   b. No
5. Do you feel the benefits of the KIA book?
   a. Yes, state the benefits …………………………………………………......
      ………………………………………………………………………………
   b. No

Description of disease patterns

1. Did your child sick in the last 1 month?
   a. Yes
   b. Never
2. If yes, what are the main symptoms?
   a. Diarrhea
   b. Fever
   c. Coughs and colds
   d. Other : ………………………………
3. How many times your child has been sick in last one month?
   a. 1 time
   b. 2 times
   c. 3 times
   d. More than 3 times
4. How many days does your child suffer from illness usually?
   a. 2 – 3 days

b. 4 – 5 days
c. 6 – 7 days
d. More than 7 days

5. When your child is gettng sick, did you take your child to the doctor or health center?
   a. Yes
   b. No
6. Do you know that your child’s teeth have dental caries?
   a. Yes
   b. No
7. Mark it (x) on the teeth which have caries

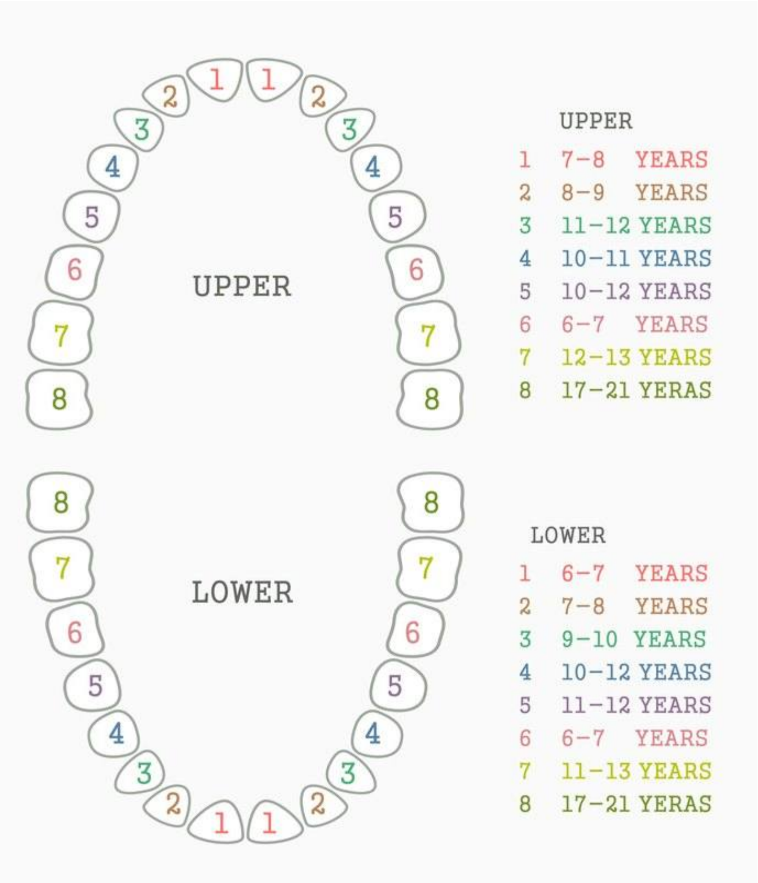

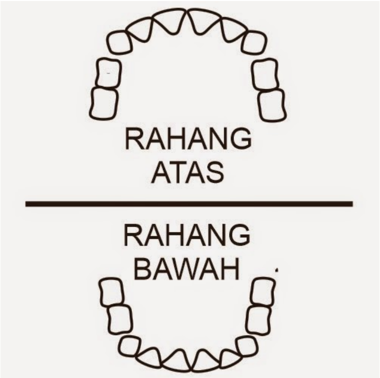

8. Does your child have brush the teeth routinely, morning and evening?
   a. Yes
   b. No
9. Do you regularly check your child’s teeth to a doctor?
   a. Yes
   b. No
10. Do you weigh your child every month?

a. Yes
b. No

11. Where do you weigh your child every month?
    a. Posyandu
    b. Puskesmas (Health Center)
    c. Clinic
    d. Pediatrician
    e. Doctor practice
    f. Midwife Practice
12. Until what age is your child routinely measured weight everymonth?
    a. Until my child is 6 months old
    b. Until my child is 12 months old
    c. Until my child is 2 years old
    d. Until my child is 3 years old
    e. Until my child is 4 years old
    f. Until my child is 5 years old
13. Did your child get the immunization according to the type of immunization in the table below:

No	Type of Immunization	Immunization Status*)
1	Hep B	
2	BCG	
3	Polio 1	
4	Polio 2	
5	Polio 3	
6	Polio 4	
7	Penta valent (DPT-HB-HIB) 1	
8	Penta valent (DPT-HB-HIB) 2	
9	Penta valent (DPT-HB-HIB) 3	
10	Penta valent (DPT-HB-HIB) 4	
11	Campak	

*) this column is filled with

V when the child get immunization

X if the child have not received the immunization
